# Supplementary material for: Characterized cis-FeV(O)(OH) intermediate mimics enzymatic oxidations in the gas phase
Source: Nat Commun. 2019 Feb 22;10:901. doi: 10.1038/s41467-019-08668-2 (PMC6385299; doi:10.1038/s41467-019-08668-2)
Supplement: Supplementary file 1 — Supplementary Information [file 41467_2019_8668_MOESM1_ESM.pdf]

Supplementary information for

Characterized *cis*-Fe^{V}(O)(OH) Intermediate Mimics Enzymatic Oxidations in the Gas Phase

Borrell *et al.*

Table of contents

Synthesis of the complex 1 3
Characterization of intermediate 2 3
Fig. 1 3
Fig. 2 4
Representative examples of catalytic *syn*-dihydroxylation reactions 4
Supplementary figures 5
Fig. 3 5
Fig. 4 6
Fig. 5 7
Fig. 6 8
Fig. 7 9
Table 1 10
Table 2 10
Supplementary Discussion 11
Fig. 8 11
Table 3 12
XYZ coordinates 13
References 28

Synthesis of the complex 1

The synthesis of the complex was synthetized following previously described procedure.[1]

Characterization of intermediate 2

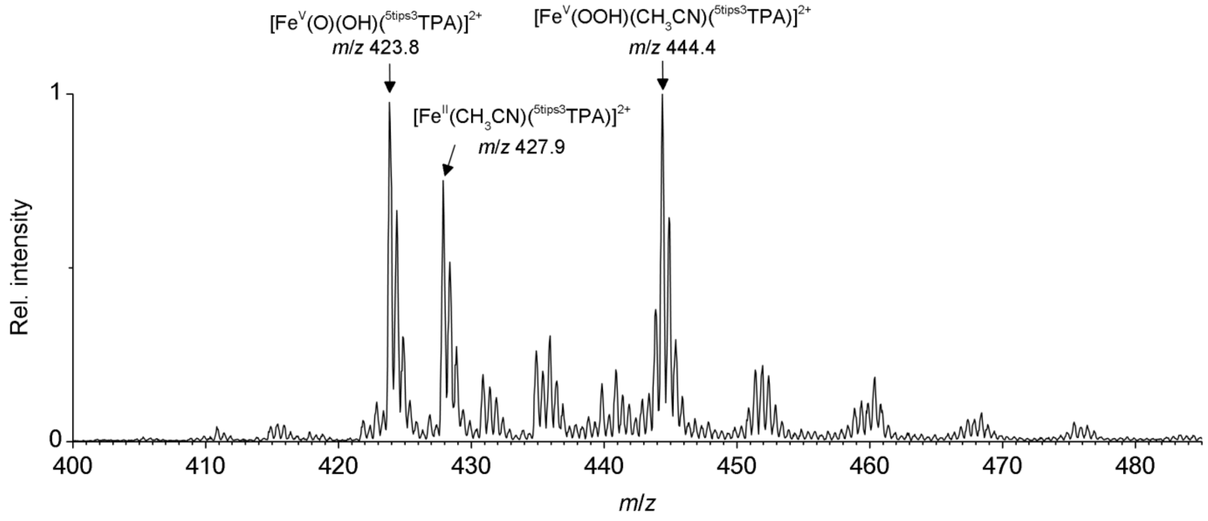

Fig. 1 Mass spectrum of the $^{5\text{-tips3}}$tpaFe^{III}OOH (**2**) solution.

A 0.4 mM solution of **1** in dry acetonitrile was prepared inside the glovebox. 2 mL of this solution were placed in a UV-Vis cuvette. The quartz cell was capped with a septum and taken out of the box, placed in the Unisoku cryostat of the UV-Vis spectrophotometer and cooled down to 233 K. After reaching thermal equilibrium, a UV-Vis spectrum of the starting complex was recorded. Then, 10 equiv. H_2O_2 in dry acetonitrile were added. The formation of a band at λmax = 544 nm was observed.

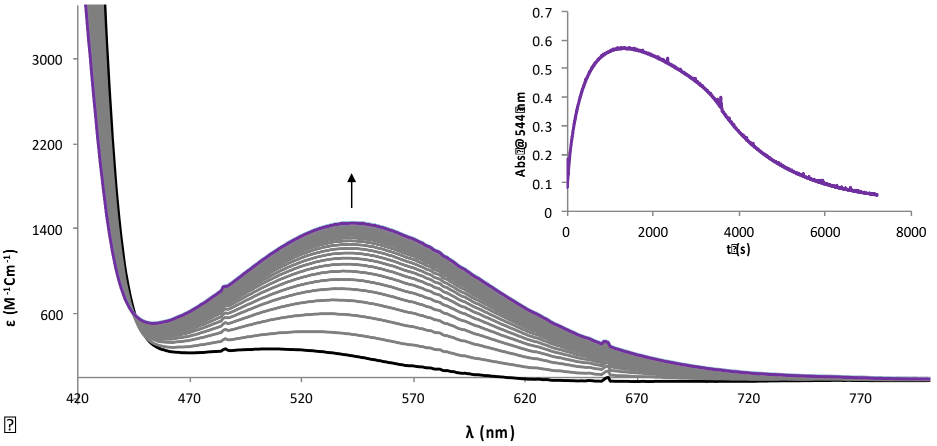

Fig. 2 UV-Vis spectra of **1** (0.4 mM, black line), **2** (violet line). Solid gray lines show the progressive formation of 2 upon addition of 10 equiv H_2O_2 at -40 °C in acetonitrile over the course of 126 minute. Inset: time trace at 544 nm.

Representative examples of catalytic *syn*-dihydroxylation reactions

General procedure:[1] An acetonitrile solution (1 mL) of substrate (90 µmol, 1 equiv), the catalyst (0.9 µmol, 1 mol%) and $Mg(ClO_4)_2{\cdot}6H_2O$ (198 µmol, 2.2 equiv.) was prepared in a 3 mL vial equipped with a stir bar, and the resulting mixture cooled with an ice bath. 114 µL (135 µmol, 1.5 equiv) of 0.58M H_2O_2 solution in acetonitrile (diluted from 50% in aqueous solution) were directly added by syringe pump over 30 minutes. Then, the solution was stirred for further 30 minutes. An internal standard (biphenyl) was added and the solution was immediately filtered through a short silica plug, which was subsequently rinsed with AcOEt. The reaction mixture was then analysed by GC and GC-MS. Products were identified and quantified by comparison with authentic samples.

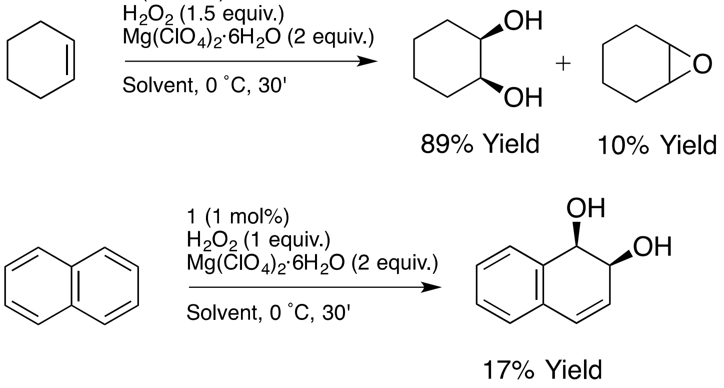

Supplementary figures

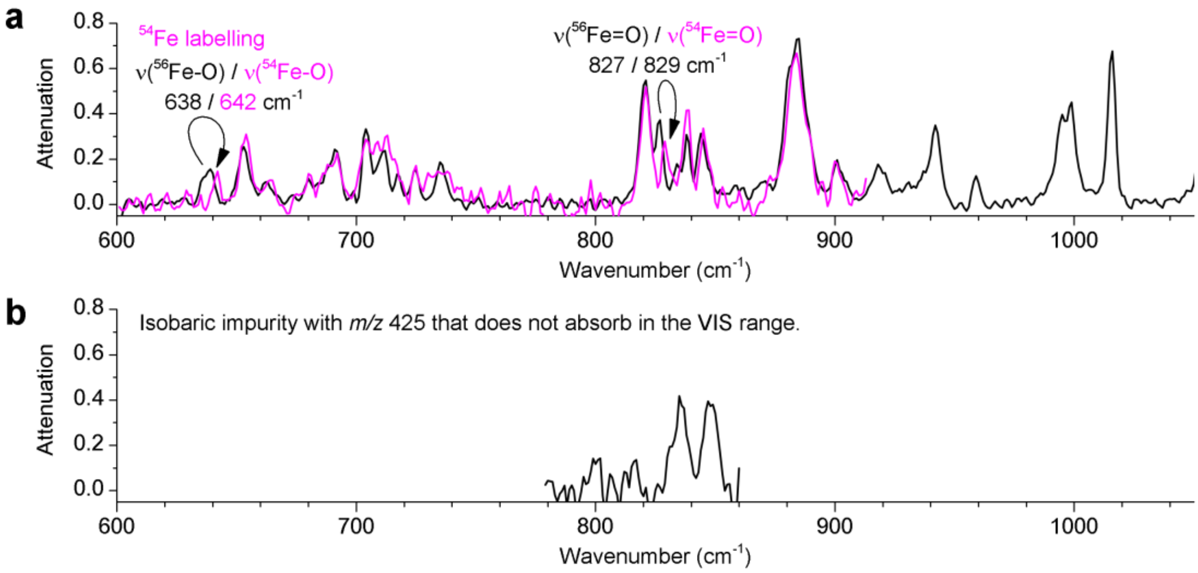

Fig. 3 (a) IRPD spectra of $\mathbf{3}^{2+}$ and $\mathbf{3}^{2+}$(^{54}Fe). (b) IRPD spectrum of an isobaric impurity present in $\mathbf{3}^{2+}$(^{16}O^{18}O) ions with *m*/*z* 425. The spectrum was obtained by depleting the helium clusters population by irradiation of the ion cloud with a visible laser concurrent with the IR irradiation.

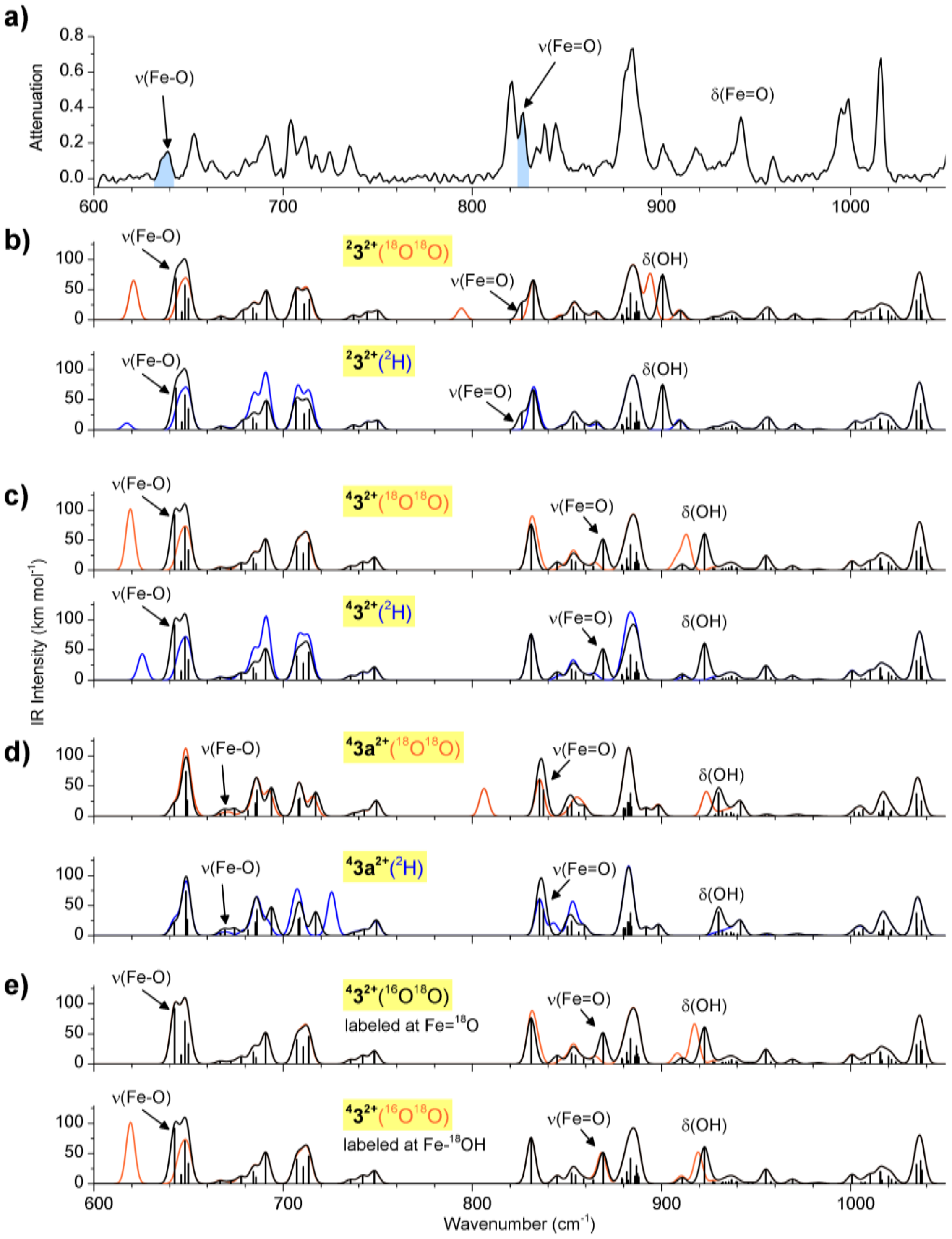

Fig. 4 (a) IRPD spectrum of $\mathbf{3}^{2+}$. (b-e) Theoretical predictions of the IR spectra of different isomers and isotopic labeling of $\mathbf{3}^{2+}$.

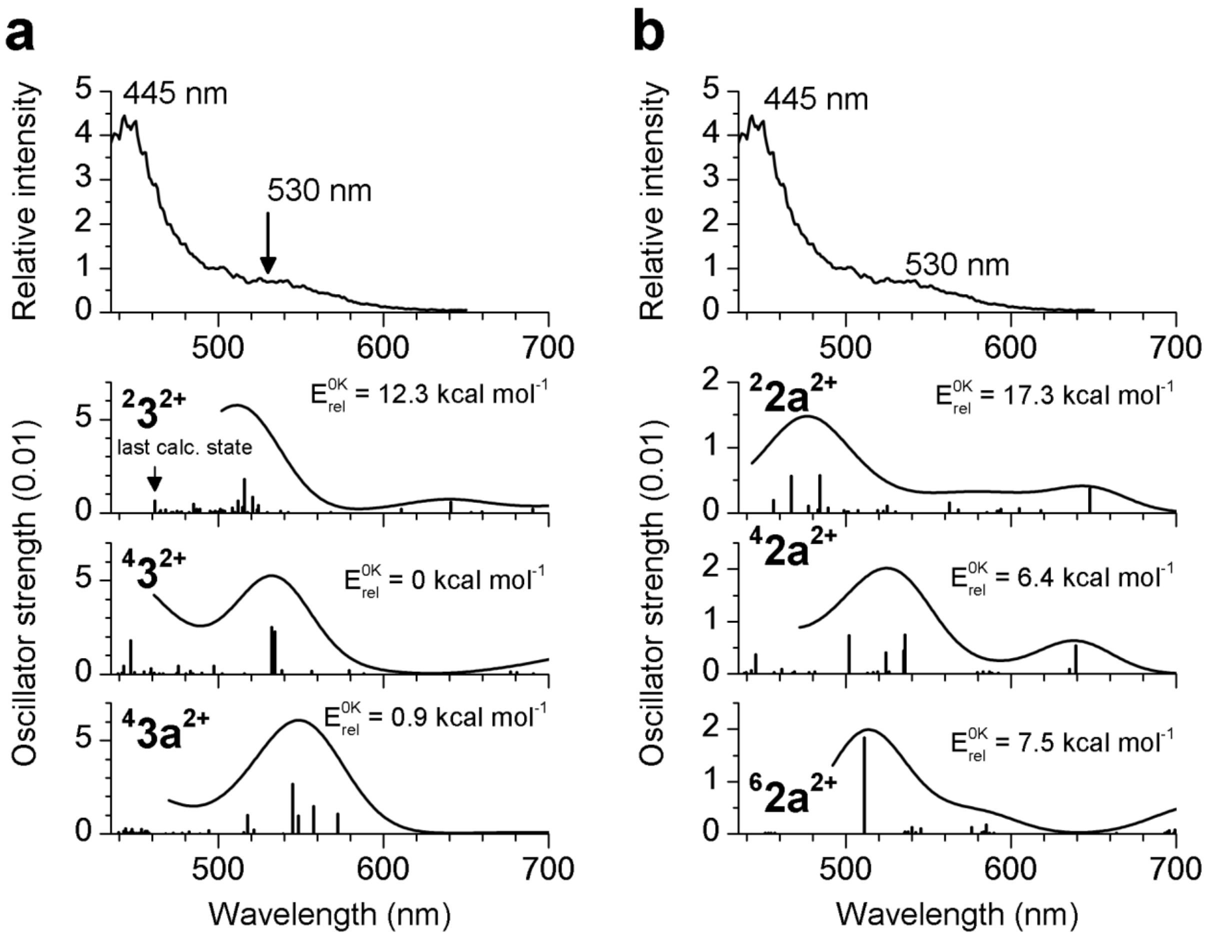

Fig. 5 Comparison of experimental VIS spectrum of **3** with TD-DFT predictions (B3LYP-D3/def2-TZVP). The convoluted spectrum is only shown to within 40 nm of the last predicted state (there were 64 states). Relative intensity was calculated by dividing the attenuation by the visible laser power in mW and irradiation time (0.780 s).

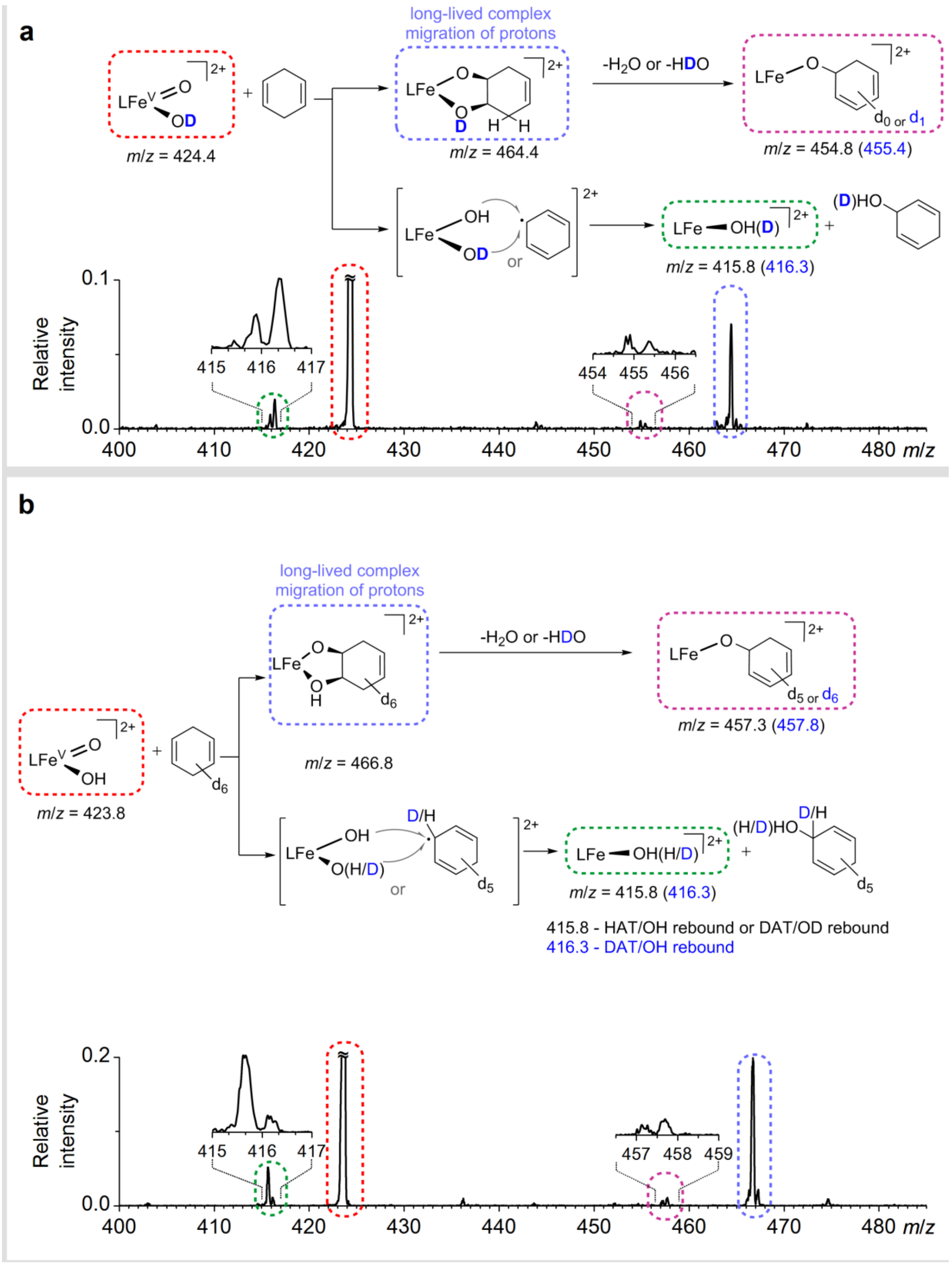

Fig. 6 Ion-molecule reactivity of $\mathbf{3}^{2+}$(^{2}H) in the gas phase with 0.1 mTorr of 1,4-cyclohexadiene. The reaction was measured at nominally zero-collision energy determined from the retarding potential analysis.

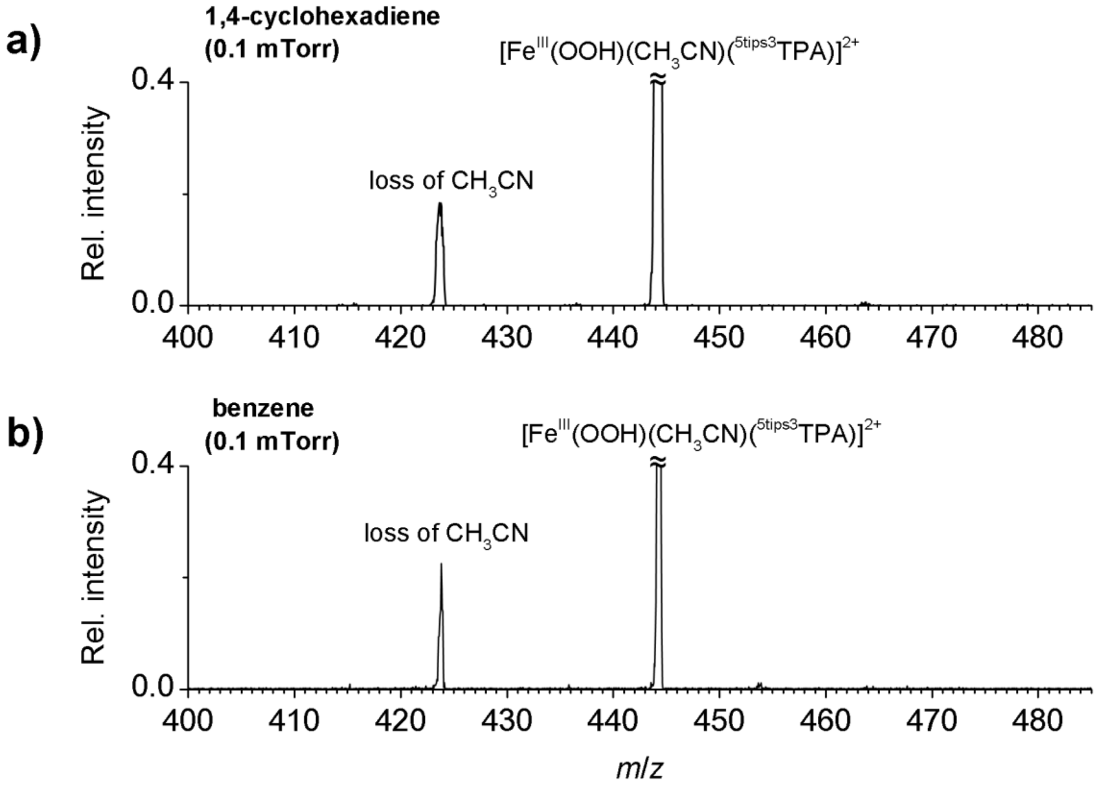

Fig. 7 Ion-molecule reactivity of $\mathbf{2}^{2+}$ in the gas phase with (a) 0.1 mTorr of 1,4-cyclohexadiene and (b) 0.1 mTorr of benzene. The reactions were measured at nominally zero-collision energy determined from the retarding potential analysis.

Table 1. Relative enthalpies of complexes **$2a^{2+}$** and **3^{2+}** and **$3a^{2+}$** in different spin states.

**Complex**	**Relative enthalpy at 0 K (kcal mol-1)[a]**
$^{2}2a^{2+}$	17.3
$^{4}2a^{2+}$	6.4
$^{6}2a^{2+}$	7.5
$^{2}3a^{2+}$	12.7
$^{2}3^{2+}$	12.3
$^{4}3a^{2+}$	0.9
$^{4}3^{2+}$	0

[a]The energies were calculated at B3LYP-D3/def2TZVP level and include zero-point vibrational energies at the same level of theory.

Table 2. Branching ratios between the addition reaction (*syn*-dihydroxylation) and the oxygen atom transfer reaction in the reactions of **3^{2+}** with olefins.

**Reaction**	**Addition**: +M/(+M-H_2O)/(+M-HDO) [%]	**OAT**: (+H,-OH)/(+D,-OH) [%]
**3^{2+}** + cyclohexene	**80**: 80/0	**20**
**3^{2+}**(^{2}H) + cyclohexene	**84**: 84/0	**16**: 15/1
**3^{2+}** + 1,4-cyclohexadiene	**69**: 63/7	**31**
**3^{2+}**(^{2}H) + 1,4-cyclohexadiene	**73**: 67/4/3	**27**: 19/7
**3^{2+}** + 1,3-cyclohexadiene	**77**: 71/6	**23**
**3^{2+}**(^{2}H) + 1,3-cyclohexadiene	**79**: 72/4/3	**21**: 13/7

Supplementary Discussion

Contrary to our quartet spin state complex $[Fe^{V}(O)(OH)(^{5tips3}tpa)]^{2+}$, the previously reported iron(V) complexes $Fe^{V}(O)(OAc)$ ($Fe^{V}(O)(OAc)$ ↔ $Fe^{IV}(O)(OAc^{\bullet})$ ↔ $Fe^{III}(OOAc)$[2,3] were characterized as the doublet spin state complexes. In order to check whether our theoretical approach reproduces the experimentally-observed trends, we have performed exploratory DFT calculations of relative energies of the $Fe^{V}(O)(OAc)$ isomers with the ^{5tips3}tpa ligand. First, we established that we can employ a smaller def2SVP basis set and obtain relative energies that agree within 2 kcal mol^{-1} with the results calculated using the def2TZVP basis set (Table 3). These calculations indicate that the doublet states of the isomers of **3** lie more than 10 kcal mol^{-1} above the respective quartet states for $[FeV(O)(OH)(^{5tips3}tpa)]^{2+}$ complexes.

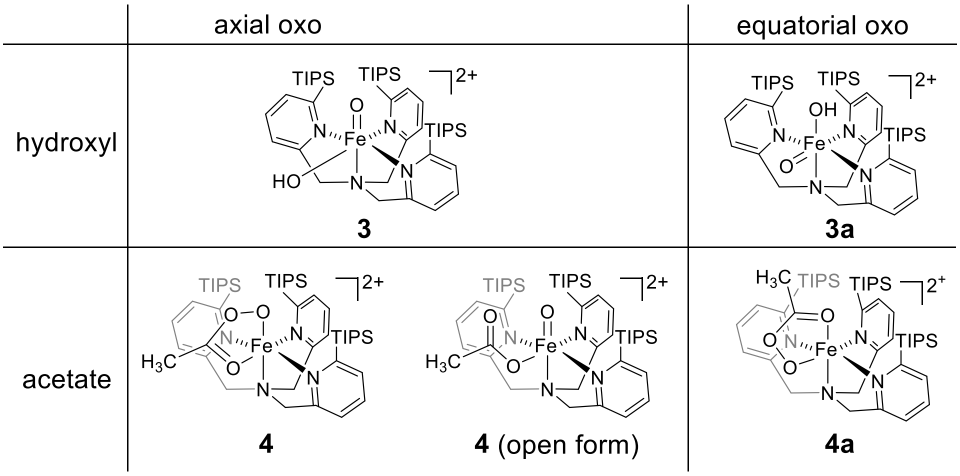

Fig. 8 Isomers of $[Fe^{V}(O)(OH)(^{5tips3}tpa)]^{2+}$ (**3**) and $[Fe(O)(OAc)(^{5tips3}tpa)]^{2+}$ (**4** open form) or $[Fe(OOAc)(^{5tips3}tpa)]^{2+}$ (**4** and **4a**).

Table 3. Relative energies (electronic energies E_{tot} and energies at 0 K E^{0K}) of isomers of **3** and **4**.[a]

	ΔE_{tot} [ΔE^{0K}] (kcal mol^{-1})	
Isomer[b]	B3LYP-D3/def2SVP	B3LYP-D3/def2TZVP
**^{4}3**	0.00 [0.00]	0.00 [0.00]
**^{2}3**	12.58 [11.99]	12.64 [12.29]
**^{4}3a**	0.57 [0.54]	0.50 [0.88]
**^{2}3a**	11.42 [10.85]	12.59 [12.66]
**^{6}4 (closed form)**	9.34 [7.47]	
**^{4}4 (open form)**	15.22 [14.06]	
**^{4}4 (closed form)**	12.44 [12.61]	
**^{2}4**	0.00 [0.00]	
**^{6}4a**	13.36 [11.53]	
**^{4}4a**	12.29 [11.01]	
**^{2}4a**	0.32 [0.66]	

[a]Please note that present calculations were exploratory in nature. We observed conformations resulting from the different orientation of the tips groups that varied in energy up to 2 kcal mol^{-1}. We cannot rule out that we missed some lower-lying conformers; however; the conformational variations do not change the clear preference of doublet electronic states for complexes **4/4a**.

[b]See Fig. 8 for the schematic depiction of each isomer.

[c] The initial $[Fe(O)(OAc)(^{5tips3}tpa)]^{2+}$ geometry converged to the closed form $[Fe(OOAc)(^{5tips3}tpa)]^{2+}$

The results obtained for different isomers of the peroxoacetate complex **4** indicate that, similarly to the reported carboxylate complexes, the ground state of **4** is doublet in both the axial (**4**) and the equatorial (**4a**) stereoisomers (Table 3). Almost all of our optimized structures correspond to the closed form, where peroxyacetate is coordinated to the iron

centre as a bidentate ligand. We also attempted to find the open forms, but these calculations converged to the closed geometry or ended up high in energy. We found that the quartet and sextet states of all isomers of **4** are more than 7 kcal mol^{-1} higher in energy than the doublet states. Interestingly, the most stable open-form complex that we optimized is also in the quartet state (^{4}**4**). This indicates, that the differences in spin state between the hydroxo and acetate complexes might indeed be caused by the ability of the iron(IV)-oxo acetate complexes to form the closed isomer.

XYZ coordinates of structures calculated at B3LYP-D3/def2TZVP[4-9] level.

Optimized Structures
The format of individual records is following:

number_of_atoms
NAME_method_multiplicityLetter (d = doublet, k = quartet, x = sextet) basis set charge multiplicity electronic_energy(Hartree) zero_point_energy(Hartree) number_of_imaginary_frequencies
atom1 x y z
...

134
4-3-2+ 2 4 -4264.7561668 1.1735945 0 B3LYP-D3/def2TZVP
Fe -0.1582904735 -1.4794416329 -0.9772937169
N -0.0588943919 0.4299510072 -1.6509915831
Si 0.3505286741 4.3196912702 -0.126749959
C 0.0316133174 1.4952859559 -0.8308906607
H -0.0679146956 1.2642145743 0.2193546801
Si -5.6047456785 -0.6190716672 0.8824104942
N -0.1803469945 -1.8939393645 -3.0416915771
C 0.2514087637 2.7888181607 -1.288121074
Si 5.3689204633 -1.0448852832 0.85012907
N -2.1225523486 -1.4505460695 -1.2479554304
C 0.3963847133 2.9293443721 -2.6757886674
H 0.6022555314 3.9016197918 -3.1044527852
N 1.8081546704 -1.5598014241 -1.2622170042
C 0.281273654 1.8379996915 -3.5229569425
H 0.3747861431 1.9490690694 -4.5956301979
C 0.042462949 0.5882339962 -2.9769999568
C 1.8717688595 5.293359517 -0.7041924802
H 1.6006306438 5.6284311057 -1.7137221556
C 3.1669212612 4.4776322617 -0.8266984919
H 3.9520979605 5.0770951357 -1.2938394714
H 3.0407380428 3.5761358757 -1.4318215938
H 3.5375478129 4.1683821776 0.1497932542
C 2.1033055424 6.5594213014 0.1404893766
H 2.8845254142 7.1777601817 -0.307502484
H 2.4323957376 6.3078266371 1.150719825
H 1.20759164 7.1774034498 0.2258554352
C 0.3744369496 3.6578510196 1.6512441939
H -0.4556759311 2.9390706244 1.6905769484
C 0.0677669916 4.7433895588 2.6985809872
H 0.0145216012 4.3038329541 3.6976628776
H -0.8814074847 5.2472111868 2.5130784827
H 0.8468947552 5.5065562629 2.7234048195
C 1.6643323096 2.9094989803 2.0206145646
H 1.5503480085 2.3846414505 2.9725883973
H 2.4936141628 3.6078944306 2.136632409
H 1.9667845031 2.1759792742 1.2691757048
C -1.2276033357 5.3290362666 -0.4287635435
H -1.1327271134 6.1856876007 0.2493606206
C -1.3620995221 5.8819180893 -1.8549990081
H -2.2410438007 6.5263499681 -1.9320903125
H -1.4982739833 5.078736865 -2.5853434923
H -0.5002403498 6.4766790626 -2.1610435485
C -2.4896872285 4.5536695948 -0.0290294562
H -3.3798989998 5.1787175743 -0.1353361482
H -2.4564092557 4.2085877201 1.00441684
H -2.6367655005 3.6765506364 -0.666173228
C -0.1680379311 -0.6268177757 -3.831895432
H -1.1270525486 -0.5239662326 -4.3425744987
H 0.5945303797 -0.6810679868 -4.6097328655
C -1.4476506887 -2.6471030518 -3.2401168829
H -1.2899215276 -3.6628174488 -2.8783825581
H -1.7248021618 -2.6977153524 -4.2945859655
C -2.5280971405 -2.0216018819 -2.4027455225
C -3.8753277521 -2.10054243 -2.6937783252
H -4.1988178752 -2.5400431163 -3.6282932065
C -4.7974547711 -1.6471904516 -1.7554168744
H -5.8515936157 -1.7371932474 -1.9822601798
C -4.3873835714 -1.1125714713 -0.5302974367
C -3.0095557819 -1.0157343206 -0.3427385338
H -2.5760111107 -0.6106912277 0.5607150108
C -4.8213018739 -1.3088944219 2.4681716817
H -3.9009410263 -0.7222332861 2.5892436674
C -4.4126322606 -2.789480569 2.4136171909
H -3.8816957417 -3.0701469165 3.326850712
H -3.7537742124 -3.0133901604 1.5705040031
H -5.2796776428 -3.4449307002 2.3320019727
C -5.6988404039 -1.0306834406 3.7016308953
H -5.1685935979 -1.3017365651 4.6176296248
H -6.6162689196 -1.6213466124 3.6745777873
H -5.9825442875 0.0202424893 3.7865327462
C -7.3011548986 -1.307809475 0.3934088922
H -7.4452981266 -0.9792632867 -0.6447478418
C -8.4497254713 -0.6883714103 1.2106833013
H -9.4109218005 -1.0641222293 0.8524837121
H -8.4772954531 0.3994249106 1.1339927439
H -8.3766234092 -0.9464369209 2.2683029058
C -7.3859610077 -2.84251714 0.4191421802
H -8.3214910414 -3.1822561598 -0.0310738063
H -7.3693029882 -3.2171523317 1.4435864673
H -6.5664422885 -3.3272350804 -0.1175782361
C -5.6549216576 1.2748783346 0.9382139973
H -6.4317225334 1.5092114937 1.6752684252
C -6.0866225867 1.8779518124 -0.407216534
H -6.1828911631 2.9638164199 -0.332648999
H -7.0480479178 1.4895525013 -0.7472996518
H -5.3490978805 1.6715398188 -1.1888900584
C -4.3498244675 1.9137774473 1.4295110403
H -4.4583834629 2.9983199915 1.4924451898
H -3.5228506457 1.7211957811 0.7385611307
H -4.0550273818 1.5587960763 2.4183279242
C 1.0472427405 -2.7063983192 -3.2544293109
H 1.3184122323 -2.7526383515 -4.3106281525
H 0.8349083157 -3.719048234 -2.9123142585
C 2.1679878077 -2.1627640351 -2.413352908
C 3.50544822 -2.3487094178 -2.7039131158
H 3.7934601139 -2.8219088548 -3.6335524944
C 4.4587987062 -1.9578257835 -1.77071499
H 5.5023441556 -2.1389323305 -1.9916406381
C 4.093280851 -1.3577460709 -0.5599253775
C 2.7271774985 -1.1603511077 -0.3711559087
H 2.3249473337 -0.6975066748 0.5186834529
C 7.0722583266 -0.9229259661 0.0292653184
H 7.2110900838 -1.906441707 -0.4384815289
C 7.2126276169 0.1356025757 -1.0773425388
H 8.1577227424 0.0030999195 -1.609424954
H 7.2157384281 1.1454144886 -0.6678751044
H 6.4118535209 0.0908021713 -1.819467742
C 8.1935365745 -0.7673870335 1.0732586639
H 9.1722795066 -0.8464808318 0.5948593527
H 8.1486125721 -1.5299787444 1.8528619649
H 8.150711461 0.2095666036 1.558463145
C 5.2482910849 -2.611752751 1.9170723554
H 5.9413663077 -2.4343744033 2.7479817869
C 5.7108792173 -3.8792781202 1.184193502
H 5.6444673514 -4.7492929214 1.8419752637
H 6.7451700646 -3.8102567586 0.8447100703
H 5.0850624598 -4.0907472863 0.3113873648
C 3.8470692505 -2.8112074144 2.5158029669
H 3.8407783618 -3.6603245548 3.2032863166
H 3.1105258354 -3.0296339831 1.7359570087
H 3.4969568837 -1.9397855987 3.0728060437
C 4.7937858622 0.4704430889 1.833890541
H 3.7101437367 0.3445635535 1.9632062673
C 5.0239190108 1.795851758 1.0947032111
H 4.5773602663 2.6223220381 1.6497651701
H 4.5979893151 1.80216216 0.0886103108
H 6.08818066 2.0143017577 1.0036165103
C 5.4034905092 0.5305399203 3.2462231499
H 4.9957953233 1.3820144307 3.7967823726
H 6.4866495007 0.6589106729 3.2117785609
H 5.1950809888 -0.3664793586 3.8301622858
O -0.2007044012 -3.2494692809 -0.71714289
O -0.143970133 -1.0254257103 0.5875872572
H -0.2005990069 -3.4797441212 0.2228988937

134
2-3-2+ 2 2 -4264.7360293 1.1730293 0 B3LYP-D3/def2TZVP
Fe -0.158843186 -1.7343335406 -0.6681211815

N -0.0710474856 0.1160301985 -1.4632444305
Si 0.2911728597 4.1171721354 -0.2377873899
C 0.0063485134 1.2426094122 -0.7266585812
H -0.0956881209 1.0930369268 0.3381364689
Si -5.6196240336 -0.7752108019 1.1001165592
N -0.1682057904 -2.3033788097 -2.6804220399
C 0.2149242833 2.5005313251 -1.2777956665
Si 5.3602506823 -1.0958396206 1.1322376596
N -2.1182543871 -1.7355524403 -0.9458051125
C 0.3640691177 2.5391658376 -2.6717205885
H 0.5620579232 3.4786969325 -3.1711706602
N 1.8074108566 -1.8122316264 -0.9365860656
C 0.2622907213 1.3864080476 -3.4345890018
H 0.358545583 1.4177404689 -4.5122864914
C 0.0330588045 0.1787766302 -2.7972802903
C 1.8031700901 5.0629693071 -0.8820758128
H 1.5318650601 5.3184093718 -1.9145527816
C 3.1082886195 4.2561569884 -0.9396260243
H 3.8879563477 4.8282497263 -1.4484556172
H 2.9948654165 3.3102575352 -1.4755080265
H 3.4790644439 4.0258005405 0.0583290665
C 2.0166718569 6.3912508192 -0.1337280011
H 2.7920705029 6.9838729122 -0.6245395105
H 2.3451822839 6.2196694371 0.8932963899
H 1.1134373145 7.0030381574 -0.0969707386
C 0.3143669227 3.5943963737 1.5860956118
H -0.5083897231 2.871807535 1.6773308129
C -0.0079031168 4.7530359759 2.5466957444
H -0.06188864 4.3901136819 3.5760650486
H -0.9609248463 5.2317991706 2.3193928787
H 0.7636873463 5.5236307251 2.5168224386
C 1.6106063646 2.890355744 2.0156910279
H 1.4984845847 2.438479757 3.0046498523
H 2.4320058135 3.6045127886 2.0808702321
H 1.9241542135 2.105149605 1.3233213987
C -1.2964866334 5.0826030659 -0.6225495753
H -1.2131442951 5.9904481012 -0.0129029873
C -1.4315447334 5.5215757306 -2.0878062043
H -2.3164202436 6.1493433001 -2.2169454181
H -1.5574539315 4.6627311675 -2.7537854116
H -0.5745111729 6.0992829423 -2.4370059835
C -2.55196986 4.3275377639 -0.1675036848
H -3.4481837743 4.9329832623 -0.3247031219
H -2.5190401036 4.0642707428 0.8897143447
H -2.6877134072 3.4020682532 -0.7349394622
C -0.163643638 -1.0969938318 -3.5616931322
H -1.1215440387 -1.0400881694 -4.0815077809
H 0.6019876041 -1.2014519609 -4.3313167665
C -1.4274500933 -3.0798627169 -2.8321139749
H -1.2657396795 -4.0644754327 -2.3936916724
H -1.6953450659 -3.2132851585 -3.8817708598
C -2.5170488655 -2.3985159118 -2.0524204451
C -3.8620105227 -2.5096815576 -2.343952834
H -4.1772728179 -3.0237854906 -3.2424939234
C -4.7923262037 -1.9901369558 -1.4493904739
H -5.8445840968 -2.1047074977 -1.6736153215
C -4.3914308602 -1.3579805526 -0.2683550319
C -3.0154501371 -1.2353628522 -0.0844021465
H -2.5920614659 -0.7545058897 0.7861080803
C -4.8392531894 -1.347273804 2.7339086694
H -3.9243303498 -0.7458042544 2.8182154525
C -4.4182063734 -2.8244717343 2.7846117662
H -3.8897376545 -3.0364307168 3.7176430083
H -3.753425246 -3.1008761345 1.961916442
H -5.2793533571 -3.4914422666 2.7449214879
C -5.7257268824 -0.9914577117 3.9407119961
H -5.1986130761 -1.1938009065 4.8760838851
H -6.6383135027 -1.5901239547 3.9500686837
H -6.0182651194 0.0604120905 3.9508729187
C -7.3085364751 -1.5101250183 0.6537927094
H -7.449190373 -1.258302167 -0.4060601345
C -8.4663949064 -0.842578697 1.4183629292
H -9.4225974755 -1.2514685157 1.0838855558
H -8.5023456812 0.2364573835 1.2625247611
H -8.3969312951 -1.022395806 2.492325513
C -7.3824292484 -3.0396120734 0.7896677713
H -8.3127486523 -3.4180703612 0.3600018868
H -7.3693241443 -3.3392287728 1.8385744505
H -6.5562682073 -3.5555919764 0.293867937
C -5.686141385 1.1175199916 1.0264199524
H -6.4736372641 1.394089673 1.7369642007
C -6.107418076 1.6231448738 -0.3618075778
H -6.2146835794 2.7105355623 -0.3626962228
H -7.0611740373 1.2027362512 -0.6852288831
H -5.3588818391 1.3712034152 -1.1192424749
C -4.3928114205 1.8006557745 1.4880555239
H -4.5111821351 2.885913487 1.4748466318
H -3.5555328328 1.56867207 0.8221716042
H -4.1079533214 1.5172870745 2.5025456319
C 1.0668678469 -3.1172429915 -2.8333707838
H 1.3382684756 -3.2414980001 -3.8832171158
H 0.8653100457 -4.1036093066 -2.4156554685
C 2.1798647878 -2.4970386451 -2.0364151639
C 3.5199486341 -2.6874827714 -2.3123429855
H 3.8150606829 -3.2269960591 -3.202775493
C 4.4670585941 -2.2130489071 -1.4125195257
H 5.5132489537 -2.3959650426 -1.6188256732
C 4.0910577529 -1.5267125249 -0.2516751136
C 2.7224289554 -1.3324036428 -0.0807859087
H 2.3156680974 -0.8052849356 0.7704309677
C 7.063921241 -1.0129315491 0.3073258879
H 7.2156900241 -2.0261407197 -0.0872634643
C 7.1938170209 -0.0364611999 -0.8735275303
H 8.1418576845 -0.1955489787 -1.39293852
H 7.1833167454 1.0005559714 -0.5390542864
H 6.3955451633 -0.145701322 -1.61160952
C 8.1808866099 -0.7679552292 1.3386225703
H 9.161581366 -0.8696280443 0.8685634029
H 8.1435388894 -1.4722107556 2.1716882294
H 8.1251533674 0.2411423353 1.7513051538
C 5.2577949992 -2.5826583251 2.3104482705
H 5.9456417786 -2.3357685906 3.1277832116
C 5.7397883576 -3.8937991432 1.6730391696
H 5.6832671333 -4.7144358232 2.3923504739
H 6.7740627772 -3.8353631583 1.3316092711
H 5.1198093394 -4.1768952174 0.8164999071
C 3.8575853046 -2.7573503573 2.9191378279
H 3.8602916149 -3.5545074035 3.6662912253
H 3.126756376 -3.0415849235 2.1551249251
H 3.4940063965 -1.8526698423 3.4106948435
C 4.7660136315 0.4790979271 2.0048651858
H 3.6834092962 0.3511726691 2.1408333349
C 4.9840435842 1.7507051249 1.1733686949
H 4.5278590202 2.6099563386 1.6674550327
H 4.5602746168 1.6806864344 0.1688378551
H 6.0461979779 1.9731301924 1.0684395273
C 5.3713280898 0.6466845933 3.4104150461
H 4.9537924163 1.5314425671 3.8974640571
H 6.4532387574 0.7831449594 3.3689589826
H 5.1703024722 -0.2079876319 4.0570763031
O -0.1866281716 -3.476966089 -0.260041327
O -0.1528154425 -1.167978215 0.8717073703
H -0.1581526334 -3.6431446884 0.6940356574

134
4-3a-2+ 2 4 -4264.7553734 1.1742051 0 B3LYP-D3/def2TZVP
Fe 0.0788947301 -2.0298486188 -0.3756249069
N 0.0041873449 -0.1967558398 -1.3168851503
Si -0.1931299615 3.8749512481 -0.3737403007
C -0.0451199599 0.983613397 -0.6766539295
H -0.0695283063 0.9071817023 0.3998082437
Si -5.5355472579 -0.771954996 0.8278232708
N 0.1132636881 -2.7293099404 -2.3220776706
C -0.0581794834 2.2110318944 -1.3312665601
Si 5.5229342744 -0.5068978019 1.0940979397
N -1.8860141709 -2.1480463027 -0.7021807571
C -0.0114158306 2.1505764662 -2.7306259848
H -0.007886665 3.0612726642 -3.3154694146
N 2.0536349724 -1.9848500547 -0.6780752141
C 0.029631595 0.9330171777 -3.3963607782
H 0.0581109001 0.8895996634 -4.4776618703
C 0.0327866484 -0.2354995305 -2.6522075562
C 0.8411849554 5.1319936189 -1.3411685434
H 0.4129972372 5.1051836205 -2.3520250731
C 2.3302262146 4.7755621721 -1.464401143
H 2.8275408897 5.4476509045 -2.1677675034
H 2.4947585138 3.7536194638 -1.8142608561
H 2.8411880034 4.8822633699 -0.5063718766
C 0.6647242465 6.5706726468 -0.8248510824
H 1.17959423 7.2748246818 -1.482523936
H 1.0901336073 6.6931114811 0.1727117133
H -0.3825325878 6.8738874272 -0.7800150473
C 0.3088976796 3.5136201572 1.4203107689
H -0.3749587946 2.7099610869 1.725668367
C 0.0415456296 4.7028719979 2.359919664
H 0.2141461343 4.4147301836 3.3998298677
H -0.9828779407 5.0715787776 2.2889612752
H 0.7087257203 5.5384286689 2.1427788093
C 1.7398800339 2.9917092619 1.6045793175
H 1.8875208128 2.6225307213 2.623142248
H 2.4738117836 3.7813103403 1.4419427903
H 1.9852985254 2.1806065242 0.9167168235
C -2.0282987158 4.3542295441 -0.4191578128
H -2.0792231526 5.3300514205 0.0773090595
C -2.5721021895 4.5286457663 -1.8453236578
H -3.609067656 4.8723055078 -1.8228078477
H -2.5621350955 3.5830169328 -2.3954656233
H -2.0027289304 5.2593842426 -2.422265101
C -2.9004221507 3.3764499656 0.3784287798
H -3.9480837487 3.6853457511 0.3545775551
H -2.6087480306 3.3102079092 1.427413741
H -2.8495815725 2.3702440262 -0.0484805288
C 0.0465923639 -1.5931922991 -3.3044177728
H -0.8551102527 -1.7083749757 -3.9071335087
H 0.8935247847 -1.6653619618 -3.9873765309
C -1.0810489331 -3.6230614295 -2.4121182682
H -0.8399346392 -4.5444104024 -1.8816410004
H -1.3063774941 -3.8727932043 -3.4500952655
C -2.2305007977 -2.958324311 -1.7204460627
C -3.561596171 -3.1756632066 -2.0178171555
H -3.8321913244 -3.8300974839 -2.8360342841
C -4.5303146182 -2.5531197478 -1.2402181059
H -5.5720497231 -2.7507854798 -1.4545085706
C -4.1822326872 -1.6778529964 -0.204581942
C -2.8173481328 -1.5099000606 0.0201223529
H -2.42863799 -0.8668268532 0.7954710203
C -4.6196076659 0.3841289119 2.0266201384
H -3.8288195818 0.8418235249 1.4188965011
C -3.9455811876 -0.3272581099 3.2130830546
H -3.2992067293 0.3665914075 3.7571206927
H -3.3372004712 -1.1861526162 2.9181486283
H -4.6887896764 -0.6931129702 3.9227411033
C -5.5037677147 1.5367706651 2.5359094811
H -4.9272543497 2.1965189455 3.1892816844
H -6.350421132 1.1673508196 3.1169824817
H -5.8997345703 2.1478667252 1.7245751694
C -6.5816586732 -2.1442426504 1.6078186969
H -7.0594283164 -2.6348021234 0.7498578003
C -7.7027653939 -1.5608737811 2.4859110107
H -8.3720027327 -2.3536522603 2.8277811114
H -8.3122144349 -0.8266529141 1.9550166272
H -7.2966990422 -1.0756311808 3.3760562384
C -5.7899655069 -3.2220645408 2.3630858198
H -6.4547758067 -4.0305124514 2.6761816253
H -5.3225351786 -2.8240922422 3.2639906171
H -5.0031711773 -3.666632815 1.7489115214
C -6.5688760115 0.2553884855 -0.394629979
H -7.200941508 0.8651043804 0.2620647981
C -7.5078369783 -0.5512628738 -1.3029139949
H -8.1517373615 0.1219922482 -1.8737376642
H -8.1595088853 -1.2256437413 -0.7461752592
H -6.9527110129 -1.1427157583 -2.0356695685
C -5.702747647 1.2122270751 -1.2267001766
H -6.3268009221 1.8559773502 -1.8513903296
H -5.0362993836 0.6611308491 -1.8976164114
H -5.0856988558 1.8607084429 -0.6058798995
C 1.3920195081 -3.4932155229 -2.4234074256
H 1.6420443139 -3.7025846225 -3.4645816713

H 1.2452567021 -4.4434838698 -1.9093048711
C 2.4718991725 -2.7358776672 -1.7159598384
C 3.8168548538 -2.8320104844 -2.0092971784
H 4.144292008 -3.427918915 -2.8511479009
C 4.7300335636 -2.1745978888 -1.1918287037
H 5.7849544741 -2.2763900584 -1.4084415411
C 4.3079381964 -1.3932144558 -0.1126694951
C 2.9281303898 -1.316990652 0.0852051837
H 2.4852003813 -0.7247399281 0.872896555
C 7.2740257845 -0.7719022565 0.4219860994
H 7.3286861412 -1.8452920618 0.1966541958
C 7.5833596032 -0.0005020445 -0.8721090585
H 8.5397269243 -0.3233253135 -1.2899276496
H 7.6649766495 1.0698531053 -0.6768634624
H 6.824173119 -0.1314694161 -1.6472731272
C 8.3579536575 -0.48986755 1.4800711873
H 9.3460765253 -0.7178261996 1.0739452575
H 8.2310663845 -1.0904038607 2.3811798453
H 8.3665809296 0.559705873 1.7780807119
C 5.33396704 -1.3855297088 2.7664812457
H 6.0553804207 -0.8776112212 3.4177163713
C 5.7376556235 -2.8663562918 2.6807696537
H 5.7013098512 -3.334799008 3.6671255249
H 6.7485629065 -3.0038256663 2.2948112424
H 5.0560565444 -3.4260152147 2.0331819448
C 3.9506043703 -1.2417832153 3.417509222
H 3.9574765306 -1.6658589491 4.4243741405
H 3.1869945724 -1.7882781965 2.855083803
H 3.6310160822 -0.2015958038 3.5058825885
C 4.9226065619 1.2882030222 1.1808901565
H 3.9505168424 1.2241938765 1.6849382387
C 4.6862832909 1.9464440596 -0.1871672355
H 4.2771080664 2.9499531763 -0.061609612
H 3.9811382196 1.3833779245 -0.804585542
H 5.6108293691 2.046766732 -0.7556607296
C 5.8287240688 2.1662723186 2.0607658667
H 5.3725659342 3.1466653522 2.2193074664
H 6.7976757919 2.3353984856 1.5876994083
H 6.0108455648 1.7281169225 3.043969155
O 0.1428827262 -3.554106983 0.2268853141
O 0.0356789779 -1.1858383729 1.1814931605
H 0.0594859496 -1.8022180502 1.9291903712

134
2-3a-2+ 2 2 -4264.7361169 1.1737163 0 B3LYP-D3/def2TZVP
Fe 0.0754469699 -2.0293641287 -0.3777809435
N -0.0025566547 -0.2043299893 -1.3059134604
Si -0.190883151 3.8703866891 -0.3681203645
C -0.0482683489 0.9777001367 -0.66772633
H -0.0681274051 0.9041081264 0.4088802351
Si -5.5338430517 -0.7620471042 0.8338045045
N 0.1032152962 -2.7360583673 -2.3101662302
C -0.063240652 2.2046537066 -1.3232000412
Si 5.5226740105 -0.5086059435 1.0777777597
N -1.8857981232 -2.1515629728 -0.6926177497
C -0.0227597098 2.1432408855 -2.7226613323
H -0.0211117869 3.053355344 -3.3083835749
N 2.0447642375 -1.9866377191 -0.6820822155
C 0.0144584199 0.9249666158 -3.3869631664
H 0.0381864016 0.8803379286 -4.468324907
C 0.0200850962 -0.2428384633 -2.6417430604
C 0.8408181906 5.1242209189 -1.342707466
H 0.4088268001 5.0947445334 -2.3518743221
C 2.3290617134 4.7662829818 -1.4705633972
H 2.8242572762 5.4357592751 -2.1779121065
H 2.4914516241 3.7431175564 -1.8177612918
H 2.8436689525 4.8756894181 -0.5147971357
C 0.6676346129 6.5647549488 -0.8304823376
H 1.1807715538 7.2661818805 -1.4924255045
H 1.0968416538 6.690289983 0.1650883263
H -0.3791368723 6.8691687972 -0.7828979033
C 0.3190925442 3.5118945499 1.4243363339
H -0.3655123601 2.7108523984 1.7349091109
C 0.0597341262 4.7041249837 2.3623332338
H 0.2367588891 4.4182450856 3.4021358309
H -0.9640043494 5.0754819648 2.2955073056
H 0.7280655781 5.537279704 2.1396168657
C 1.7494083803 2.9863420511 1.6028018581
H 1.9010064208 2.6187789859 2.621401119
H 2.4848614689 3.7734091964 1.4350089742
H 1.9891849484 2.1733230504 0.9152031211
C -2.0249286692 4.3545722276 -0.4051006643
H -2.0704640354 5.3329439079 0.0868285803
C -2.5765251748 4.5233639929 -1.8289842852
H -3.6121670001 4.8706997996 -1.8022111182
H -2.5729327659 3.5748574418 -2.3741861829
H -2.0080964517 5.2491257766 -2.4131175832
C -2.8947951035 3.3828548642 0.4021180996
H -3.9422990322 3.6925222828 0.3808181821
H -2.5988318756 3.3227004917 1.4502619112
H -2.846513032 2.3740978117 -0.018898203
C 0.029323758 -1.6004712561 -3.2934688571
H -0.8768186143 -1.7157977778 -3.8894719058
H 0.8707304355 -1.6728959966 -3.9831934485
C -1.0891879498 -3.6315363243 -2.3995530376
H -0.8463912028 -4.5541846872 -1.8715643834
H -1.3176904892 -3.881228738 -3.4368693535
C -2.2364866916 -2.9663702719 -1.7044829405
C -3.5682529668 -3.1860941004 -1.9977733632
H -3.8407113192 -3.8454506529 -2.8113722232
C -4.5349225284 -2.5582919348 -1.2224456412
H -5.5773487351 -2.7562232842 -1.4330627889
C -4.1826801021 -1.6764570462 -0.1936352216
C -2.8178434722 -1.5080712963 0.0266370661
H -2.4287294021 -0.8590847877 0.796647409
C -4.6165767372 0.3917087418 2.0336983207
H -3.8235277209 0.8467049991 1.4270669358
C -3.9463213138 -0.3214213029 3.2211654495
H -3.2979611925 0.3702936075 3.7655791254
H -3.3408617159 -1.1826786869 2.9268477722
H -4.6914244069 -0.6843900772 3.9303197169
C -5.4978786753 1.5473892336 2.5409829857
H -4.9202682919 2.205936179 3.1945800162
H -6.3465790777 1.1811492964 3.1210420451
H -5.8907132174 2.1589800291 1.7284813233
C -6.5887474385 -2.127721084 1.613976424
H -7.0679931705 -2.6164791255 0.7558837608
C -7.7077750343 -1.5375953018 2.4900675519
H -8.3816978046 -2.3264900656 2.8317502321
H -8.3126481888 -0.800620863 1.9577364509
H -7.3002574773 -1.0538127368 3.3803136595
C -5.8036465786 -3.208850309 2.371273151
H -6.4731963713 -4.012879622 2.6856604129
H -5.3340631176 -2.811950919 3.2715361726
H -5.0193771424 -3.6590625093 1.7579531017
C -6.5586077918 0.2678908675 -0.3936999613
H -7.1912612249 0.8802378328 0.2600283403
C -7.4960801925 -0.5369713574 -1.3051147485
H -8.1360233499 0.1375881836 -1.8788515492
H -8.1517362881 -1.2094339999 -0.750663695
H -6.9393442497 -1.1301798126 -2.0352806739
C -5.6862441822 1.2212458654 -1.2232360189
H -6.3059896154 1.8661437742 -1.8510555625
H -5.0187837288 0.6675755792 -1.8909973877
H -5.06990814 1.8686726698 -0.6006839825
C 1.3818052657 -3.4973186385 -2.4227052243
H 1.627454305 -3.7031199706 -3.4656569446
H 1.2406315238 -4.4507619563 -1.9123420492
C 2.4631082983 -2.73906857 -1.7182042595
C 3.8070622269 -2.8362563983 -2.0165347127
H 4.1315316057 -3.4342817711 -2.8579995036
C 4.7228882771 -2.1768600205 -1.2042368079
H 5.7771263006 -2.2788646751 -1.4238733566
C 4.3029757297 -1.3928727129 -0.1259181136
C 2.9243641825 -1.3157176927 0.075155197
H 2.4855902804 -0.7194010261 0.8618986851
C 7.2719529447 -0.7781342985 0.4022861599
H 7.3234658406 -1.8516381496 0.1767925323
C 7.5803918541 -0.0073117187 -0.8923254991
H 8.5351954864 -0.3322382643 -1.3121118099
H 7.6648310515 1.0628369644 -0.6971570262
H 6.8193417206 -0.13652226 -1.6659174016
C 8.3586587435 -0.49886197 1.4582185122
H 9.3453381581 -0.7295725563 1.0500980256
H 8.2320322264 -1.098930677 2.3596821376
H 8.370790976 0.5507066143 1.7560517844
C 5.3357650419 -1.3872761619 2.7506380297
H 6.0596364926 -0.8811329892 3.4004822825
C 5.7360060679 -2.8689293315 2.6636533038
H 5.7007836846 -3.3377607538 3.64987743
H 6.7457638922 -3.0083844372 2.2754528072
H 5.0519539652 -3.4268191446 2.0170887349
C 3.9542522445 -1.240730384 3.4048413638
H 3.9621273257 -1.6654100866 4.4114581779
H 3.1883358464 -1.7851776753 2.8434135151
H 3.6372320751 -0.1998669391 3.4942926355
C 4.9283431766 1.2881929435 1.1672880625
H 3.9578080205 1.2273820896 1.6746140922
C 4.6893974529 1.9472487755 -0.1999054503
H 4.2843436179 2.9522112018 -0.0728475009
H 3.9799223721 1.3866881146 -0.8145727808
H 5.6121777132 2.0442578265 -0.7718423482
C 5.8402797161 2.1632298137 2.0441630656
H 5.3875502433 3.1448861108 2.2047120656
H 6.8079312121 2.3297486249 1.5675594736
H 6.0248003066 1.7242381007 3.0265563075
O 0.1435093743 -3.5607859548 0.2392137259
O 0.0373040767 -1.1951096415 1.1883235813
H 0.0659294767 -1.8082037998 1.9394497465

134
6-2a-2+ 2 6 -4264.741321 1.1706268 0 B3LYP-D3/def2TZVP
Fe 0.4151338366 -0.3751048734 -0.5851978258
N 0.3216902361 1.4479199425 -1.5067795435
Si -0.2873204368 5.4283971483 -0.3666581686
C 0.1454941284 2.5895833542 -0.8117121421
H 0.1127022447 2.4618260373 0.2620137769
Si -3.3302227511 -3.7000917647 2.3972469135
N 0.1399614388 -0.9667217608 -2.7482062189
C 0.0203823373 3.8389773358 -1.4073237089
Si 5.4845764038 -2.6090715295 1.2236671631
N -1.2871217199 -1.5312817119 -0.5093379025
C 0.0782861236 3.8471490607 -2.8068154056
H -0.0157315831 4.7804790762 -3.3472231115
N 2.0554274577 -1.572612046 -0.8981296979
C 0.2562137313 2.6734322892 -3.532763368
H 0.299384826 2.6890174872 -4.6140098339
C 0.3855375693 1.4774077206 -2.8537867128
C 0.5040026193 6.8494258128 -1.3345013416
H -0.0102822114 6.8266313371 -2.3044890817
C 2.0072050238 6.684788871 -1.6073500327
H 2.3566793183 7.4566259844 -2.2971416764
H 2.2530583973 5.7155931038 -2.0488787036
H 2.590918126 6.7852993511 -0.6918528242
C 0.2061332994 8.2238669253 -0.7096552082
H 0.5517990807 9.0235822121 -1.3685587937
H 0.7217273635 8.3476372115 0.2440423788
H -0.8596476817 8.3823296723 -0.5353712465
C 0.3893772362 5.0654597745 1.3690045564
H -0.1511246166 4.1617249695 1.6834067414
C 0.0297823857 6.1653946164 2.3837278209
H 0.3242786624 5.8649898166 3.3923134765
H -1.0398940181 6.3800162708 2.4062646955
H 0.5505404197 7.0978030954 2.1617252603
C 1.8918967029 4.7463955876 1.4241264159
H 2.1700493457 4.3849577408 2.4177782735
H 2.4937122998 5.6346396506 1.2295229662
H 2.1930268203 3.9853009331 0.6994979177
C -2.1739268485 5.630902168 -0.3037884356
H -2.3426208657 6.5247171512 0.3082662302
C -2.7880343456 5.8909077606 -1.6878020958
H -3.8674805 6.0404769136 -1.6078057095
H -2.6336597649 5.0413238707 -2.3605573485
H -2.3745974539 6.7785282603 -2.1689643626
C -2.8735585289 4.4515978211 0.3884983503
H -3.9507387725 4.6247283677 0.4491063255
H -2.5131824153 4.2911668626 1.4062022395
H -2.7307729544 3.520898297 -0.1707277043

C 0.6708443747 0.1625741076 -3.5368507966
H 0.2826188261 0.1593636103 -4.5593033478
H 1.75454025 0.0375057416 -3.6040871377
C -1.3196818626 -1.149442015 -2.9014996182
H -1.5637329217 -1.7066252856 -3.8100452941
H -1.7677962126 -0.1575734741 -2.9987595182
C -1.8899687144 -1.827045005 -1.6794132453
C -2.9744122256 -2.6810808705 -1.7096932425
H -3.4533928352 -2.9155662898 -2.6514139589
C -3.4285322331 -3.2405046079 -0.5171354826
H -4.272733874 -3.9170451485 -0.5527312095
C -2.804317918 -2.9598763658 0.7026954931
C -1.7288117069 -2.0785475521 0.6361477502
H -1.1683424787 -1.796892656 1.5170993636
C -1.7064631252 -3.9916541028 3.3415248323
H -1.3053176809 -2.9818768195 3.49892767
C -0.6353855095 -4.79204353 2.5836058305
H 0.3000200224 -4.8082838848 3.149229805
H -0.4121470628 -4.3723110803 1.5991388402
H -0.9396992907 -5.8283104827 2.4339293941
C -1.9518796313 -4.5973752056 4.7344043905
H -1.0260676549 -4.6127155341 5.3149144373
H -2.3020674353 -5.6282001971 4.6615054215
H -2.6898918733 -4.0359076802 5.3103441808
C -4.3921708722 -5.2200245599 2.0219843013
H -5.1538571729 -4.8487023231 1.3229843169
C -5.1513167213 -5.7450693127 3.2543937154
H -5.8303221212 -6.5500785903 2.9638646673
H -5.7511903599 -4.9720174765 3.73677864
H -4.471003766 -6.1534972192 4.0033797283
C -3.6345430354 -6.3613160325 1.3242150558
H -4.330869719 -7.13050311 0.9820140547
H -2.9354015797 -6.8448196379 2.0081163614
H -3.0626937948 -6.0250073827 0.4553779941
C -4.35855017 -2.3576414165 3.258532879
H -4.6498077483 -2.7962197262 4.2202370239
C -5.6456549181 -2.0325852762 2.4836001023
H -6.2537589729 -1.3096389786 3.0325340775
H -6.264773127 -2.9150454247 2.3156629398
H -5.422828725 -1.5894896124 1.5081216697
C -3.5655563718 -1.0761534841 3.5558891398
H -4.1943566245 -0.3490040164 4.0756101396
H -3.2198050358 -0.5951939243 2.6352520124
H -2.6954335862 -1.2612893204 4.1885649613
C 0.9041141842 -2.2166173182 -2.9416839592
H 1.1709651964 -2.3658102994 -3.9912727446
H 0.2572852722 -3.049557434 -2.6560363559
C 2.1279370496 -2.2567319162 -2.058267426
C 3.2424732005 -3.0193821673 -2.3504311286
H 3.3020924285 -3.5518887387 -3.290798816
C 4.2691841825 -3.1054904136 -1.4139115462
H 5.134435044 -3.7125420345 -1.6480581253
C 4.1952396937 -2.4308143487 -0.190217848
C 3.0541868921 -1.6592443607 -0.0004315947
H 2.8997757933 -1.0851714821 0.9014275272
C 7.1104551031 -3.1468631426 0.4195910221
H 6.8341951235 -4.0241644735 -0.1807683024
C 7.7152805551 -2.1040886721 -0.5337889672
H 8.5520136828 -2.5336359759 -1.0895646306
H 8.1032638017 -1.2460127336 0.0165849911
H 6.9939581087 -1.7272635689 -1.2636571472
C 8.1559849077 -3.6258030474 1.4421403345
H 9.0335469211 -4.023702748 0.9274123283
H 7.7741244732 -4.4147832825 2.0920993057
H 8.4991033182 -2.8080551663 2.0776501326
C 4.7919615253 -3.9872329636 2.3335884172
H 5.5177789024 -4.0858306544 3.1492189944
C 4.7202864941 -5.3375136233 1.6032621413
H 4.366179497 -6.1218600976 2.276624555
H 5.6900189 -5.6555062148 1.2178289821
H 4.0219725475 -5.2985409501 0.7614281613
C 3.4329646798 -3.6394293227 2.9602408916
H 3.0977603036 -4.4471274039 3.616109626
H 2.6593048777 -3.5072009575 2.1976676569
H 3.4697969982 -2.7289388657 3.561146434
C 5.4710921768 -0.9544592064 2.1542277493
H 4.4433415776 -0.8736482047 2.5339520848
C 5.7365327584 0.2853840304 1.2858655658
H 5.5700243814 1.1991216178 1.8625471405
H 5.0910304487 0.328710547 0.4047563465
H 6.7676562099 0.3132804839 0.9331711788
C 6.3965222522 -0.96733083 3.3839542744
H 6.2525930412 -0.0636093474 3.9813389226
H 7.4470700189 -0.9922588202 3.0900229893
H 6.2137444531 -1.82339617 4.0362203826
O 0.844586813 -0.7608579725 2.217425688
O 0.6277143933 0.169078966 1.1780827698
H 0.8862290465 -0.184251739 2.998057363

134
2-2a-2+ 2 2 -4264.7282165 1.173269 0 B3LYP-D3/def2TZVP
Fe -0.3236804752 -0.7182223423 -0.5928997286
N 0.0471171371 1.074357082 -1.3789834408
Si 0.763300679 4.9787973867 -0.0740099948
C 0.2206562097 2.187888696 -0.6433406777
H 0.1546652384 2.0312784611 0.4227093116
Si -3.7265864607 -4.3997319984 2.3712145475
N -0.4868877611 -1.2568503467 -2.5882638646
C 0.4835969224 3.4374046631 -1.1893512155
Si 5.1312393416 -1.309625805 0.9558704343
N -1.7963657072 -2.0608001925 -0.4754662131
C 0.5204434335 3.4917920306 -2.5888986386
H 0.714373536 4.430258562 -3.0925404973
N 1.4018586009 -1.5784170382 -0.8276980979
C 0.3241464561 2.3513388127 -3.3588262563
H 0.3604628909 2.3956100084 -4.4396230529
C 0.111389472 1.1421084097 -2.722175908
C 2.161045564 5.9575709054 -0.8985277677
H 1.7582150071 6.2057301539 -1.8892799999
C 3.4593237835 5.1656374238 -1.119439227
H 4.1559512321 5.7379197423 -1.7367569531
H 3.2889541512 4.2082930873 -1.6181015354
H 3.9643440233 4.9594271778 -0.1749455282
C 2.4462491561 7.2907217679 -0.1855222429
H 3.1444150498 7.8942916539 -0.7700886746
H 2.9028929454 7.1280543184 0.7924587969
H 1.5443069486 7.8872038667 -0.0368385614
C 1.0902961343 4.3199505912 1.6795338474
H 0.2263878386 3.6740265975 1.8871106153
C 1.0674316834 5.4363371554 2.7387239811
H 1.1513129496 5.0125201694 3.7426629299
H 0.146873733 6.0207002813 2.7075683028
H 1.902158883 6.1266597547 2.6096965107
C 2.357338545 3.4626407962 1.8286838744
H 2.3837694168 2.9832458301 2.811355736
H 3.2567863232 4.0739452539 1.7493809959
H 2.434762286 2.6770438795 1.0737034811
C -0.8613744789 5.9571222278 -0.1042521992
H -0.6860004148 6.8218992703 0.5463645422
C -1.2133294332 6.4896704708 -1.5014714388
H -2.1299774707 7.0831267356 -1.4660685938
H -1.3920627282 5.6731172358 -2.2075273634
H -0.4311130808 7.1280391775 -1.9151113476
C -2.0331385823 5.1563903649 0.4852313095
H -2.9441496827 5.7593718225 0.4971189058
H -1.8439997523 4.8339755615 1.510579963
H -2.2461304136 4.2650426261 -0.1133210048
C 0.0594427834 -0.1706167985 -3.4533665967
H -0.5097164218 -0.0967375978 -4.3820099766
H 1.0805521244 -0.4412545279 -3.7277695101
C -1.9384911159 -1.4926244462 -2.8056576778
H -2.1217903679 -2.0073225734 -3.7521666158
H -2.4239833443 -0.514946118 -2.857135974
C -2.4682631348 -2.2681390404 -1.6309935362
C -3.5520181622 -3.1191886876 -1.6731042852
H -4.0788519625 -3.2810249561 -2.6043378219
C -3.9459608509 -3.7651536096 -0.5022730748
H -4.7950476872 -4.4353259438 -0.5411940352
C -3.2581409538 -3.5724168952 0.6987844883
C -2.1781404653 -2.6931597048 0.6456602452
H -1.5737332284 -2.4713281217 1.5124364069
C -2.0713680145 -4.8161999157 3.2044434147
H -1.6095163751 -3.8363937115 3.3852936144
C -1.0901545071 -5.6261043431 2.3422533232
H -0.1262977123 -5.7206444976 2.8495058759
H -0.9048538968 -5.1628192015 1.3695713807
H -1.455534313 -6.6364077657 2.1567872311
C -2.2626002477 -5.4806559206 4.5793362509
H -1.305278136 -5.5668301466 5.0991738762
H -2.6643524321 -6.4903226706 4.4787004195
H -2.9373756714 -4.9178294211 5.227206983
C -4.8745483814 -5.8481311438 1.9646315985
H -5.6481743708 -5.4072896498 1.3214342402
C -5.5983287228 -6.403720075 3.2042528867
H -6.324281967 -7.1646424082 2.9087345955
H -6.1402048696 -5.6315796536 3.7522745642
H -4.9010525925 -6.8760867674 3.8978028681
C -4.2016110045 -6.9820392041 1.1746105613
H -4.9479501765 -7.695057694 0.8165372998
H -3.5041580542 -7.5385619535 1.8025268584
H -3.6441585349 -6.6241467991 0.305003891
C -4.6498557557 -3.0663368422 3.358205135
H -4.9165993589 -3.5491680411 4.3058682948
C -5.9536018583 -2.640256594 2.6644986125
H -6.4962555377 -1.9141406352 3.2744486921
H -6.6261183887 -3.481101294 2.4881579018
H -5.7543441038 -2.1644321342 1.6994001947
C -3.7859332773 -1.8404258881 3.6906276602
H -4.3516098069 -1.1262667108 4.2941111264
H -3.4741760734 -1.312079049 2.7839240307
H -2.8893803629 -2.1033880457 4.2554908601
C 0.3073196538 -2.5148567866 -2.7312076136
H 0.5242001604 -2.7279815412 -3.7795653022
H -0.2932491996 -3.3379147408 -2.3421660602
C 1.5531489252 -2.3720076689 -1.9067604528
C 2.7690574085 -2.970229321 -2.1664129479
H 2.8842934981 -3.610612442 -3.0310269144
C 3.8404835451 -2.7146728587 -1.3119247024
H 4.7959660464 -3.1743602808 -1.5286316535
C 3.7066248356 -1.8592775824 -0.2145789224
C 2.4369118496 -1.3241014651 -0.0137800835
H 2.2177120748 -0.6576653756 0.8063443451
C 6.7470182713 -1.9355352158 0.1947929405
H 6.5501949604 -2.9926568685 -0.0289064683
C 7.1350493579 -1.2419113659 -1.1220292139
H 7.9715002419 -1.7609883504 -1.5957195517
H 7.4572166322 -0.2148119878 -0.942615708
H 6.317700104 -1.205557249 -1.8468706976
C 7.9254072619 -1.9057758535 1.1853319926
H 8.8087420895 -2.3632227631 0.7339429387
H 7.7107147343 -2.4503022673 2.1053686205
H 8.1956946586 -0.8843407393 1.4581734188
C 4.8109651259 -2.1400470162 2.6321824537
H 5.6480990726 -1.8229728747 3.2652893978
C 4.8606040783 -3.6729966837 2.5220127072
H 4.7461111756 -4.1342560795 3.5059663362
H 5.8024358343 -4.0319225697 2.1044572182
H 4.0507241323 -4.0522675972 1.8916982458
C 3.5144912461 -1.689763084 3.3236213032
H 3.4306400093 -2.1540281503 4.3095400513
H 2.629896228 -1.9907322476 2.7549106462
H 3.4711683029 -0.6084226585 3.4709034798
C 4.9255192841 0.578181036 1.0836779818
H 3.9861166934 0.710956234 1.6353226469
C 4.7739880091 1.3104906825 -0.2587305256
H 4.6089498972 2.3779749142 -0.0955364823
H 3.9296778957 0.938614128 -0.8457971764
H 5.6676505334 1.2132321173 -0.8752428815
C 6.0373418548 1.2214813323 1.930158787
H 5.8167745255 2.275892494 2.1140993889
H 6.9999715468 1.1781753966 1.4177102514
H 6.1560699667 0.7384027122 2.9019292574
O 0.0155088861 -1.0698500939 2.0579018132
O -0.2485307618 -0.0772689054 1.0544060234
H -0.0950084849 -0.5514114534 2.8708637738

134
4-2a-2+ 2 4 -4264.7444956 1.1720435 0 B3LYP-D3/def2TZVP

Fe 0.6929932043 -0.8644880104 -0.6781578006
N 0.4848810258 1.0294148752 -1.3032702807
Si -0.6834323452 4.7267810231 0.1930660369
C 0.163412794 2.066862456 -0.5181697134
H 0.1163273409 1.8334948348 0.5367445532
Si -3.7543617592 -3.2189896181 2.3168790475
N 0.4525814003 -1.2508332145 -2.6666247193
C -0.0984505453 3.344058377 -1.0075601219
Si 6.187726098 -2.255795087 0.7633212919
N -1.0872973007 -1.8234814217 -0.5479945321
C 0.0125185288 3.4945988904 -2.3940689651
H -0.1744407422 4.4583435697 -2.8502485527
N 2.4463326877 -1.8001428047 -0.9982785429
C 0.353752395 2.4221948103 -3.2177425237
H 0.4335791978 2.5460995686 -4.289746277
C 0.5817819911 1.1896792493 -2.6402011824
C -0.396473088 6.3791974374 -0.6814536538
H -0.8829753107 6.256024711 -1.6585436889
C 1.0831946389 6.7076964549 -0.9380618287
H 1.1736247793 7.579392388 -1.5903462815
H 1.625954655 5.885022163 -1.4111081783
H 1.6003430865 6.9491015062 -0.0083120053
C -1.1050566458 7.5545586459 0.0156316117
H -0.9905531738 8.4673660132 -0.5734519239
H -0.6810790182 7.7519790046 1.0012160361
H -2.1748909626 7.3814397657 0.1428597625
C 0.2433459859 4.4296769547 1.822631655
H -0.1132122302 3.4466247949 2.1583462171
C -0.1554999813 5.4420492728 2.9109264487
H 0.273522358 5.1567057643 3.8745152089
H -1.2369809451 5.5120650535 3.0424690408
H 0.2164335148 6.4410268334 2.677736391
C 1.7723635378 4.3422974404 1.6946718974
H 2.2184471356 4.0478702301 2.6483619515
H 2.207310642 5.3039280994 1.4211285473
H 2.0920375131 3.6154659021 0.9435626528
C -2.5428280919 4.4100368246 0.4313511975
H -2.8725435363 5.1888139485 1.1291155474
C -3.3317953651 4.5873673692 -0.8752159119
H -4.4026686838 4.4572888051 -0.7009168799
H -3.0352790598 3.8451586993 -1.6230999706
H -3.1930809247 5.5758210499 -1.3151205802
C -2.8572417216 3.0498233484 1.0727779357
H -3.933358698 2.9371993115 1.2268197398
H -2.3772083318 2.9209289449 2.0444027213
H -2.5418749041 2.2212134364 0.4299599759
C 0.9853221934 -0.0541695288 -3.3870837782
H 0.6456828041 -0.0425779326 -4.4246623845
H 2.0741496905 -0.1359517477 -3.3935197172
C -1.0048546753 -1.4432069818 -2.9271647416
H -1.1560799174 -2.023055252 -3.8399868124
H -1.4412197122 -0.4561411368 -3.0922474311
C -1.678810039 -2.0674204063 -1.7363386123
C -2.8741128452 -2.7541350218 -1.8025080804
H -3.3306972897 -2.9619262498 -2.761439701
C -3.4876403649 -3.1475246541 -0.6151302384
H -4.4320530217 -3.6737645903 -0.6692307522
C -2.9109978822 -2.8676873329 0.6273573954
C -1.6826764628 -2.2121250585 0.5910072095
H -1.1290720246 -1.9814963632 1.488635347
C -2.3589741435 -3.6454582632 3.5310705692
H -1.7806507322 -2.7159721838 3.6148742671
C -1.3958267033 -4.7434552343 3.053319661
H -0.5674997216 -4.8574407033 3.7577559583
H -0.9635849674 -4.5279157169 2.0731060322
H -1.8933133551 -5.7111940726 2.9843397986
C -2.8982541287 -3.9677930853 4.93537076
H -2.07670926 -4.0624588086 5.6496047491
H -3.4391950416 -4.9152518444 4.9406913183
H -3.5735062145 -3.1975831216 5.3132377249
C -5.0666944457 -4.5485022444 2.0165184378
H -5.648950302 -4.1663308622 1.1672227397
C -6.0528786827 -4.7008926913 3.1891056889
H -6.8474102442 -5.4023314518 2.9243396719
H -6.5291608242 -3.7575114916 3.4612423784
H -5.5607826921 -5.0932813989 4.0802368469
C -4.4921595579 -5.9149683853 1.6080239026
H -5.2918052179 -6.5832577596 1.2800489648
H -3.9950914159 -6.4011123992 2.4489467553
H -3.7657765362 -5.8456819056 0.7940450399
C -4.56945307 -1.5672938751 2.7923075264
H -5.040578514 -1.7535253637 3.764932944
C -5.6765779112 -1.1693011507 1.804125715
H -6.1724413905 -0.2508546912 2.1279425045
H -6.4459146375 -1.9366810186 1.7101137963
H -5.2702138044 -0.9797389945 0.8057200663
C -3.5679372778 -0.4166935467 2.9740854599
H -4.0831282922 0.4925445416 3.2940252358
H -3.0583957245 -0.1767964559 2.0354746866
H -2.806501121 -0.6381946633 3.7242805185
C 1.2323321727 -2.488345715 -2.9549911062
H 1.4103604528 -2.5937851454 -4.0275192441
H 0.6214732762 -3.3349708951 -2.6340280426
C 2.5119022598 -2.474642474 -2.1660017924
C 3.671186189 -3.1199364541 -2.5433681506
H 3.7163738267 -3.653730187 -3.4835558316
C 4.773708198 -3.0694942796 -1.6924845877
H 5.6806115744 -3.5794710355 -1.9902593639
C 4.7293066214 -2.3665350697 -0.4843559507
C 3.5192974602 -1.7417333838 -0.1946070061
H 3.3735319551 -1.1663241661 0.7066574123
C 7.7496964638 -2.7841955231 -0.1663858515
H 7.4816412227 -3.7500208284 -0.61560199
C 8.1604243407 -1.8378207471 -1.3065309157
H 8.9532211013 -2.2870102425 -1.9093064492
H 8.5512315928 -0.8968987353 -0.9160365096
H 7.3341598728 -1.594412406 -1.979783965
C 8.9402983058 -3.0536942794 0.7716559212
H 9.7819737699 -3.4603036974 0.2063824777
H 8.6983579168 -3.7711041204 1.5569983852
H 9.2873603197 -2.1383247039 1.2530559365
C 5.7793279253 -3.4976711994 2.1418011872
H 6.617829047 -3.4237118339 2.8444011832
C 5.7297350935 -4.9421440176 1.6192079816
H 5.5513968092 -5.6431195315 2.4381623852
H 6.6596950778 -5.2417817236 1.1339809461
H 4.9179172 -5.0773740723 0.8980191936
C 4.4962761426 -3.1581446186 2.9163770498
H 4.337345929 -3.876026985 3.7249029038
H 3.6131571024 -3.2061949221 2.2712456049
H 4.5351856097 -2.1651348125 3.3694200695
C 6.1486811609 -0.4818261223 1.442259123
H 5.1996732854 -0.4238046784 1.9924628392
C 6.1269530642 0.6250479634 0.3767824283
H 5.9848593427 1.6030707416 0.8440305065
H 5.3240066714 0.4892244815 -0.3525580796
H 7.0649765898 0.6673244074 -0.1772174657
C 7.2721522361 -0.2356947 2.463940305
H 7.1397929873 0.7307166086 2.9564962475
H 8.2490955568 -0.217245423 1.9784963039
H 7.3032628228 -0.9990621677 3.2435893148
O 1.1118808628 -1.5743795027 1.8334037533
O 0.9365389757 -0.4032145692 1.0144499538
H 1.4726799467 -1.1876818062 2.647241115

134
4-3-2+ 2 4 -4264.7561668 1.1735945 0 B3LYP-D3/def2TZVP
Fe -0.1582904735 -1.4794416329 -0.9772937169
N -0.0588943919 0.4299510072 -1.6509915831
Si 0.3505286741 4.3196912702 -0.126749959
C 0.0316133174 1.4952859559 -0.8308906607
H -0.0679146956 1.2642145743 0.2193546801
Si -5.6047456785 -0.6190716672 0.8824104942
N -0.1803469945 -1.8939393645 -3.0416915771
C 0.2514087637 2.7888181607 -1.288121074
Si 5.3689204633 -1.0448852832 0.85012907
N -2.1225523486 -1.4505460695 -1.2479554304
C 0.3963847133 2.9293443721 -2.6757886674
H 0.6022555314 3.9016197918 -3.1044527852
N 1.8081546704 -1.5598014241 -1.2622170042
C 0.281273654 1.8379996915 -3.5229569425
H 0.3747861431 1.9490690694 -4.5956301979
C 0.042462949 0.5882339962 -2.9769999568
C 1.8717688595 5.293359517 -0.7041924802
H 1.6006306438 5.6284311057 -1.7137221556
C 3.1669212612 4.4776322617 -0.8266984919
H 3.9520979605 5.0770951357 -1.2938394714
H 3.0407380428 3.5761358757 -1.4318215938
H 3.5375478129 4.1683821776 0.1497932542
C 2.1033055424 6.5594213014 0.1404893766
H 2.8845254142 7.1777601817 -0.307502484
H 2.4323957376 6.3078266371 1.150719825
H 1.20759164 7.1774034498 0.2258554352
C 0.3744369496 3.6578510196 1.6512441939
H -0.4556759311 2.9390706244 1.6905769484
C 0.0677669916 4.7433895588 2.6985809872
H 0.0145216012 4.3038329541 3.6976628776
H -0.8814074847 5.2472111868 2.5130784827
H 0.8468947552 5.5065562629 2.7234048195
C 1.6643323096 2.9094989803 2.0206145646
H 1.5503480085 2.3846414505 2.9725883973
H 2.4936141628 3.6078944306 2.136632409
H 1.9667845031 2.1759792742 1.2691757048
C -1.2276033357 5.3290362666 -0.4287635435
H -1.1327271134 6.1856876007 0.2493606206
C -1.3620995221 5.8819180893 -1.8549990081
H -2.2410438007 6.5263499681 -1.9320903125
H -1.4982739833 5.078736865 -2.5853434923
H -0.5002403498 6.4766790626 -2.1610435485
C -2.4896872285 4.5536695948 -0.0290294562
H -3.3798989998 5.1787175743 -0.1353361482
H -2.4564092557 4.2085877201 1.00441684
H -2.6367655005 3.6765506364 -0.666173228
C -0.1680379311 -0.6268177757 -3.831895432
H -1.1270525486 -0.5239662326 -4.3425744987
H 0.5945303797 -0.6810679868 -4.6097328655
C -1.4476506887 -2.6471030518 -3.2401168829
H -1.2899215276 -3.6628174488 -2.8783825581
H -1.7248021618 -2.6977153524 -4.2945859655
C -2.5280971405 -2.0216018819 -2.4027455225
C -3.8753277521 -2.10054243 -2.6937783252
H -4.1988178752 -2.5400431163 -3.6282932065
C -4.7974547711 -1.6471904516 -1.7554168744
H -5.8515936157 -1.7371932474 -1.9822601798
C -4.3873835714 -1.1125714713 -0.5302974367
C -3.0095557819 -1.0157343206 -0.3427385338
H -2.5760111107 -0.6106912277 0.5607150108
C -4.8213018739 -1.3088944219 2.4681716817
H -3.9009410263 -0.7222332861 2.5892436674
C -4.4126322606 -2.789480569 2.4136171909
H -3.8816957417 -3.0701469165 3.326850712
H -3.7537742124 -3.0133901604 1.5705040031
H -5.2796776428 -3.4449307002 2.3320019727
C -5.6988404039 -1.0306834406 3.7016308953
H -5.1685935979 -1.3017365651 4.6176296248
H -6.6162689196 -1.6213466124 3.6745777873
H -5.9825442875 0.0202424893 3.7865327462
C -7.3011548986 -1.307809475 0.3934088922
H -7.4452981266 -0.9792632867 -0.6447478418
C -8.4497254713 -0.6883714103 1.2106833013
H -9.4109218005 -1.0641222293 0.8524837121
H -8.4772954531 0.3994249106 1.1339927439
H -8.3766234092 -0.9464369209 2.2683029058
C -7.3859610077 -2.84251714 0.4191421802
H -8.3214910414 -3.1822561598 -0.0310738063
H -7.3693029882 -3.2171523317 1.4435864673
H -6.5664422885 -3.3272350804 -0.1175782361
C -5.6549216576 1.2748783346 0.9382139973
H -6.4317225334 1.5092114937 1.6752684252
C -6.0866225867 1.8779518124 -0.407216534
H -6.1828911631 2.9638164199 -0.332648999
H -7.0480479178 1.4895525013 -0.7472996518
H -5.3490978805 1.6715398188 -1.1888900584
C -4.3498244675 1.9137774473 1.4295110403
H -4.4583834629 2.9983199915 1.4924451898
H -3.5228506457 1.7211957811 0.7385611307
H -4.0550273818 1.5587960763 2.4183279242
C 1.0472427405 -2.7063983192 -3.2544293109

H 1.3184122323 -2.7526383515 -4.3106281525
H 0.8349083157 -3.719048234 -2.9123142585
C 2.1679878077 -2.1627640351 -2.413352908
C 3.50544822 -2.3487094178 -2.7039131158
H 3.7934601139 -2.8219088548 -3.6335524944
C 4.4587987062 -1.9578257835 -1.77071499
H 5.5023441556 -2.1389323305 -1.9916406381
C 4.093280851 -1.3577460709 -0.5599253775
C 2.7271774985 -1.1603511077 -0.3711559087
H 2.3249473337 -0.6975066748 0.5186834529
C 7.0722583266 -0.9229259661 0.0292653184
H 7.2110900838 -1.906441707 -0.4384815289
C 7.2126276169 0.1356025757 -1.0773425388
H 8.1577227424 0.0030999195 -1.609424954
H 7.2157384281 1.1454144886 -0.6678751044
H 6.4118535209 0.0908021713 -1.819467742
C 8.1935365745 -0.7673870335 1.0732586639
H 9.1722795066 -0.8464808318 0.5948593527
H 8.1486125721 -1.5299787444 1.8528619649
H 8.150711461 0.2095666036 1.558463145
C 5.2482910849 -2.611752751 1.9170723554
H 5.9413663077 -2.4343744033 2.7479817869
C 5.7108792173 -3.8792781202 1.184193502
H 5.6444673514 -4.7492929214 1.8419752637
H 6.7451700646 -3.8102567586 0.8447100703
H 5.0850624598 -4.0907472863 0.3113873648
C 3.8470692505 -2.8112074144 2.5158029669
H 3.8407783618 -3.6603245548 3.2032863166
H 3.1105258354 -3.0296339891 1.7359570087
H 3.4969568837 -1.9397855987 3.0728060437
C 4.7937858622 0.4704430889 1.833890541
H 3.7101437367 0.3445635535 1.9632062673
C 5.0239190108 1.795851758 1.0947032111
H 4.5773602663 2.6223220381 1.6497651701
H 4.5979893151 1.80216216 0.0886103108
H 6.08818066 2.0143017577 1.0036165103
C 5.4034905092 0.5305399203 3.2462231499
H 4.9957953233 1.3820144307 3.7967823726
H 6.4866495007 0.6589106729 3.2117785609
H 5.1950809888 -0.3664793586 3.8301622858
O -0.2007044012 -3.2494692809 -0.71714289
O -0.143970133 -1.0254257103 0.5875872572
H -0.2005990069 -3.4797441212 0.2228988937

134
2-3-2+ 2 2 -4264.7360293 1.1730293 0 B3LYP-D3/def2TZVP
Fe -0.158843186 -1.7343335406 -0.6681211815
N -0.0710474856 0.1160301985 -1.4632444305
Si 0.2911728597 4.1171721354 -0.2377873899
C 0.0063485134 1.2426094122 -0.7266585812
H -0.0956881209 1.0930369268 0.3381364689
Si -5.6196240336 -0.7752108019 1.1001165592
N -0.1682057904 -2.3033788097 -2.6804220399
C 0.2149242833 2.5005313251 -1.2777956665
Si 5.3602506823 -1.0958396206 1.1322376596
N -2.1182543871 -1.7355524403 -0.9458051125
C 0.3640691177 2.5391658376 -2.6717205885
H 0.5620579232 3.4786969324 -3.1711706602
N 1.8074108566 -1.8122316264 -0.9365860656
C 0.2622907213 1.3864080476 -3.4345890018
H 0.358545583 1.4177404689 -4.5122864914
C 0.0330588045 0.1787766302 -2.7972802903
C 1.8031700901 5.0629693071 -0.8820758128
H 1.5318650601 5.3184093718 -1.9145527816
C 3.1082886195 4.2561569884 -0.9396260243
H 3.8879563477 4.8282497263 -1.4484556172
H 2.9948654165 3.3102575352 -1.4755080265
H 3.4790644439 4.0258005405 0.0583290665
C 2.0166718569 6.3912508192 -0.1337280011
H 2.7920705029 6.9838729122 -0.6245395105
H 2.3451822839 6.2196694371 0.8932963899
H 1.1134373145 7.0030381574 -0.0969707386
C 0.3143669227 3.5943963737 1.5860956118
H -0.5083897231 2.871807535 1.6773308129
C -0.0079031168 4.7530359759 2.5466957444
H -0.06188864 4.3901136819 3.5760650486
H -0.9609248463 5.2317991706 2.3193928787
H 0.7636873463 5.5236307251 2.5168224386
C 1.6106063646 2.890355744 2.0156910279
H 1.4984845847 2.438479757 3.0046498523
H 2.4320058135 3.6045127886 2.0808702321
H 1.9241542135 2.105149605 1.3233213987
C -1.2964866334 5.0826030659 -0.6225495753
H -1.2131442951 5.9904481012 -0.0129029873
C -1.4315447334 5.5215757306 -2.0878062043
H -2.3164202436 6.1493433001 -2.2169454181
H -1.5574539315 4.6627311675 -2.7537854116
H -0.5745111729 6.0992829423 -2.4370059835
C -2.55196986 4.3275377639 -0.1675036848
H -3.4481837743 4.9329832623 -0.3247031219
H -2.5190401036 4.0642707428 0.8897143447
H -2.6877134072 3.4020682532 -0.7349394622
C -0.163643638 -1.0969938318 -3.5616931322
H -1.1215440387 -1.0400881694 -4.0815077809
H 0.6019876041 -1.2014519609 -4.3313167665
C -1.4274500933 -3.0798627169 -2.8321139749
H -1.2657396795 -4.0644754327 -2.3936916724
H -1.6953450659 -3.2132851585 -3.8817708598
C -2.5170488655 -2.3985159118 -2.0524204451
C -3.8620105227 -2.5096815576 -2.343952834
H -4.1772728179 -3.0237854906 -3.2424939234
C -4.7923262037 -1.9901369558 -1.4493904739
H -5.8445840968 -2.1047074977 -1.6736153215
C -4.3914308602 -1.3579805526 -0.2683550319
C -3.0154501371 -1.2353628522 -0.0844021465
H -2.5920614659 -0.7545058897 0.7861080803
C -4.8392531894 -1.347273804 2.7339086694
H -3.9243303498 -0.7458042544 2.8182154525
C -4.4182063734 -2.8244717343 2.7846117662
H -3.8897376545 -3.0364307168 3.7176430083
H -3.753425246 -3.1008761345 1.961916442
H -5.2793533571 -3.4914422666 2.7449214879
C -5.7257268824 -0.9914577117 3.9407119961
H -5.1986130761 -1.1938009065 4.8760838851
H -6.6383135027 -1.5901239547 3.9500686837
H -6.0182651194 0.0604120905 3.9508729187
C -7.3085364751 -1.5101250183 0.6537927094
H -7.449190373 -1.258302167 -0.4060601345
C -8.4663949064 -0.842578697 1.4183629292
H -9.4225974755 -1.2514685157 1.0838855558
H -8.5023456812 0.2364573835 1.2625247611
H -8.3969312951 -1.022395806 2.492325513
C -7.3824292484 -3.0396120734 0.7896677713
H -8.3127486523 -3.4180703612 0.3600018868
H -7.3693241443 -3.3392287728 1.8385744505
H -6.5562682073 -3.5555919764 0.293867937
C -5.686141385 1.1175199916 1.0264199524
H -6.4736372641 1.394089673 1.7369642007
C -6.107418076 1.6231448738 -0.3618075778
H -6.2146835794 2.7105355623 -0.3626962228
H -7.0611740373 1.2027362512 -0.6852288831
H -5.3588818391 1.3712034152 -1.1192424749
C -4.3928114205 1.8006557745 1.4880555239
H -4.5111821351 2.885913487 1.4748466318
H -3.5555328328 1.56867207 0.8221716042
H -4.1079533214 1.5172870745 2.5025456319
C 1.0668678469 -3.1172429915 -2.8333707838
H 1.3382684756 -3.2414980001 -3.8832171158
H 0.8653100457 -4.1036093066 -2.4156554685
C 2.1798647878 -2.4970386451 -2.0364151639
C 3.5199486341 -2.6874827714 -2.3123429855
H 3.8150606829 -3.2269960591 -3.202775493
C 4.4670585941 -2.2130489071 -1.4125195257
H 5.5132489537 -2.3959650426 -1.6188256732
C 4.0910577529 -1.5267125249 -0.2516751136
C 2.7224289554 -1.3324036428 -0.0807859087
H 2.3156680974 -0.8052849356 0.7704309677
C 7.063921241 -1.0129315491 0.3073258879
H 7.2156900241 -2.0261407197 -0.0872634643
C 7.1938170209 -0.0364611999 -0.8735275303
H 8.1418576845 -0.1955489787 -1.39293852
H 7.1833167454 1.0005559714 -0.5390542864
H 6.3955451633 -0.145701322 -1.61160952
C 8.1808866099 -0.7679552292 1.3386225703
H 9.161581366 -0.8696280443 0.8685634029
H 8.1435388894 -1.4722107556 2.1716882294
H 8.1251533674 0.2411423353 1.7513051538
C 5.2577949992 -2.5826583251 2.3104482705
H 5.9456417786 -2.3357685906 3.1277832116
C 5.7397883576 -3.8937991432 1.6730391696
H 5.6832671333 -4.7144358232 2.3923504739
H 6.7740627772 -3.8353631583 1.3316092711
H 5.1198093394 -4.1768952174 0.8164999071
C 3.8575853046 -2.7573503573 2.9191378279
H 3.8602916149 -3.5545074035 3.6662912253
H 3.126756376 -3.0415849235 2.1551249251
H 3.4940063965 -1.8526698423 3.4106948435
C 4.7660136315 0.4790979271 2.0048651858
H 3.6834092962 0.3511726691 2.1408333349
C 4.9840435842 1.7507051249 1.1733686949
H 4.5278590202 2.6099563386 1.6674550327
H 4.5602746168 1.6806864344 0.1688378551
H 6.0461979779 1.9731301924 1.0684395273
C 5.3713280898 0.6466845933 3.4104150461
H 4.9537924163 1.5314425671 3.8974640571
H 6.4532387574 0.7831449594 3.3689589826
H 5.1703024722 -0.2079876319 4.0570763031
O -0.1866281716 -3.476966089 -0.260041327
O -0.1528154425 -1.167978215 0.8717073703
H -0.1581526334 -3.6431446884 0.6940356574

134
4-3a-2+ 2 4 -4264.7553734 1.1742051 0 B3LYP-D3/def2TZVP
Fe 0.0788947301 -2.0298486188 -0.3756249069
N 0.0041873449 -0.1967558398 -1.3168851503
Si -0.1931299615 3.8749512481 -0.3737403007
C -0.0451199599 0.983613397 -0.6766539295
H -0.0695283063 0.9071817023 0.3998082437
Si -5.5355472579 -0.771954996 0.8278232708
N 0.1132636881 -2.7293099404 -2.3220776706
C -0.0581794834 2.2110318944 -1.3312665601
Si 5.5229342744 -0.5068978019 1.0940979397
N -1.8860141709 -2.1480463027 -0.7021807571
C -0.0114158306 2.1505764662 -2.7306259848
H -0.007886665 3.0612726642 -3.3154694146
N 2.0536349724 -1.9848500547 -0.6780752141
C 0.029631595 0.9330171777 -3.3963607782
H 0.0581109001 0.8895996634 -4.4776618703
C 0.0327866484 -0.2354995305 -2.6522075562
C 0.8411849554 5.1319936189 -1.3411685434
H 0.4129972372 5.1051836205 -2.3520250731
C 2.3302262146 4.7755621721 -1.464401143
H 2.8275408897 5.4476509045 -2.1677675034
H 2.4947585138 3.7536194638 -1.8142608561
H 2.8411880034 4.8822633699 -0.5063718766
C 0.6647242465 6.5706726468 -0.8248510824
H 1.17959423 7.2748246818 -1.482523936
H 1.0901336073 6.6931114811 0.1727117133
H -0.3825325878 6.8738874272 -0.7800150473
C 0.3088976796 3.5136201572 1.4203107689
H -0.3749587946 2.7099610869 1.725668367
C 0.0415456296 4.7028719979 2.359919664
H 0.2141461343 4.4147301836 3.3998298677
H -0.9828779407 5.0715787776 2.2889612752
H 0.7087257203 5.5384286689 2.1427788093
C 1.7398800339 2.9917092619 1.6045793175
H 1.8875208128 2.6225307213 2.623142248
H 2.4738117836 3.7813103403 1.4419427903
H 1.9852985254 2.1806065242 0.9167168235
C -2.0282987158 4.3542295441 -0.4191578128
H -2.0792231526 5.3300514205 0.0773090595
C -2.5721021895 4.5286457663 -1.8453236578
H -3.609067656 4.8723055078 -1.8228078477
H -2.5621350955 3.5830169328 -2.3954656233
H -2.0027289304 5.2593842426 -2.422265101
C -2.9004221507 3.3764499656 0.3784287798
H -3.9480837487 3.6853457511 0.3545775551
H -2.6087480306 3.3102079092 1.427413741

H -2.8495815725 2.3702440262 -0.0484805288
C 0.0465923639 -1.5931922991 -3.3044177728
H -0.8551102527 -1.7083749757 -3.9071335087
H 0.8935247847 -1.6653619618 -3.9873765309
C -1.0810489331 -3.6230614295 -2.4121182682
H -0.8399346392 -4.5444104024 -1.8816410004
H -1.3063774941 -3.8727932043 -3.4500952655
C -2.2305007977 -2.958324311 -1.7204460627
C -3.561596171 -3.1756632066 -2.0178171555
H -3.8321913244 -3.8300974839 -2.8360342841
C -4.5303146182 -2.5531197478 -1.2402181059
H -5.5720497231 -2.7507854798 -1.4545085706
C -4.1822326872 -1.6778529964 -0.204581942
C -2.8173481328 -1.5099000606 0.0201223529
H -2.42863799 -0.8668268532 0.7954710203
C -4.6196076659 0.3841289119 2.0266201384
H -3.8288195818 0.8418235249 1.4188965011
C -3.9455811876 -0.3272581099 3.2130830546
H -3.2992067293 0.3665914075 3.7571206927
H -3.3372004712 -1.1861526162 2.9181486283
H -4.6887896764 -0.6931129702 3.9227411033
C -5.5037677147 1.5367706651 2.5359094811
H -4.9272543497 2.1965189455 3.1892816844
H -6.350421132 1.1673508196 3.1169824817
H -5.8997345703 2.1478667252 1.7245751694
C -6.5816586732 -2.1442426504 1.6078186969
H -7.0594283164 -2.6348021234 0.7498578003
C -7.7027653939 -1.5608737811 2.4859110107
H -8.3720027327 -2.3536522603 2.8277811114
H -8.3122144349 -0.8266529141 1.9550166272
H -7.2966990422 -1.0756311808 3.3760562384
C -5.7899655069 -3.2220645408 2.3630858198
H -6.4547758067 -4.0305124514 2.6761816253
H -5.3225351786 -2.8240922422 3.2639906171
H -5.0031711773 -3.666632815 1.7489115214
C -6.5688760115 0.2553884855 -0.394629979
H -7.200941508 0.8651043804 0.2620647981
C -7.5078369783 -0.5512628738 -1.3029139949
H -8.1517373615 0.1219922482 -1.8737376642
H -8.1595088853 -1.2256437413 -0.7461752592
H -6.9527110129 -1.1427157583 -2.0356695685
C -5.702747647 1.2122270751 -1.2267001766
H -6.3268009221 1.8559773502 -1.8513903296
H -5.0362993836 0.6611308491 -1.8976164114
H -5.0856988558 1.8607084429 -0.6058798995
C 1.3920195081 -3.4932155229 -2.4234074256
H 1.6420443139 -3.7025846225 -3.4645816713
H 1.2452567021 -4.4434838698 -1.9093048711
C 2.4718991725 -2.7358776672 -1.7159598384
C 3.8168548538 -2.8320104844 -2.0092971784
H 4.144292008 -3.427918915 -2.8511479009
C 4.7300335636 -2.1745978888 -1.1918287037
H 5.7849544741 -2.2763900584 -1.4084415411
C 4.3079381964 -1.3932144558 -0.1126694951
C 2.9281303898 -1.316990652 0.0852051837
H 2.4852003813 -0.7247399281 0.872896555
C 7.2740257845 -0.7719022565 0.4219860994
H 7.3286861412 -1.8452920618 0.1966541958
C 7.5833596032 -0.0005020445 -0.8721090585
H 8.5397269243 -0.3233253135 -1.2899276496
H 7.6649766495 1.0698531053 -0.6768634624
H 6.824173119 -0.1314694161 -1.6472731272
C 8.3579536575 -0.48986755 1.4800711873
H 9.3460765253 -0.7178261996 1.0739452575
H 8.2310663845 -1.0904038607 2.3811798453
H 8.3665809296 0.559705873 1.7780807119
C 5.33396704 -1.3855297088 2.7664812457
H 6.0553804207 -0.8776112212 3.4177163713
C 5.7376556235 -2.8663562918 2.6807696537
H 5.7013098512 -3.334799008 3.6671255249
H 6.7485629065 -3.0038256663 2.2948112424
H 5.0560565444 -3.4260152147 2.0331819448
C 3.9506043703 -1.2417832153 3.417509222
H 3.9574765306 -1.6658589491 4.4243741405
H 3.1869945724 -1.7882781965 2.855083803
H 3.6310160822 -0.2015958038 3.5058825885
C 4.9226065619 1.2882030222 1.1808901565
H 3.9505168424 1.2241938765 1.6849382387
C 4.6862832909 1.9464440596 -0.1871672355
H 4.2771080664 2.9499531763 -0.061609612
H 3.9811382196 1.3833779245 -0.804585542
H 5.6108293691 2.046766732 -0.7556607296
C 5.8287240688 2.1662723186 2.0607658667
H 5.3725659342 3.1466653522 2.2193074664
H 6.7976757919 2.3353984856 1.5876994083
H 6.0108455648 1.7281169225 3.043969155
O 0.1428827262 -3.554106983 0.2268853141
O 0.0356789779 -1.1858383729 1.1814931605
H 0.0594859496 -1.8022180502 1.9291903712

134
2-3a-2+ 2 2 -4264.7361169 1.1737163 0 B3LYP-D3/def2TZVP
Fe 0.0754469699 -2.0293641287 -0.3777809435
N -0.0025566547 -0.2043299893 -1.3059134604
Si -0.190883151 3.8703866891 -0.3681203645
C -0.0482683489 0.9777001367 -0.66772633
H -0.0681274051 0.9041081264 0.4088802351
Si -5.5338430517 -0.7620471042 0.8338045045
N 0.1032152962 -2.7360583673 -2.3101662302
C -0.063240652 2.2046537066 -1.3232000412
Si 5.5226740105 -0.5086059435 1.0777777597
N -1.8857981232 -2.1515629728 -0.6926177497
C -0.0227597098 2.1432408855 -2.7226613323
H -0.0211117869 3.053355344 -3.3083835749
N 2.0447642375 -1.9866377191 -0.6820822155
C 0.0144584199 0.9249666158 -3.3869631664
H 0.0381864016 0.8803379286 -4.468324907
C 0.0200850962 -0.2428384633 -2.6417430604
C 0.8408181906 5.1242209189 -1.342707466
H 0.4088268001 5.0947445334 -2.3518743221
C 2.3290617134 4.7662829818 -1.4705633972
H 2.8242572762 5.4357592751 -2.1779121065
H 2.4914516241 3.7431175564 -1.8177612918
H 2.8436689525 4.8756894181 -0.5147971357
C 0.6676346129 6.5647549488 -0.8304823376
H 1.1807715538 7.2661818805 -1.4924255045
H 1.0968416538 6.690289983 0.1650883263
H -0.3791368723 6.8691687972 -0.7828979033
C 0.3190925442 3.5118945499 1.4243363339
H -0.3655123601 2.7108523984 1.7349091109
C 0.0597341262 4.7041249837 2.3623332338
H 0.2367588891 4.4182450856 3.4021358309
H -0.9640043494 5.0754819648 2.2955073056
H 0.7280655781 5.537279704 2.1396168657
C 1.7494083803 2.9863420511 1.6028018581
H 1.9010064208 2.6187789859 2.621401119
H 2.4848614689 3.7734091964 1.4350089742
H 1.9891849484 2.1733230504 0.9152031211
C -2.0249286692 4.3545722276 -0.4051006643
H -2.0704640354 5.3329439079 0.0868285803
C -2.5765251748 4.5233639929 -1.8289842852
H -3.6121670001 4.8706997996 -1.8022111182
H -2.5729327659 3.5748574418 -2.3741861829
H -2.0080964517 5.2491257766 -2.4131175832
C -2.8947951035 3.3828548642 0.4021180996
H -3.9422990322 3.6925222828 0.3808181821
H -2.5988318756 3.3227004917 1.4502619112
H -2.846513032 2.3740978117 -0.018898203
C 0.029323758 -1.6004712561 -3.2934688571
H -0.8768186143 -1.7157977778 -3.8894719058
H 0.8707304355 -1.6728959966 -3.9831934485
C -1.0891879498 -3.6315363243 -2.3995530376
H -0.8463912028 -4.5541846872 -1.8715643834
H -1.3176904892 -3.881228738 -3.4368693535
C -2.2364866916 -2.9663702719 -1.7044829405
C -3.5682529668 -3.1860941004 -1.9977733632
H -3.8407113192 -3.8454506529 -2.8113722232
C -4.5349225284 -2.5582919348 -1.2224456412
H -5.5773487351 -2.7562232842 -1.4330627889
C -4.1826801021 -1.6764570462 -0.1936352216
C -2.8178434722 -1.5080712963 0.0266370661
H -2.4287294021 -0.8590847877 0.796647409
C -4.6165767372 0.3917087418 2.0336983207
H -3.8235277209 0.8467049991 1.4270669358
C -3.9463213138 -0.3214213029 3.2211654495
H -3.2979611925 0.3702936075 3.7655791254
H -3.3408617159 -1.1826786869 2.9268477722
H -4.6914244069 -0.6843900772 3.9303197169
C -5.4978786753 1.5473892336 2.5409829857
H -4.9202682919 2.205936179 3.1945800162
H -6.3465790777 1.1811492964 3.1210420451
H -5.8907132174 2.1589800291 1.7284813233
C -6.5887474385 -2.127721084 1.613976424
H -7.0679931705 -2.6164791255 0.7558837608
C -7.7077750343 -1.5375953018 2.4900675519
H -8.3816978046 -2.3264900656 2.8317502321
H -8.3126481888 -0.800620863 1.9577364509
H -7.3002574773 -1.0538127368 3.3803136595
C -5.8036465786 -3.208850309 2.371273151
H -6.4731963713 -4.012879622 2.6856604129
H -5.3340631176 -2.811950919 3.2715361726
H -5.0193771424 -3.6590625093 1.7579531017
C -6.5586077918 0.2678908675 -0.3936999613
H -7.1912612249 0.8802378328 0.2600283403
C -7.4960801925 -0.5369713574 -1.3051147485
H -8.1360233499 0.1375881836 -1.8788515492
H -8.1517362881 -1.2094339999 -0.750663695
H -6.9393442497 -1.1301798126 -2.0352806739
C -5.6862441822 1.2212458654 -1.2232360189
H -6.3059896154 1.8661437742 -1.8510555625
H -5.0187837288 0.6675755792 -1.8909973877
H -5.06990814 1.8686726698 -0.6006839825
C 1.3818052657 -3.4973186385 -2.4227052243
H 1.627454305 -3.7031199706 -3.4656569446
H 1.2406315238 -4.4507619563 -1.9123420492
C 2.4631082983 -2.73906857 -1.7182042595
C 3.8070622269 -2.8362563983 -2.0165347127
H 4.1315316057 -3.4342817711 -2.8579995036
C 4.7228882771 -2.1768600205 -1.2042368079
H 5.7771263006 -2.2788646751 -1.4238733566
C 4.3029757297 -1.3928727129 -0.1259181136
C 2.9243641825 -1.3157176927 0.075155197
H 2.4855902804 -0.7194010261 0.8618986851
C 7.2719529447 -0.7781342985 0.4022861599
H 7.3234658406 -1.8516381496 0.1767925323
C 7.5803918541 -0.0073117187 -0.8923254991
H 8.5351954864 -0.3322382643 -1.3121118099
H 7.6648310515 1.0628369644 -0.6971570262
H 6.8193417206 -0.13652226 -1.6659174016
C 8.3586587435 -0.49886197 1.4582185122
H 9.3453381581 -0.7295725563 1.0500980256
H 8.2320322264 -1.098930677 2.3596821376
H 8.370790976 0.5507066143 1.7560517844
C 5.3357650419 -1.3872761619 2.7506380297
H 6.0596364926 -0.8811329892 3.4004822825
C 5.7360060679 -2.8689293315 2.6636533038
H 5.7007836846 -3.3377607538 3.64987743
H 6.7457638922 -3.0083844372 2.2754528072
H 5.0519539652 -3.4268191446 2.0170887349
C 3.9542522445 -1.240730384 3.4048413638
H 3.9621273257 -1.6654100866 4.4114581779
H 3.1883358464 -1.7851776753 2.8434135151
H 3.6372320751 -0.1998669391 3.4942926355
C 4.9283431766 1.2881929435 1.1672880625
H 3.9578080205 1.2273820896 1.6746140922
C 4.6893974529 1.9472487755 -0.1999054503
H 4.2843436179 2.9522112018 -0.0728475009
H 3.9799223721 1.3866881146 -0.8145727808
H 5.6121777132 2.0442578265 -0.7718423482
C 5.8402797161 2.1632298137 2.0441630656
H 5.3875502433 3.1448861108 2.2047120656
H 6.8079312121 2.3297486249 1.5675594736
H 6.0248003066 1.7242381007 3.0265563075
O 0.1435093743 -3.5607859548 0.2392137259
O 0.0373040767 -1.1951096415 1.1883235813
H 0.0659294767 -1.8082037998 1.9394497465

134

6-2a-2+ 2 6 -4264.741321 1.1706268 0 B3LYP-D3/def2TZVP
Fe 0.4151338366 -0.3751048734 -0.5851978258
N 0.3216902361 1.4479199425 -1.5067795435
Si -0.2873204368 5.4283971483 -0.3666581686
C 0.1454941284 2.5895833542 -0.8117121421
H 0.1127022447 2.4618260373 0.2620137769
Si -3.3302227511 -3.7000917647 2.3972469135
N 0.1399614388 -0.9667217608 -2.7482062189
C 0.0203823373 3.8389773358 -1.4073237089
Si 5.4845764038 -2.6090715295 1.2236671631
N -1.2871217199 -1.5312817119 -0.5093379025
C 0.0782861236 3.8471490607 -2.8068154056
H -0.0157315831 4.7804790762 -3.3472231115
N 2.0554274577 -1.572612046 -0.8981296979
C 0.2562137313 2.6734322892 -3.532763368
H 0.299384826 2.6890174872 -4.6140098339
C 0.3855375693 1.4774077206 -2.8537867128
C 0.5040026193 6.8494258128 -1.3345013416
H -0.0102822114 6.8266313371 -2.3044890817
C 2.0072050238 6.684788871 -1.6073500327
H 2.3566793183 7.4566259844 -2.2971416764
H 2.2530583973 5.7155931038 -2.0488787036
H 2.590918126 6.7852993511 -0.6918528242
C 0.2061332994 8.2238669253 -0.7096552082
H 0.5517990807 9.0235822121 -1.3685587937
H 0.7217273635 8.3476372115 0.2440423788
H -0.8596476817 8.3823296723 -0.5353712465
C 0.3893772362 5.0654597745 1.3690045564
H -0.1511246166 4.1617249695 1.6834067414
C 0.0297823857 6.1653946164 2.3837278209
H 0.3242786624 5.8649898166 3.3923134765
H -1.0398940181 6.3800162708 2.4062646955
H 0.5505404197 7.0978030954 2.1617252603
C 1.8918967029 4.7463955876 1.4241264159
H 2.1700493457 4.3849577408 2.4177782735
H 2.4937122998 5.6346396506 1.2295229662
H 2.1930268203 3.9853009331 0.6994979177
C -2.1739268485 5.630902168 -0.3037884356
H -2.3426208657 6.5247171512 0.3082662302
C -2.7880343456 5.8909077606 -1.6878020958
H -3.8674805 6.0404769136 -1.6078057095
H -2.6336597649 5.0413238707 -2.3605573485
H -2.3745974539 6.7785282603 -2.1689643626
C -2.8735585289 4.4515978211 0.3884983503
H -3.9507387725 4.6247283677 0.4491063255
H -2.5131824153 4.2911668626 1.4062022395
H -2.7307729544 3.520898297 -0.1707277043
C 0.6708443747 0.1625741076 -3.5368507966
H 0.2826188261 0.1593636103 -4.5593033478
H 1.75454025 0.0375057416 -3.6040871377
C -1.3196818626 -1.149442015 -2.9014996182
H -1.5637329217 -1.7066252856 -3.8100452941
H -1.7677962126 -0.1575734741 -2.9987595182
C -1.8899687144 -1.827045005 -1.6794132453
C -2.9744122256 -2.6810808705 -1.7096932425
H -3.4533928352 -2.9155662898 -2.6514139589
C -3.4285322331 -3.2405046079 -0.5171354826
H -4.272733874 -3.9170451485 -0.5527312095
C -2.804317918 -2.9598763658 0.7026954931
C -1.7288117069 -2.0785475521 0.6361477502
H -1.1683424787 -1.796892656 1.5170993636
C -1.7064631252 -3.9916541028 3.3415248323
H -1.3053176809 -2.9818768195 3.49892767
C -0.6353855095 -4.79204353 2.5836058305
H 0.3000200224 -4.8082838848 3.149229805
H -0.4121470628 -4.3723110803 1.5991388402
H -0.9396992907 -5.8283104827 2.4339293941
C -1.9518796313 -4.5973752056 4.7344043905
H -1.0260676549 -4.6127155341 5.3149144373
H -2.3020674353 -5.6282001971 4.6615054215
H -2.6898918733 -4.0359076802 5.3103441808
C -4.3921708722 -5.2200245599 2.0219843013
H -5.1538571729 -4.8487023231 1.3229843169
C -5.1513167213 -5.7450693127 3.2543937154
H -5.8303221212 -6.5500785903 2.9638646673
H -5.7511903599 -4.9720174765 3.73677864
H -4.471003766 -6.1534972192 4.0033797283
C -3.6345430354 -6.3613160325 1.3242150558
H -4.330869719 -7.13050311 0.9820140547
H -2.9354015797 -6.8448196379 2.0081163614
H -3.0626937948 -6.0250073827 0.4553779941
C -4.35855017 -2.3576414165 3.258532879
H -4.6498077483 -2.7962197262 4.2202370239
C -5.6456549181 -2.0325852762 2.4836001023
H -6.2537589729 -1.3096389786 3.0325340775
H -6.264773127 -2.9150454247 2.3156629398
H -5.422828725 -1.5894896124 1.5081216697
C -3.5655563718 -1.0761534841 3.5558891398
H -4.1943566245 -0.3490040164 4.0756101396
H -3.2198050358 -0.5951939243 2.6352520124
H -2.6954335862 -1.2612893204 4.1885649613
C 0.9041141842 -2.2166173182 -2.9416839592
H 1.1709651964 -2.3658102994 -3.9912727446
H 0.2572852722 -3.049557434 -2.6560363559
C 2.1279370496 -2.2567319162 -2.058267426
C 3.2424732005 -3.0193821673 -2.3504311286
H 3.3020924285 -3.5518887387 -3.290798816
C 4.2691841825 -3.1054904136 -1.4139115462
H 5.134435044 -3.7125420345 -1.6480581253
C 4.1952396937 -2.4308143487 -0.190217848
C 3.0541868921 -1.6592443607 -0.0004315947
H 2.8997757933 -1.0851714821 0.9014275272
C 7.1104551031 -3.1468631426 0.4195910221
H 6.8341951235 -4.0241644735 -0.1807683024
C 7.7152805551 -2.1040886721 -0.5337889672
H 8.5520136828 -2.5336359759 -1.0895646306
H 8.1032638017 -1.2460127336 0.0165849911
H 6.9939581087 -1.7272635689 -1.2636571472
C 8.1559849077 -3.6258030474 1.4421403345
H 9.0335469211 -4.023702748 0.9274123283
H 7.7741244732 -4.4147832825 2.0920993057
H 8.4991033182 -2.8080551663 2.0776501326
C 4.7919615253 -3.9872329636 2.3335884172
H 5.5177789024 -4.0858306544 3.1492189944
C 4.7202864941 -5.3375136233 1.6032621413
H 4.366179497 -6.1218600976 2.276624555
H 5.6900189 -5.6555062148 1.2178289821
H 4.0219725475 -5.2985409501 0.7614281613
C 3.4329646798 -3.6394293227 2.9602408916
H 3.0977603036 -4.4471274039 3.616109626
H 2.6593048777 -3.5072009575 2.1976676569
H 3.4697969982 -2.7289388657 3.561146434
C 5.4710921768 -0.9544592064 2.1542277493
H 4.4433415776 -0.8736482047 2.5339520848
C 5.7365327584 0.2853840304 1.2858655658
H 5.5700243814 1.1991216178 1.8625471405
H 5.0910304487 0.328710547 0.4047563465
H 6.7676562099 0.3132804839 0.9331711788
C 6.3965222522 -0.96733083 3.3839542744
H 6.2525930412 -0.0636093474 3.9813389226
H 7.4470700189 -0.9922588202 3.0900229893
H 6.2137444531 -1.82339617 4.0362203826
O 0.844586813 -0.7608579725 2.217425688
O 0.6277143933 0.169078966 1.1780827698
H 0.8862290465 -0.184251739 2.998057363

134
2-2a-2+ 2 2 -4264.7282165 1.173269 0 B3LYP-D3/def2TZVP
Fe -0.3236804752 -0.7182223423 -0.5928997286
N 0.0471171371 1.074357082 -1.3789834408
Si 0.763300679 4.9787973867 -0.0740099948
C 0.2206562097 2.187888696 -0.6433406777
H 0.1546652384 2.0312784611 0.4227093116
Si -3.7265864607 -4.3997319984 2.3712145475
N -0.4868877611 -1.2568503467 -2.5882638646
C 0.4835969224 3.4374046631 -1.1893512155
Si 5.1312393416 -1.309625805 0.9558704343
N -1.7963657072 -2.0608001925 -0.4754662131
C 0.5204434335 3.4917920306 -2.5888986386
H 0.714373536 4.430258562 -3.0925404973
N 1.4018586009 -1.5784170382 -0.8276980979
C 0.3241464561 2.3513388127 -3.3588262563
H 0.3604628909 2.3956100084 -4.4396230529
C 0.111389472 1.1421084097 -2.722175908
C 2.161045564 5.9575709054 -0.8985277677
H 1.7582150071 6.2057301539 -1.8892799999
C 3.4593237835 5.1656374238 -1.119439227
H 4.1559512321 5.7379197423 -1.7367569531
H 3.2889541512 4.2082930873 -1.6181015354
H 3.9643440233 4.9594271778 -0.1749455282
C 2.4462491561 7.2907217679 -0.1855222429
H 3.1444150498 7.8942916539 -0.7700886746
H 2.9028929454 7.1280543184 0.7924587969
H 1.5443069486 7.8872038667 -0.0368385614
C 1.0902961343 4.3199505912 1.6795338474
H 0.2263878386 3.6740265975 1.8871106153
C 1.0674316834 5.4363371554 2.7387239811
H 1.1513129496 5.0125201694 3.7426629299
H 0.146873733 6.0207002813 2.7075683028
H 1.902158883 6.1266597547 2.6096965107
C 2.357338545 3.4626407962 1.8286838744
H 2.3837694168 2.9832458301 2.811355736
H 3.2567863232 4.0739452539 1.7493809959
H 2.434762286 2.6770438795 1.0737034811
C -0.8613744789 5.9571222278 -0.1042521992
H -0.6860004148 6.8218992703 0.5463645422
C -1.2133294332 6.4896704708 -1.5014714388
H -2.1299774707 7.0831267356 -1.4660685938
H -1.3920627282 5.6731172358 -2.2075273634
H -0.4311130808 7.1280391775 -1.9151113476
C -2.0331385823 5.1563903649 0.4852313095
H -2.9441496827 5.7593718225 0.4971189058
H -1.8439997523 4.8339755615 1.510579963
H -2.2461304136 4.2650426261 -0.1133210048
C 0.0594427834 -0.1706167985 -3.4533665967
H -0.5097164218 -0.0967375978 -4.3820099766
H 1.0805521244 -0.4412545279 -3.7277695101
C -1.9384911159 -1.4926244462 -2.8056576778
H -2.1217903679 -2.0073225734 -3.7521666158
H -2.4239833443 -0.514946118 -2.857135974
C -2.4682631348 -2.2681390404 -1.6309935362
C -3.5520181622 -3.1191886876 -1.6731042852
H -4.0788519625 -3.2810249561 -2.6043378219
C -3.9459608509 -3.7651536096 -0.5022730748
H -4.7950476872 -4.4353259438 -0.5411940352
C -3.2581409538 -3.5724168952 0.6987844883
C -2.1781404653 -2.6931597048 0.6456602452
H -1.5737332284 -2.4713281217 1.5124364069
C -2.0713680145 -4.8161999157 3.2044434147
H -1.6095163751 -3.8363937115 3.3852936144
C -1.0901545071 -5.6261043431 2.3422533232
H -0.1262977123 -5.7206444976 2.8495058759
H -0.9048538968 -5.1628192015 1.3695713807
H -1.455534313 -6.6364077657 2.1567872311
C -2.2626002477 -5.4806559206 4.5793362509
H -1.305278136 -5.5668301466 5.0991738762
H -2.6643524321 -6.4903226706 4.4787004195
H -2.9373756714 -4.9178294211 5.227206983
C -4.8745483814 -5.8481311438 1.9646315985
H -5.6481743708 -5.4072896498 1.3214342402
C -5.5983287228 -6.403720075 3.2042528867
H -6.324281967 -7.1646424082 2.9087345955
H -6.1402048696 -5.6315796536 3.7522745642
H -4.9010525925 -6.8760867674 3.8978028681
C -4.2016110045 -6.9820392041 1.1746105613
H -4.9479501765 -7.695057694 0.8165372998
H -3.5041580542 -7.5385619535 1.8025268584
H -3.6441585349 -6.6241467991 0.305003891
C -4.6498557557 -3.0663368422 3.358205135
H -4.9165993589 -3.5491680411 4.3058682948
C -5.9536018583 -2.640256594 2.6644986125
H -6.4962555377 -1.9141406352 3.2744486921
H -6.6261183887 -3.481101294 2.4881579018
H -5.7543441038 -2.1644321342 1.6994001947
C -3.7859332773 -1.8404258881 3.6906276602
H -4.3516098069 -1.1262667108 4.2941111264
H -3.4741760734 -1.312079049 2.7839240307
H -2.8893803629 -2.1033880457 4.2554908601

C 0.3073196538 -2.5148567866 -2.7312076136
H 0.5242001604 -2.7279815412 -3.7795653022
H -0.2932491996 -3.3379147408 -2.3421660602
C 1.5531489252 -2.3720076689 -1.9067604528
C 2.7690574085 -2.970229321 -2.1664129479
H 2.8842934981 -3.610612442 -3.0310269144
C 3.8404835451 -2.7146728587 -1.3119247024
H 4.7959660464 -3.1743602808 -1.5286316535
C 3.7066248356 -1.8592775824 -0.2145789224
C 2.4369118496 -1.3241014651 -0.0137800835
H 2.2177120748 -0.6576653756 0.8063443451
C 6.7470182713 -1.9355352158 0.1947929405
H 6.5501949604 -2.9926568685 -0.0289064683
C 7.1350493579 -1.2419113659 -1.1220292139
H 7.9715002419 -1.7609883504 -1.5957195517
H 7.4572166322 -0.2148119878 -0.942615708
H 6.317700104 -1.205557249 -1.8468706976
C 7.9254072619 -1.9057758535 1.1853319926
H 8.8087420895 -2.3632227631 0.7339429387
H 7.7107147343 -2.4503022673 2.1053686205
H 8.1956946586 -0.8843407393 1.4581734188
C 4.8109651259 -2.1400470162 2.6321824537
H 5.6480990726 -1.8229728747 3.2652893978
C 4.8606040783 -3.6729966837 2.5220127072
H 4.7461111756 -4.1342560795 3.5059663362
H 5.8024358343 -4.0319225697 2.1044572182
H 4.0507241323 -4.0522675972 1.8916982458
C 3.5144912461 -1.689763084 3.3236213032
H 3.4306400093 -2.1540281503 4.3095400513
H 2.629896228 -1.9907322476 2.7549106462
H 3.4711683029 -0.6084226585 3.4709034798
C 4.9255192841 0.578181036 1.0836779818
H 3.9861166934 0.710956234 1.6353226469
C 4.7739880091 1.3104906825 -0.2587305256
H 4.6089498972 2.3779749142 -0.0955364823
H 3.9296778957 0.938614128 -0.8457971764
H 5.6676505334 1.2132321173 -0.8752428815
C 6.0373418548 1.2214813323 1.930158787
H 5.8167745255 2.275892494 2.1140993889
H 6.9999715468 1.1781753966 1.4177102514
H 6.1560699667 0.7384027122 2.9019292574
O 0.0155088861 -1.0698500939 2.0579018132
O -0.2485307618 -0.0772689054 1.0544060234
H -0.0950084849 -0.5514114534 2.8708637738

134
4-2a-2+ 2 4 -4264.7444956 1.1720435 0 B3LYP-D3/def2TZVP
Fe 0.6929932043 -0.8644880104 -0.6781578006
N 0.4848810258 1.0294148752 -1.3032702807
Si -0.6834323452 4.7267810231 0.1930660369
C 0.163412794 2.066862456 -0.5181697134
H 0.1163273409 1.8334948348 0.5367445532
Si -3.7543617592 -3.2189896181 2.3168790475
N 0.4525814003 -1.2508332145 -2.6666247193
C -0.0984505453 3.344058377 -1.0075601219
Si 6.187726098 -2.255795087 0.7633212919
N -1.0872973007 -1.8234814217 -0.5479945321
C 0.0125185288 3.4945988904 -2.3940689651
H -0.1744407422 4.4583435697 -2.8502485527
N 2.4463326877 -1.8001428047 -0.9982785429
C 0.353752395 2.4221948103 -3.2177425237
H 0.4335791978 2.5460995686 -4.289746277
C 0.5817819911 1.1896792493 -2.6402011824
C -0.396473088 6.3791974374 -0.6814536538
H -0.8829753107 6.256024711 -1.6585436889
C 1.0831946389 6.7076964549 -0.9380618287
H 1.1736247793 7.579392388 -1.5903462815
H 1.625954655 5.885022163 -1.4111081783
H 1.6003430865 6.9491015062 -0.0083120053
C -1.1050566458 7.5545586459 0.0156316117
H -0.9905531738 8.4673660132 -0.5734519239
H -0.6810790182 7.7519790046 1.0012160361
H -2.1748909626 7.3814397657 0.1428597625
C 0.2433459859 4.4296769547 1.822631655
H -0.1132122302 3.4466247949 2.1583462171
C -0.1554999813 5.4420492728 2.9109264487
H 0.273522358 5.1567057643 3.8745152089
H -1.2369809451 5.5120650535 3.0424690408
H 0.2164335148 6.4410268334 2.677736391
C 1.7723635378 4.3422974404 1.6946718974
H 2.2184471356 4.0478702301 2.6483619515
H 2.207310642 5.3039280994 1.4211285473
H 2.0920375131 3.6154659021 0.9435626528
C -2.5428280919 4.4100368246 0.4313511975
H -2.8725435363 5.1888139485 1.1291155474
C -3.3317953651 4.5873673692 -0.8752159119
H -4.4026686838 4.4572888051 -0.7009168799
H -3.0352790598 3.8451586993 -1.6230999706
H -3.1930809247 5.5758210499 -1.3151205802
C -2.8572417216 3.0498233484 1.0727779357
H -3.933358698 2.9371993115 1.2268197398
H -2.3772083318 2.9209289449 2.0444027213
H -2.5418749041 2.2212134364 0.4299599759
C 0.9853221934 -0.0541695288 -3.3870837782
H 0.6456828041 -0.0425779326 -4.4246623845
H 2.0741496905 -0.1359517477 -3.3935197172
C -1.0048546753 -1.4432069818 -2.9271647416
H -1.1560799174 -2.023055252 -3.8399868124
H -1.4412197122 -0.4561411368 -3.0922474311
C -1.678810039 -2.0674204063 -1.7363386123
C -2.8741128452 -2.7541350218 -1.8025080804
H -3.3306972897 -2.9619262498 -2.761439701
C -3.4876403649 -3.1475246541 -0.6151302384
H -4.4320530217 -3.6737645903 -0.6692307522
C -2.9109978822 -2.8676873329 0.6273573954
C -1.6826764628 -2.2121250585 0.5910072095
H -1.1290720246 -1.9814963632 1.488635347
C -2.3589741435 -3.6454582632 3.5310705692
H -1.7806507322 -2.7159721838 3.6148742671
C -1.3958267033 -4.7434552343 3.053319661
H -0.5674997216 -4.8574407033 3.7577559583
H -0.9635849674 -4.5279157169 2.0731060322
H -1.8933133551 -5.7111940726 2.9843397986
C -2.8982541287 -3.9677930853 4.93537076
H -2.07670926 -4.0624588086 5.6496047491
H -3.4391950416 -4.9152518444 4.9406913183
H -3.5735062145 -3.1975831216 5.3132377249
C -5.0666944457 -4.5485022444 2.0165184378
H -5.648950302 -4.1663308622 1.1672227397
C -6.0528786827 -4.7008926913 3.1891056889
H -6.8474102442 -5.4023314518 2.9243396719
H -6.5291608242 -3.7575114916 3.4612423784
H -5.5607826921 -5.0932813989 4.0802368469
C -4.4921595579 -5.9149683853 1.6080239026
H -5.2918052179 -6.5832577596 1.2800489648
H -3.9950914159 -6.4011123992 2.4489467553
H -3.7657765362 -5.8456819056 0.7940450399
C -4.56945307 -1.5672938751 2.7923075264
H -5.040578514 -1.7535253637 3.764932944
C -5.6765779112 -1.1693011507 1.804125715
H -6.1724413905 -0.2508546912 2.1279425045
H -6.4459146375 -1.9366810186 1.7101137963
H -5.2702138044 -0.9797389945 0.8057200663
C -3.5679372778 -0.4166935467 2.9740854599
H -4.0831282922 0.4925445416 3.2940252358
H -3.0583957245 -0.1767964559 2.0354746866
H -2.806501121 -0.6381946633 3.7242805185
C 1.2323321727 -2.488345715 -2.9549911062
H 1.4103604528 -2.5937851454 -4.0275192441
H 0.6214732762 -3.3349708951 -2.6340280426
C 2.5119022598 -2.474642474 -2.1660017924
C 3.671186189 -3.1199364541 -2.5431681506
H 3.7163738267 -3.653730187 -3.4835558316
C 4.773708198 -3.0694942796 -1.6924845877
H 5.6806115744 -3.5794710355 -1.9902593639
C 4.7293066214 -2.3665350697 -0.4843559507
C 3.5192974602 -1.7417333838 -0.1946070061
H 3.3735319551 -1.1663241661 0.7066574123
C 7.7496964638 -2.7841955231 -0.1663858515
H 7.4816412227 -3.7500208284 -0.61560199
C 8.1604243407 -1.8378207471 -1.3065309157
H 8.9532211013 -2.2870102425 -1.9093064492
H 8.5512315928 -0.8968987353 -0.9160365096
H 7.3341598728 -1.594412406 -1.979783965
C 8.9402983058 -3.0536942794 0.7716559212
H 9.7819737699 -3.4603036974 0.2063824777
H 8.6983579168 -3.7711041204 1.5569983852
H 9.2873603197 -2.1383247039 1.2530559365
C 5.7793279253 -3.4976711994 2.1418011872
H 6.617829047 -3.4237118339 2.8444011832
C 5.7297350935 -4.9421440176 1.6192079816
H 5.5513968092 -5.6431195315 2.4381623852
H 6.6596950778 -5.2417817236 1.1339809461
H 4.9179172 -5.0773740723 0.8980191936
C 4.4962761426 -3.1581446186 2.9163770498
H 4.337345929 -3.876026985 3.7249029038
H 3.6131571024 -3.2061949221 2.2712456049
H 4.5351856097 -2.1651348125 3.3694200695
C 6.1486811609 -0.4818261223 1.442259123
H 5.1996732854 -0.4238046784 1.9924628392
C 6.1269530642 0.6250479634 0.3767824283
H 5.9848593427 1.6030707416 0.8440305065
H 5.3240066714 0.4892244815 -0.3525580796
H 7.0649765898 0.6673244074 -0.1772174657
C 7.2721522361 -0.2356947 2.463940305
H 7.1397929873 0.7307166086 2.9564962475
H 8.2490955568 -0.217245423 1.9784963039
H 7.3032628228 -0.9990621677 3.2435893148
O 1.1118808628 -1.5743795027 1.8334037533
O 0.9365389757 -0.4032145692 1.0144499538
H 1.4726799467 -1.1876818062 2.647241115

134
4-3-2+ 2 4 -4261.9462653 1.1712959 0 B3LYP-D3/def2SVP
Fe -0.1167155449 -1.6779947147 -0.8733711417
N 0.0202810378 0.2050083835 -1.60627886
Si 0.1543842244 4.1850247432 -0.2230623194
C 0.0419707829 1.3015096978 -0.8177493153
H -0.1033547408 1.1030685874 0.2451192053
Si -5.4642841935 -0.3602481524 0.9588949684
N -0.1425208472 -2.1615981272 -2.9274822139
C 0.2411111581 2.5932555935 -1.3158443375
Si 5.4919119187 -1.1005057445 0.8017908821
N -2.0678185685 -1.544409311 -1.1458694748
C 0.470723767 2.6868644577 -2.7039124285
H 0.6835053419 3.6556024567 -3.1616841614
N 1.8419049286 -1.8317042595 -1.1479484752
C 0.4148596834 1.55821133 -3.5206324938
H 0.5556944984 1.6341871158 -4.6013320489
C 0.1630010326 0.3178030447 -2.9377422482
C 1.80933733125 5.0918764697 -0.4834190423
H 1.773608739 5.3973817222 -1.5468343406
C 3.0654443147 4.2244791821 -0.2998893059
H 3.969038775 4.784216372 -0.5938496259
H 3.0365767616 3.3052923781 -0.9084650683
H 3.2012903299 3.921900845 0.7484146582
C 1.8852887042 6.3772495372 0.3622913894
H 2.7813655969 6.9638793507 0.100912508
H 1.9527028912 6.1466089904 1.4380542447
H 1.0116842192 7.0323819331 0.2157139011
C -0.2461405699 3.6118523179 1.5523557726
H -1.0467612047 2.8596269917 1.4189565448
C -0.8351539781 4.733262654 2.4295768985
H -1.0939420144 4.3448430834 3.428801048
H -1.7514974303 5.1667776605 2.0011325927
H -0.117292326 5.5553340521 2.5788088259
C 0.930838449 2.9245623191 2.2663039817
H 0.603049266 2.4706764847 3.2165783586
H 1.7230139819 3.64881092 2.5127459887
H 1.3996423258 2.13045881 1.6636172346
C -1.2933063336 5.2313140161 -0.9004671116
H -1.3753641843 6.0506473285 -0.161735619
C -1.0516737505 5.8805305838 -2.272952883
H -1.8784980282 6.5648580893 -2.5255314348
H -1.0137481491 5.1302940424 -3.0810849262
H -0.1221853401 6.4691010625 -2.3105466319
C -2.6182393013 4.4519062878 -0.8848701211
H -3.4612945626 5.0959202704 -1.1854381514

H -2.8559862269 4.0461488783 0.1081543909
H -2.5945825112 3.6027108377 -1.5902460264
C -0.0138216732 -0.9296049811 -3.7606386049
H -0.9197482621 -0.8139349353 -4.3765765368
H 0.8244784884 -1.0390049426 -4.4655816495
C -1.4595471962 -2.8308007114 -3.1067861435
H -1.3728987592 -3.8473497354 -2.6958830524
H -1.7373870358 -2.9121591853 -4.1694727975
C -2.504897945 -2.1007618125 -2.3007079805
C -3.8596334318 -2.0609547811 -2.6102234364
H -4.2155297101 -2.4898616412 -3.5495331101
C -4.7518466197 -1.4968582065 -1.6894094238
H -5.8162481085 -1.4840624879 -1.9350927549
C -4.3045813805 -0.9817458072 -0.4596536542
C -2.9184281341 -1.0155791346 -0.2530512834
H -2.4540392227 -0.6362466902 0.6591404743
C -4.8833819102 -1.3198598484 2.5045909725
H -3.8807215527 -0.9012115479 2.7189606967
C -4.7091995745 -2.8360797531 2.3063100576
H -4.2924576752 -3.299081494 3.2164558478
H -4.0279419175 -3.0780856364 1.4730868056
H -5.667399428 -3.3363555601 2.1013556753
C -5.7757348397 -1.0141188578 3.7228297654
H -5.3480421328 -1.4521800522 4.6397321943
H -6.7825337425 -1.4442129257 3.5998299261
H -5.8948304407 0.0668941986 3.9002564683
C -7.2557197843 -0.6698093206 0.3947679194
H -7.2900694849 -0.2844744032 -0.643539946
C -8.2813918554 0.1460905344 1.2072483858
H -9.2985773086 -0.0212300453 0.8166568356
H -8.0910806892 1.229346291 1.1651764276
H -8.2903539624 -0.1504037797 2.2679128087
C -7.647079028 -2.1593840902 0.3637179404
H -8.6285050835 -2.2937243999 -0.1197964369
H -7.734270544 -2.5672903435 1.3830707594
H -6.9228051904 -2.7903819056 -0.1777844892
C -5.148867192 1.5080027908 1.1830533633
H -5.8768106088 1.8030357278 1.9615702042
C -5.4735464965 2.3016193679 -0.0945945196
H -5.3811838695 3.3863164631 0.0779078714
H -6.4965938771 2.1161641824 -0.4566853551
H -4.7790382607 2.0452150892 -0.913431677
C -3.7473411739 1.8514571592 1.7116996859
H -3.6653756606 2.9276491809 1.9362759839
H -2.9690048391 1.6264037205 0.9619853441
H -3.4935476765 1.3120713279 2.6372813594
C 1.0353067384 -3.0584460313 -3.0746133591
H 1.3014750587 -3.2135307022 -4.1321098921
H 0.7704679043 -4.0324992303 -2.6386947516
C 2.1831796969 -2.4909143471 -2.2801205016
C 3.5249266061 -2.6720726781 -2.596586891
H 3.8018145716 -3.1853888234 -3.5199251286
C 4.4977945427 -2.2063506826 -1.7068703642
H 5.5506420773 -2.3539657303 -1.9592612462
C 4.1445143628 -1.5679945491 -0.5021455976
C 2.7728214041 -1.3884318415 -0.2844187077
H 2.3803834102 -0.8874236811 0.6028541028
C 6.9296954704 -0.3336027532 -0.1865667978
H 7.3341691823 -1.1822281111 -0.7710306845
C 6.5158849831 0.7589358318 -1.1888666826
H 7.3817608789 1.0774610771 -1.7927677187
H 6.1308512109 1.6560515742 -0.6800875066
H 5.7359201721 0.4178707402 -1.8898173035
C 8.060355582 0.1568487545 0.7383943952
H 8.9458870783 0.4460843365 0.14909326
H 8.3832479046 -0.6123722882 1.4580457995
H 7.751932079 1.0447133966 1.3135394703
C 5.9956228098 -2.75893528 1.6002106042
H 6.754638062 -2.4744614775 2.3530503131
C 6.654751717 -3.7469469478 0.6242763065
H 6.9946439331 -4.6504133917 1.1568094249
H 7.5343331079 -3.3212752578 0.1165925061
H 5.9462217098 -4.0874649432 -0.1510308226
C 4.8169593267 -3.416485341 2.3401263215
H 5.1382872137 -4.3425522339 2.8446492839
H 4.0092791937 -3.6958358344 1.640103618
H 4.3804120236 -2.7615811835 3.1104238436
C 4.668769546 0.0200470883 2.103537995
H 3.7311655252 -0.5055000479 2.372782138
C 4.2924799243 1.409905613 1.5677327311
H 3.7190511089 1.9754222623 2.319318181
H 3.6833913286 1.3671643354 0.64964916
H 5.187465444 2.0069694316 1.3337227492
C 5.4992037628 0.1352646435 3.3969961172
H 4.9578105541 0.7310460264 4.150672958
H 6.4624268121 0.6378090001 3.218957234
H 5.7150479124 -0.8444974644 3.8494174639
O -0.2520648842 -3.4236249673 -0.5613680532
O -0.0810964524 -1.1755567001 0.6731567082
H -0.275692939 -3.6354037811 0.3871059157

134
2-3-2+ 2 2 -4261.9262249 1.1703612 0 B3LYP-D3/def2SVP
Fe -0.1155140889 -1.9224915902 -0.5872857272
N 0.0101889862 -0.0874288363 -1.4463013336
Si 0.1005501608 3.9780585352 -0.3388128097
C 0.0188349345 1.0602751722 -0.7346615737
H -0.1281932315 0.9319347901 0.3383273507
Si -5.4755061611 -0.5169155041 1.1688375465
N -0.1262815219 -2.5339312051 -2.5848437012
C 0.209412945 2.3167927269 -1.3198105465
Si 5.4724410302 -1.1642389783 1.1124084777
N -2.0617769537 -1.8129305158 -0.8437200353
C 0.446415431 2.3176788709 -2.709644665
H 0.6536045192 3.2547060729 -3.2314956368
N 1.8428441742 -2.0611370074 -0.808895649
C 0.4047800386 1.1352132564 -3.4475713933
H 0.5511454346 1.1379917262 -4.5301751934
C 0.1592812761 -0.0635424287 -2.781504756
C 1.7465738513 4.8840678016 -0.6532534048
H 1.7135255642 5.1145780508 -1.7354905796
C 3.011240722 4.0460938091 -0.4037041438
H 3.9101100485 4.5941655324 -0.7321033317
H 2.9957629799 3.0862432539 -0.9464675835
H 3.1449008701 3.8193328907 0.6638426508
C 1.8031465318 6.2257603703 0.1015318517
H 2.694167149 6.8031881036 -0.1952306316
H 1.8666532395 6.0710766475 1.1910439516
H 0.9231678813 6.858996204 -0.0947014817
C -0.302604662 3.5232502193 1.4700335929
H -1.0943041853 2.7548982232 1.3847033181
C -0.9080924268 4.6950711312 2.2663571664
H -1.168199093 4.3725414723 3.2884309176
H -1.8266173682 5.0885135199 1.80517261
H -0.19980504 5.5331847717 2.3628389319
C 0.8778139322 2.8989273603 2.2342040087
H 0.5499448338 2.5067573065 3.2115508351
H 1.6608770888 3.6468849497 2.4345443715
H 1.3580642137 2.0710458328 1.6887159854
C -1.3555027166 4.9590548883 -1.0918896178
H -1.4511261211 5.825611606 -0.4109592597
C -1.1137926315 5.5163025475 -2.5041902125
H -1.9473705854 6.1714141648 -2.8070502318
H -1.0618284932 4.7133002413 -3.2590749066
H -0.1913169116 6.1125391753 -2.5770410313
C -2.6714112357 4.1667178569 -1.0295711389
H -3.520317025 4.77895659 -1.3764902238
H -2.9096079374 3.8258982263 -0.0125196113
H -2.6341731987 3.2723677596 -1.6761126812
C -0.0064157372 -1.3676901878 -3.5112127403
H -0.9133799133 -1.3072587347 -4.1332889363
H 0.8329958752 -1.5251197655 -4.2054714543
C -1.439666054 -3.2248086578 -2.7157087722
H -1.3442041569 -4.21121673 -2.2388289946
H -1.7177788743 -3.378746376 -3.7700463203
C -2.4923539002 -2.4501160827 -1.9587704378
C -3.8468330609 -2.4442854512 -2.2720934375
H -4.1978855409 -2.9389263989 -3.1803841481
C -4.74578361 -1.8271017296 -1.3926619417
H -5.8098259616 -1.8414094065 -1.6397382835
C -4.3056300598 -1.2239399954 -0.2006884238
C -2.919728857 -1.2304548855 0.0078652304
H -2.4621484296 -0.7801791417 0.8916676947
C -4.8850237096 -1.3560524427 2.7796930205
H -3.8872163141 -0.9118007912 2.9633206956
C -4.6938373156 -2.8805828454 2.6911274927
H -4.2719593636 -3.2721022204 3.6319391768
H -4.0100187445 -3.1745118363 1.8769044912
H -5.6463929411 -3.4049292128 2.5235145086
C -5.7815535472 -0.9734230573 3.9728643933
H -5.3496239001 -1.3394463776 4.9188991357
H -6.7833536103 -1.4225877973 3.8812608997
H -5.9129438294 0.1161444569 4.0719529643
C -7.262032652 -0.8883472566 0.6271367044
H -7.2996519501 -0.577830853 -0.4358323949
C -8.2989882987 -0.0298427237 1.3791494165
H -9.3134106089 -0.2369820816 1.0006673321
H -8.121988243 1.0500020012 1.2606792149
H -8.3059508974 -0.2506600041 2.4581178004
C -7.6348060099 -2.3811372509 0.7011711227
H -8.6136907156 -2.5615847404 0.2276229687
H -7.7186021568 -2.7170435279 1.7467365276
H -6.901839916 -3.0396570791 0.2061240529
C -5.1833972728 1.3663501961 1.2582653207
H -5.9162603707 1.7080260753 2.0127033928
C -5.5149567048 2.0617221135 -0.0736902796
H -5.4353324117 3.1570512787 0.0201187166
H -6.5352189195 1.8387959869 -0.4222691916
H -4.816157565 1.7549735312 -0.8712729228
C -3.7869433634 1.7632366334 1.762122172
H -3.7164970425 2.8540288342 1.9055417377
H -3.0044497947 1.4908458566 1.0327544862
H -3.5299383327 1.2973592108 2.7259440164
C 1.0650272058 -3.4247285597 -2.6582115278
H 1.3448149748 -3.6458181982 -3.7001158424
H 0.8063757637 -4.3713700942 -2.1625090175
C 2.1999761709 -2.7917861411 -1.8914853161
C 3.5458057928 -2.9817841789 -2.1849479094
H 3.8354751133 -3.5534970818 -3.0692187388
C 4.5072601461 -2.448192818 -1.3212555391
H 5.5633171461 -2.6037426632 -1.5546515132
C 4.1388284837 -1.7312442031 -0.1660708722
C 2.7640875733 -1.5494017847 0.026929846
H 2.3624874346 -0.9868958131 0.8730764984
C 6.9163408689 -0.4643926578 0.0840081236
H 7.3288240961 -1.3512122776 -0.4343950535
C 6.508188169 0.5517903895 -0.9978015075
H 7.3784529664 0.8290333963 -1.6155896749
H 6.1161980075 1.4818090579 -0.5581900179
H 5.735357875 0.158612859 -1.6790735132
C 8.0370434584 0.095456046 0.9812425239
H 8.9268728921 0.3451834339 0.3804644537
H 8.3559630803 -0.618996046 1.7569286046
H 7.7206236506 1.0210405485 1.4885671123
C 5.9751794079 -2.7586759218 2.0324204557
H 6.7254570749 -2.4175756735 2.7703175804
C 6.6480308893 -3.8105734553 1.1358119091
H 6.9850753398 -4.6727989218 1.7344223171
H 7.5316716361 -3.4182235016 0.6087422261
H 5.9491561627 -4.2077336663 0.3789007511
C 4.7918792705 -3.3673502518 2.8059868416
H 5.1119613161 -4.2532154829 3.3788460101
H 3.9925998767 -3.700271103 2.1198515617
H 4.3446565526 -2.6610701651 3.5229441747
C 4.6336712665 0.0440166812 2.3225320095
H 3.6945837296 -0.463317269 2.6201828424
C 4.2598899732 1.3906213611 1.6842581927
H 3.6772729936 2.0070324868 2.3871310847
H 3.6607313369 1.2799630432 0.7653329732
H 5.1560755545 1.9718437064 1.4173140862
C 5.4513773884 0.2542959763 3.6121516482
H 4.9017553871 0.9017455766 4.3155723709
H 6.4154501012 0.7449274068 3.4074437403
H 5.6644338246 -0.6897046627 4.1362376733
O -0.2348718715 -3.6298271536 -0.1455187198
O -0.0929210849 -1.2943375715 0.9693925812
H -0.2604224605 -3.764261177 0.817512254

134
4-3a-2+ 2 4 -4261.9453569 1.1712505 0 B3LYP-D3/def2SVP
Fe 0.0802663331 -2.0284178461 -0.4501478528
N 0.0422282099 -0.1969795063 -1.3770505267
Si -0.2066039111 3.8742916713 -0.3860888181
C -0.0313034309 0.9766194874 -0.7207430443
H -0.0743695098 0.8852080605 0.3649429023
Si -5.4977309865 -0.7491064589 0.8440579443
N 0.1427465677 -2.7253000689 -2.4026849459
C -0.0439681392 2.2184137068 -1.3669434277
Si 5.4856081436 -0.5205734238 1.1263235968
N -1.8750256222 -2.0996505896 -0.7946427343
C 0.0232380412 2.17444379 -2.7738387126
H 0.0257506037 3.1001630039 -3.3545040828
N 2.0519531336 -2.0153334885 -0.7161472867
C 0.0872691891 0.9571088473 -3.4552719787
H 0.1334181328 0.9245461082 -4.5463170863
C 0.0944527726 -0.2254075707 -2.7168679227
C 0.8296156499 5.160226543 -1.332325605
H 0.3923420146 5.148599159 -2.3498321435
C 2.3190731694 4.8005001107 -1.4655662508
H 2.8264930517 5.4890664825 -2.1611304917
H 2.4788293873 3.7753372918 -1.838488451
H 2.8375453286 4.8843577333 -0.4979089829
C 0.6460561685 6.5879367813 -0.7840295902
H 1.1601641089 7.3190980504 -1.4292747008
H 1.0747997815 6.6918600806 0.2254055269
H -0.4122921608 6.8877032417 -0.7296965475
C 0.2905942348 3.4889392635 1.4152773464
H -0.394282107 2.6666771831 1.7025796388
C 0.0048730041 4.6618326648 2.3719035368
H 0.1875129066 4.3647404883 3.4180551847
H -1.0361804466 5.0151599337 2.3085399152
H 0.6596489606 5.5221120419 2.1622318534
C 1.7275506687 2.9719934685 1.5865511868
H 1.890156097 2.5911530416 2.6092013827
H 2.465879067 3.7713437366 1.42210953
H 1.9740031589 2.160447518 0.8840976906
C -2.0556879823 4.3399033486 -0.4445707364
H -2.1177311364 5.3062138984 0.0890931592
C -2.5723791255 4.5574630508 -1.876333364
H -3.6281699838 4.8754551122 -1.8689014455
H -2.5230114939 3.6278730982 -2.4703914867
H -2.0053021097 5.331978061 -2.4159182675
C -2.9273037233 3.3261537616 0.3110867304
H -3.9876072126 3.6272999328 0.2946670865
H -2.6403389508 3.2201281406 1.3686439281
H -2.8670525133 2.3273615483 -0.1552893131
C 0.1677086448 -1.5813118147 -3.3770126994
H -0.6703495728 -1.6921033668 -4.0820448631
H 1.0892081008 -1.6445049677 -3.9756026816
C -1.0869354608 -3.5648835468 -2.5303519142
H -0.8932968615 -4.513841146 -2.0078290121
H -1.3049681071 -3.7954384806 -3.5848162681
C -2.2297911827 -2.8729974772 -1.8434099129
C -3.5728094951 -3.0413954155 -2.1614452405
H -3.8587272585 -3.6636045918 -3.0122557432
C -4.5355747454 -2.4201234075 -1.3602824459
H -5.590643512 -2.579058955 -1.5940987751
C -4.1689842175 -1.6015890978 -0.2749053952
C -2.7930560506 -1.4685603508 -0.0439407057
H -2.3860920565 -0.858470331 0.7631814816
C -4.5376203286 0.4100276314 2.0227498677
H -3.7608790038 0.8697344021 1.3831589857
C -3.8273541389 -0.3171082456 3.1805263624
H -3.1679395849 0.3762517176 3.7295736426
H -3.2118442262 -1.1715945885 2.8525431902
H -4.5542353982 -0.7103301259 3.9079916437
C -5.4021512886 1.5647454944 2.5644456581
H -4.8025583598 2.2222725047 3.2161810547
H -6.2465770066 1.1950192453 3.1680264933
H -5.8154020964 2.1930705426 1.761308917
C -6.4584518735 -2.1743992287 1.6638017752
H -6.9937893939 -2.6451259996 0.816926443
C -7.5199817375 -1.6334415913 2.6407734609
H -8.1611214574 -2.450315221 3.0108755422
H -8.1807201242 -0.8844488172 2.1752113073
H -7.0536584168 -1.1642005447 3.5224400112
C -5.5915533874 -3.2647829397 2.3164195553
H -6.2199927353 -4.1058377108 2.6531375431
H -5.0571924041 -2.8898563467 3.2022964728
H -4.8398758283 -3.6763017828 1.6223336349
C -6.6389706927 0.2702662646 -0.299102268
H -7.1981506171 0.9066788249 0.4117444074
C -7.680294391 -0.5437783819 -1.0854518355
H -8.3623347684 0.1302683104 -1.6297332346
H -8.301741082 -1.1794099589 -0.4368617248
H -7.2141222903 -1.1917433093 -1.8470488111
C -5.8409988641 1.1995325219 -1.2295760687
H -6.5141033236 1.8639427316 -1.7962609295
H -5.2585742526 0.6225148353 -1.9691886053
H -5.1353946998 1.8392661685 -0.6798398512
C 1.3948078858 -3.5398716472 -2.4549847728
H 1.6695409158 -3.7829815001 -3.4933832498
H 1.1978487534 -4.4833223393 -1.9241420388
C 2.482880209 -2.8028497225 -1.7276029279
C 3.8428428618 -2.9347983595 -1.9774134575
H 4.1883048404 -3.5633594989 -2.8010355562
C 4.7482451609 -2.2687992777 -1.1433240583
H 5.8174671577 -2.3977020639 -1.325184602
C 4.3042860045 -1.4444027011 -0.0948502624
C 2.9131123074 -1.3407619288 0.0612637038
H 2.4519828792 -0.7161276944 0.828235898
C 7.2679026021 -0.8126907288 0.5243267049
H 7.3209760223 -1.9013845286 0.3268685621
C 7.6134852615 -0.0726856937 -0.7813844989
H 8.5949852881 -0.3966582232 -1.1645648582
H 7.6782374005 1.0142685065 -0.6153584296
H 6.8754309476 -0.2355780933 -1.584550306
C 8.3135218845 -0.5074216869 1.6162810142
H 9.3265038995 -0.7460805791 1.2527285132
H 8.1515526543 -1.0921288091 2.5344718425
H 8.3132845796 0.5579641452 1.8949278065
C 5.2157172254 -1.3310708547 2.8330323482
H 5.9164907622 -0.7880422338 3.494807948
C 5.6199344286 -2.8165321996 2.8291070628
H 5.5333161217 -3.2506024164 3.8388494494
H 6.6577222588 -2.9728521309 2.496994564
H 4.9658495232 -3.4085781319 2.1652052221
C 3.803037056 -1.1506023193 3.4141338952
H 3.7576388781 -1.5328492214 4.4473212951
H 3.051872493 -1.7207316346 2.8387317593
H 3.4823055407 -0.0968849724 3.4430748902
C 4.8917255438 1.2902304509 1.1264283601
H 3.9186363179 1.249956165 1.6490806494
C 4.6394677008 1.8793722498 -0.2720181433
H 4.2480460839 2.9056113807 -0.1953665791
H 3.9041623505 1.2941502455 -0.8499561548
H 5.5616131847 1.9301034843 -0.8699145571
C 5.8143009335 2.201144373 1.957450348
H 5.3670298483 3.2022428064 2.0763649449
H 6.7907983714 2.3404619664 1.4669153343
H 6.0025525911 1.8045365208 2.9679670408
O 0.101635223 -3.5559734861 0.1380627467
O 0.0364912889 -1.1951445974 1.1102930627
H 0.0412918653 -1.8104340628 1.8647483772

134
2-3a-2+ 2 2 -4261.9280743 1.1703885 0 B3LYP-D3/def2SVP
Fe 0.0769647166 -1.9842816901 -0.476753422
N 0.055560375 -0.1763577002 -1.4083654002
Si -0.2167195373 3.8904587429 -0.4096211563
C -0.0305179472 0.9959296449 -0.7506156976
H -0.0776800731 0.9039954487 0.3347299211
Si -5.4843777823 -0.7408116192 0.8687149402
N 0.1484174004 -2.6973931165 -2.4290692109
C -0.0498597731 2.2378901461 -1.3953824065
Si 5.481575349 -0.5626209283 1.1390304925
N -1.8743738365 -2.0855495178 -0.8000113804
C 0.0173875288 2.1968065553 -2.8023106811
H 0.0122382904 3.1233649367 -3.3815969396
N 2.0464619423 -2.0520671909 -0.7010460328
C 0.0917402538 0.9809109566 -3.4849492483
H 0.1391319242 0.9496135452 -4.5759577137
C 0.1115258006 -0.2028250369 -2.7486545086
C 0.8382236515 5.1732983662 -1.339298997
H 0.4056340903 5.1749483448 -2.3588453281
C 2.3245828064 4.7994881737 -1.4694384589
H 2.8421519222 5.4905429828 -2.1549838227
H 2.4752696362 3.7771129992 -1.8536197592
H 2.8398036197 4.8668135209 -0.4987782963
C 0.6667148229 6.59773223 -0.7787393701
H 1.1880644247 7.3299938226 -1.4168778672
H 1.0953689434 6.6888117363 0.2320042895
H -0.3890081943 6.9064591827 -0.7227481988
C 0.2600998207 3.494991563 1.3954116206
H -0.4245577937 2.6681136428 1.6699888171
C -0.0423915655 4.6605083479 2.3559879085
H 0.1319098105 4.3583171975 3.4020995424
H -1.0850457749 5.0074120984 2.2844699277
H 0.608708541 5.526261649 2.1579066621
C 1.6979193806 2.9840169033 1.580459574
H 1.85075273 2.5986394944 2.6028943243
H 2.4335397974 3.7885009739 1.4289838521
H 1.9570910444 2.1775099619 0.8768298534
C -2.062801288 4.3649911907 -0.4877924079
H -2.1277383287 5.3254768832 0.0559910023
C -2.5599010095 4.6015527528 -1.9235128726
H -3.6142310668 4.9243860992 -1.9256761486
H -2.5075783242 3.6787264811 -2.5277515462
H -1.9826166107 5.3796588279 -2.4467934544
C -2.9493630782 3.3467455789 0.2446351025
H -4.0077183487 3.6541793576 0.2184462681
H -2.6760966552 3.2269265015 1.304402956
H -2.8891161711 2.3529756121 -0.232520507
C 0.2242466043 -1.5568661114 -3.4046289958
H -0.5657684196 -1.6730760672 -4.1622295687
H 1.1828852656 -1.6158930247 -3.942401111
C -1.0976816264 -3.5101760173 -2.5757558163
H -0.9206165985 -4.4740556984 -2.0760585205
H -1.3180504562 -3.7125631556 -3.6353086721
C -2.2336185131 -2.8215111432 -1.8737501711
C -3.5770075118 -2.9633903361 -2.2038878109
H -3.8656395328 -3.5553569519 -3.075092091
C -4.5373275799 -2.3550485554 -1.3900107832
H -5.5925568202 -2.4913357602 -1.6367709007
C -4.167198215 -1.576357981 -0.2767464583
C -2.7913067424 -1.4674056789 -0.0359027673
H -2.3825930343 -0.8840452898 0.7895030229
C -4.5133814401 0.4373379805 2.021607092
H -3.7451788072 0.8907922418 1.3673464647
C -3.7883677439 -0.2708481122 3.1821635206
H -3.1273523805 0.4332075332 3.7154664565
H -3.1716791856 -1.1268267881 2.8607541198
H -4.5062563645 -0.6579048834 3.9217317104
C -5.3755394349 1.5960337869 2.5585881144
H -4.7717557923 2.2611854072 3.1986634388
H -6.2142070169 1.2309400377 3.1730800503
H -5.7964259004 2.2155604637 1.7526867812
C -6.4033703726 -2.1765669528 1.7176765167
H -6.9497161202 -2.6592723329 0.8846783167
C -7.4507886989 -1.6463496137 2.715544674
H -8.0708150361 -2.4706902425 3.1046273557
H -8.1333387298 -0.91029301 2.2610076876
H -6.9722680139 -1.1645572902 3.5838178649
C -5.5058349028 -3.2505027584 2.3557345907
H -6.1136423234 -4.1003681257 2.7078572959
H -4.9598456985 -2.8640984037 3.2294999873
H -4.7620759207 -3.6524314698 1.6476033725
C -6.6692246209 0.2580468736 -0.2475355216
H -7.2062525286 0.900788733 0.474451832
C -7.7352111304 -0.568948189 -0.9861693452
H -8.4361060003 0.0961816743 -1.517301727
H -8.3334296314 -1.1948649693 -0.3070920171
H -7.2957542392 -1.2295435458 -1.7526966119
C -5.9083334207 1.1804066898 -1.2155705028
H -6.6034276524 1.838853676 -1.7622797524
H -5.3530042817 0.5981536151 -1.9716235294

H -5.1832112197 1.8260350625 -0.6983486081
C 1.3796018436 -3.5461378822 -2.46139043
H 1.6645461832 -3.7941720389 -3.4957946565
H 1.1481195099 -4.4841168792 -1.9361034442
C 2.4746764567 -2.8382092083 -1.7150886479
C 3.8345393364 -2.986602091 -1.9571896788
H 4.1767948177 -3.6160091286 -2.7814745561
C 4.7439215296 -2.3312221593 -1.1187261178
H 5.812594716 -2.4687454132 -1.2979170458
C 4.3036209097 -1.5025806384 -0.0720784618
C 2.9134026036 -1.3899246627 0.0814393278
H 2.4570348347 -0.7623257369 0.8486577755
C 7.263445745 -0.8366192949 0.528279876
H 7.3279803235 -1.9264746272 0.3405515122
C 7.5914866742 -0.1053850698 -0.7867854472
H 8.5734437553 -0.4226823849 -1.1743381605
H 7.6461164825 0.983736203 -0.6314627144
H 6.8490806215 -0.2838553627 -1.5826069892
C 8.3122884759 -0.5085995417 1.6103917866
H 9.3257247811 -0.7395012881 1.2431434136
H 8.1623887457 -1.0855860482 2.535490922
H 8.3015837183 0.5595511408 1.8781900218
C 5.230139031 -1.3636423124 2.8525721873
H 5.9259623651 -0.8069864675 3.508188579
C 5.6535730545 -2.8437738132 2.8573982465
H 5.5743819952 -3.2722858781 3.8701066645
H 6.6927488856 -2.9887675701 2.5244758414
H 5.0060499081 -3.4485806932 2.1986228082
C 3.8168406811 -1.1974027198 3.4364323625
H 3.7783216927 -1.5755555168 4.4713859388
H 3.0713790932 -1.7790588228 2.8651586098
H 3.4838197283 -0.1473902015 3.4616204575
C 4.8622379857 1.2409554196 1.1290180902
H 3.8884283594 1.1911943867 1.6503657294
C 4.6046887544 1.8201989296 -0.2727603434
H 4.2005119624 2.8419396418 -0.2017683465
H 3.8776447146 1.2230663497 -0.8490268125
H 5.5270347886 1.8792118369 -0.8694827311
C 5.7712961739 2.1683553987 1.956675831
H 5.3107749207 3.1641231685 2.0697491569
H 6.7466967791 2.3179226739 1.4669259161
H 5.9631663599 1.7794304664 2.9694714755
O 0.0717773193 -3.5826459876 0.1229607912
O 0.0378126376 -1.1776712862 1.0840036598
H 0.0378704702 -1.8095930601 1.8256022978

139
2-4-2+ 2 2 -4414.5292441 1.2094905 0 B3LYP-D3/def2SVP
O -0.0778479375 -1.4233624795 0.8846960206
O -0.1858326872 -3.6521087549 -0.3034965001
C -0.1924924108 -3.7219490095 0.9385176292
O -0.140911843 -2.6381317891 1.6749799918
C -0.2357151012 -4.9980873739 1.7069086992
H -1.0175198984 -5.6445626464 1.2827212198
H -0.4166502911 -4.817797558 2.7733568525
H 0.7318168822 -5.5106769583 1.5802363999
Fe -0.0500696068 -1.7732156545 -0.881382327
N 0.0870831406 0.0932606968 -1.3845661787
Si 0.1912443431 3.8842246356 0.406018863
C 0.1087311717 1.1126925741 -0.5044564644
H 0.0267183043 0.8157778164 0.5425553167
Si -5.5292731248 -0.4038802069 0.6844493592
N -0.0157366044 -2.1787738334 -2.8701841193
C 0.2326738014 2.4522385474 -0.8874224567
Si 5.4057871415 -1.2828353041 1.1010157055
N -1.9988923522 -1.6912090428 -1.1869294079
C 0.337087659 2.6827877727 -2.2742118949
H 0.4462991737 3.700822825 -2.6559393747
N 1.9086244879 -1.9757212703 -1.0654195798
C 0.3018307375 1.626268119 -3.1866903564
H 0.3750073791 1.8085552335 -4.2613263245
C 0.1717097651 0.3241883671 -2.7053303879
C 1.3930423823 5.2158664214 -0.2304451537
H 1.004166592 5.4547350828 -1.2395389728
C 2.8437938929 4.7327785757 -0.3973099436
H 3.4477298827 5.4867455427 -0.9286243559
H 2.9172210989 3.7898401651 -0.9639212768
H 3.3245189848 4.5691151342 0.5795977064
C 1.3276953254 6.515614653 0.5933237304
H 1.9411319475 7.3025023276 0.1244505442
H 1.7178170105 6.3679902358 1.6129863517
H 0.3030129789 6.9094634035 0.6820159034
C 0.5649119285 3.0947678714 2.1028706779
H -0.203327129 2.3016706729 2.1955303077
C 0.3386762637 4.0713488632 3.2723273402
H 0.4482096271 3.552923659 4.2394695554
H -0.66434857 4.5255162377 3.2557425549
H 1.0734628732 4.8915916422 3.2611696491
C 1.9411176615 2.4179386406 2.2062536917
H 2.017688325 1.8201220526 3.1303085706
H 2.7527138658 3.160524112 2.2370654094
H 2.152776934 1.7492160299 1.3572363342
C -1.5987429698 4.5453231425 0.3984249181
H -1.5915896997 5.3795879227 1.1239961817
C -2.0218326891 5.1118320306 -0.9669329973
H -3.040913239 5.5303414692 -0.9197564882
H -2.0343286519 4.3278015481 -1.7443040883
H -1.3566933396 5.9182679025 -1.3131188852
C -2.6021158833 3.4943843187 0.8970103391
H -3.6244868931 3.906139871 0.921551179
H -2.375710423 3.1360964948 1.9131755052
H -2.6229438685 2.6166431839 0.22776407
C 0.1131766185 -0.880405033 -3.6175545921
H -0.7384891095 -0.7733641961 -4.3071078095
H 1.0179495904 -0.9109069376 -4.2439245145
C -1.3036572809 -2.8822673913 -3.1497240378
H -1.1879732629 -3.9227985698 -2.810626984
H -1.5263152043 -2.9048513702 -4.2284398925
C -2.4062021424 -2.246526219 -2.3469537534
C -3.7557481148 -2.2741192391 -2.6827327295
H -4.0780685054 -2.7225955277 -3.6249300708
C -4.6809816528 -1.7369137727 -1.7822817783
H -5.743000074 -1.7860531262 -2.0331106621
C -4.2654625436 -1.1383078145 -0.5767426606
C -2.8842788741 -1.1366321343 -0.3411238926
H -2.4434768653 -0.6986957267 0.555503434
C -4.4947403379 0.4404576346 2.0541836955
H -3.6863503729 0.9586669049 1.5049636822
C -3.8385504986 -0.5389682422 3.046008492
H -3.1366300338 -0.0077907072 3.7109816966
H -3.2761907148 -1.3509510466 2.5556359139
H -4.5932159339 -1.0144553712 3.6916808481
C -5.2782566258 1.5281407237 2.8137733541
H -4.6346275185 2.0114610397 3.5680933213
H -6.144781649 1.1089875547 3.3498747129
H -5.6498217003 2.3212564326 2.1478727091
C -6.6067759377 -1.8749953046 1.2383663621
H -7.1699489368 -2.1421506887 0.3236018218
C -7.6318624889 -1.4452446625 2.3047098435
H -8.3365723625 -2.2645540408 2.5230628562
H -8.2297439967 -0.5756023223 1.9877509803
H -7.1385115966 -1.1814804829 3.2545511634
C -5.8298785858 -3.1293652682 1.6739535
H -6.5223410559 -3.9672753399 1.8598032459
H -5.2703490116 -2.9621311299 2.6068559017
H -5.1102985252 -3.4629184714 0.9073603565
C -6.5834927826 0.8961106043 -0.2336077679
H -7.1245751265 1.4059411025 0.5851496363
C -7.6464208947 0.3255918355 -1.1875510386
H -8.2774748189 1.1361464654 -1.5883189125
H -8.3177332945 -0.3945749002 -0.6956084876
H -7.1942753823 -0.1745469764 -2.0608091822
C -5.7096052638 1.9456086791 -0.9418247286
H -6.3291168237 2.7491046525 -1.3735758202
H -5.137728751 1.4962241804 -1.7725446717
H -4.9865783694 2.4189857796 -0.2616409132
C 1.1643121336 -3.0724394079 -3.0662444564
H 1.4467443347 -3.1430002236 -4.1288117002
H 0.8720800214 -4.0786387803 -2.7299731345
C 2.2985668512 -2.5992804012 -2.198594952
C 3.6449879745 -2.832337517 -2.4518584963
H 3.9521252995 -3.3260436238 -3.3763463347
C 4.5858297853 -2.4428859566 -1.4915559092
H 5.6418868606 -2.6487448032 -1.6801626093
C 4.1914550447 -1.7952942694 -0.3073251401
C 2.8146289043 -1.5656097853 -0.1628247305
H 2.3965959969 -1.0499900694 0.7042492381
C 7.1759214151 -1.6026514577 0.4774772063
H 7.1363128245 -2.6207116778 0.0426970294
C 7.6283299841 -0.6318605156 -0.6291286583
H 8.5867547386 -0.9574478006 -1.0657788373
H 7.7881371072 0.3809147333 -0.2265660702
H 6.9017720598 -0.5440319397 -1.4541260547
C 8.2090976717 -1.6471144954 1.6210718704
H 9.2077079683 -1.8968653064 1.2263916108
H 7.9637711382 -2.4003303986 2.3853067563
H 8.2970900626 -0.6739770466 2.1290706204
C 5.0051810437 -2.4312850595 2.5751092289
H 5.733513061 -2.1319880247 3.3518727604
C 5.2616224259 -3.9089375284 2.2296939792
H 5.0994501213 -4.5542324875 3.1091986741
H 6.2895179454 -4.0903910353 1.87992149
H 4.5766554082 -4.2595021036 1.4371070621
C 3.5985440683 -2.2441548726 3.1679490719
H 3.4769688436 -2.8537662041 4.0790091441
H 2.8107949594 -2.5693009344 2.4653440732
H 3.3861423332 -1.1998587629 3.4468269442
C 4.9777862444 0.5280400366 1.5134478432
H 3.9782524517 0.4655519009 1.9820028283
C 4.8530471695 1.4458787772 0.285123003
H 4.5468742614 2.4592733328 0.5876383926
H 4.1036328397 1.0824285745 -0.4381218362
H 5.8073606574 1.5451161377 -0.2531023594
C 5.9327597921 1.126535401 2.5625902224
H 5.5722590439 2.1131588203 2.8988233087
H 6.9426586451 1.2764132043 2.1486961053
H 6.0305289713 0.4910990812 3.4574011454

139
4-4open-2+ 2 4 -4414.5050027 1.207646 0 B3LYP-D3/def2SVP
O 0.1222896631 -1.0250445784 -0.5988250565
O 0.5142743892 -3.2673736817 0.6709615035
C 1.0960990917 -3.7179333308 -0.4603001838
O 1.5657364374 -2.9844816744 -1.2983484288
C 1.1186980735 -5.2244484691 -0.5261426956
H 1.6993956288 -5.6235772403 0.3198326102
H 1.5704623648 -5.544551144 -1.4725484471
H 0.0935135491 -5.6152724885 -0.4401549073
Fe 0.1661026746 -1.4924659022 0.9790139053
N -0.154332524 0.3754833715 1.6196776656
Si -0.5272084366 4.2483447179 0.0216968723
C -0.221918116 1.4355785659 0.78528957
H -0.0673814596 1.1989101669 -0.2683798269
Si 5.4368633321 -0.3197564643 -1.0248879672
N 0.1529725774 -1.9186987253 3.0321487236
C -0.475950439 2.7372376142 1.2264226536
Si -5.2821704224 -1.5367817504 -1.0065842078
N 2.0839901068 -1.2198711575 1.2560092634
C -0.6508931879 2.8904577679 2.6170826316
H -0.8654634433 3.8752356359 3.0390535572
N -1.753823582 -1.8971480217 1.1771893042
C -0.5521760628 1.7989402274 3.4807370097
H -0.6766355054 1.9188908763 4.5594248382
C -0.3034544551 0.5364392702 2.9464691252
C -1.993180151 5.3231284868 0.5898639309
H -1.7331192108 5.5795539135 1.6352225662
C -3.3521711887 4.6057765805 0.6176582608
H -4.11228662 5.2302325129 1.1154863274
H -3.3149539625 3.6413654817 1.1505508422
H -3.7192753697 4.4069571092 -0.4005661725
C -2.0771415666 6.6487469109 -0.1906124438
H -2.8459136917 7.3071958876 0.2461337466
H -2.3575702847 6.4800744505 -1.2427848844
H -1.127143573 7.2059251616 -0.1833411649
C -0.5901127175 3.5218379663 -1.7409353909
H 0.265075215 2.8170900903 -1.7674217109
C -0.321793921 4.5815631191 -2.8267117538
H -0.2688344047 4.111984402 -3.8229580512

H 0.626607482 5.1173556115 -2.6664464359
H -1.1252236125 5.3338705316 -2.8664330245
C -1.8675167176 2.7221581481 -2.0566315578
H -1.7645264149 2.1799925444 -3.0115707534
H -2.7384671635 3.3876920121 -2.1579160902
H -2.1199827662 1.9821188547 -1.2795685773
C 1.1100063955 5.1885806293 0.2936503092
H 1.0587860098 6.0336214952 -0.4180830615
C 1.2506759953 5.7774169183 1.7072097559
H 2.1759474352 6.3711799764 1.7902508267
H 1.3156466566 4.9847721623 2.4731344487
H 0.4153164707 6.4419141424 1.977030576
C 2.3328789214 4.3359048809 -0.0738095451
H 3.2671612692 4.9054663239 0.0590896102
H 2.3088955689 3.9909023759 -1.1175517753
H 2.4101889108 3.4410802851 0.5685587855
C -0.2421964008 -0.7002122893 3.8017651575
H 0.4504260778 -0.543177528 4.6433040834
H -1.2352775338 -0.8650672412 4.2478646819
C 1.5421384148 -2.3659966035 3.3244520362
H 1.6086128733 -3.4274400704 3.0422935236
H 1.779700438 -2.2891811596 4.3970172752
C 2.536247237 -1.6193726119 2.4690408309
C 3.8824225685 -1.4713659811 2.7790232635
H 4.2503681639 -1.7655722941 3.76447554
C 4.7585112759 -0.995639209 1.792663386
H 5.8198886965 -0.9076213273 2.0364972568
C 4.3027913725 -0.6805793274 0.5009517943
C 2.9191219172 -0.779795892 0.3006508197
H 2.4463275654 -0.5530340645 -0.6545170101
C 4.8884234461 -1.6302243689 -2.3003111211
H 3.8334803295 -1.36981404 -2.5112098542
C 4.8901354692 -3.0805339251 -1.7864399369
H 4.4176495497 -3.7474420354 -2.5261868333
H 4.3262828494 -3.1927572381 -0.846545051
H 5.9105003628 -3.4531541044 -1.6130861409
C 5.6718449302 -1.5020452154 -3.6199134345
H 5.2548255745 -2.1752635889 -4.3872014283
H 6.7301721997 -1.7796570417 -3.4883375147
H 5.644576796 -0.480035755 -4.0309578287
C 7.2413083323 -0.4123022302 -0.421866004
H 7.2531625623 0.1894407864 0.5086375292
C 8.224214796 0.2567870015 -1.4038579718
H 9.249870498 0.2249270322 -1.0008542497
H 7.9826101937 1.3132997396 -1.5948144792
H 8.2433397173 -0.2619254817 -2.3756272364
C 7.7151162535 -1.8366719525 -0.0753799021
H 8.6936425823 -1.8082264632 0.4314571869
H 7.8439275877 -2.4417317157 -0.9863177938
H 7.0182688601 -2.3848432018 0.5799426578
C 5.0389989633 1.4351434529 -1.6549044599
H 5.7505378398 1.5759148781 -2.4898592212
C 5.3424860566 2.5047265643 -0.5917052636
H 5.1836919 3.5187155333 -0.9936350185
H 6.381730476 2.4565633743 -0.2318100526
H 4.6825739278 2.3975578735 0.2859411609
C 3.6231887232 1.6004721878 -2.2322503576
H 3.5081930075 2.5923522612 -2.7001885521
H 2.85211969 1.5352006079 -1.4452456972
H 3.3873716008 0.8495273832 -3.0020706454
C -0.8765281092 -2.9912788587 3.1499267578
H -1.1731573676 -3.1602583958 4.1974302894
H -0.4364337683 -3.919558338 2.7601980562
C -2.0535913901 -2.6262711918 2.2794546002
C -3.3627122357 -3.0375249447 2.4980545189
H -3.6105904648 -3.6155822555 3.3909479791
C -4.3430890896 -2.7129645055 1.5508778191
H -5.3676348892 -3.052085503 1.7219902161
C -4.0307495063 -1.9659353407 0.401078939
C -2.6926958365 -1.5707737666 0.274578053
H -2.3354158468 -0.9821852488 -0.5726097832
C -7.0259013615 -1.8941879197 -0.3352650646
H -6.9470406153 -2.9171587566 0.0826290843
C -7.4668184397 -0.9528945911 0.8000094894
H -8.4006428748 -1.3111617501 1.2635303629
H -7.6677210445 0.0611116686 0.4198025366
H -6.7142541325 -0.859547742 1.6011808551
C -8.0889218654 -1.9517781439 -1.4502235447
H -9.0655977746 -2.2493145002 -1.0345083763
H -7.8357906595 -2.6765441293 -2.2392250043
H -8.2280767602 -0.9710475921 -1.9313468995
C -4.8540660921 -2.7228963811 -2.4387099682
H -5.5714125051 -2.4552843909 -3.2371293594
C -5.0977119158 -4.1932454274 -2.0549195469
H -4.8992277862 -4.8602284357 -2.9100664088
H -6.1331109737 -4.3805880208 -1.7311538725
H -4.4293639339 -4.5113751004 -1.2352791295
C -3.4364335887 -2.5275818461 -3.0021177771
H -3.2722214668 -3.1791646578 -3.8761899841
H -2.6619835348 -2.7954239455 -2.2613600017
H -3.245872154 -1.4932940355 -3.3298041418
C -4.9019866276 0.2713682412 -1.4832466311
H -3.8889339467 0.2319123958 -1.9289517586
C -4.8429835835 1.2447097732 -0.2940225967
H -4.5636696964 2.2529322067 -0.634742089
H -4.1028853383 0.941780674 0.4663035646
H -5.8149628935 1.3338608737 0.2134862092
C -5.8542392685 0.7901830655 -2.5770559904
H -5.5231045864 1.7752527632 -2.9460953126
H -6.8778714909 0.9180420132 -2.1906548252
H -5.9078975751 0.115511771 -3.4462484953

139

6-4-2+ 2 6 -4414.5143632 1.2065155 0 B3LYP-D3/def2SVP
O -0.1843946798 -0.7713976441 1.1588334674
O -0.1569488857 -3.2136856039 0.5962232176
C -0.1852661457 -2.9536902559 1.8061452774
O -0.2032555179 -1.6997314088 2.2235612675
C -0.2114908662 -3.9625956046 2.9005941811
H -1.1419680086 -4.5456612557 2.8140067306
H -0.1599333609 -3.4842372864 3.8858867118
H 0.6338869952 -4.6533215689 2.7638088453
Fe -0.1722743265 -1.5098481538 -0.6355935832
N -0.072283355 0.4168105542 -1.4291016217
Si 0.4946008394 4.3406829752 -0.0835603394
C 0.1036118826 1.517595769 -0.6723835342
H -0.0242925123 1.357995355 0.3997969801
Si -5.7185952985 -1.0771848886 1.0165370886
N -0.194216316 -2.0563027259 -2.8610934419
C 0.4302005957 2.7709441291 -1.2011961271
Si 5.3581552494 -1.267345674 1.0946805609
N -2.1842453724 -1.844777009 -1.0443145156
C 0.6141600169 2.8085814488 -2.5970519601
H 0.901891386 3.7415040559 -3.0884772713
N 1.847551109 -1.80359733 -1.0721437222
C 0.424412365 1.6679677551 -3.3854050641
H 0.5459763047 1.7112739048 -4.4701290388
C 0.0556591214 0.4739800746 -2.7697552369
C 1.8864987244 5.4424291566 -0.7690859409
H 1.6013522871 5.5897710322 -1.8290636761
C 3.2780467383 4.7876452467 -0.7524589502
H 4.0028811208 5.3954825309 -1.3188679307
H 3.2788545833 3.775859083 -1.1907692582
H 3.6660770933 4.7019997461 0.2742226364
C 1.9152720703 6.8363340717 -0.114673478
H 2.6467099472 7.4876412273 -0.6208868974
H 2.2135396603 6.7792788997 0.9443835318
H 0.9389863505 7.344313549 -0.1588815095
C 0.6597131286 3.721632996 1.7148199089
H -0.2021138408 3.0359150455 1.8339422171
C 0.4715510584 4.8517590981 2.7446897854
H 0.4571477389 4.4457590358 3.7699344717
H -0.4699782202 5.4035232579 2.597936752
H 1.2947368177 5.5820791789 2.6987944485
C 1.9369581773 2.9162061971 2.0088121681
H 1.8777407898 2.4354360044 2.9999445121
H 2.8238537791 3.5675354946 2.0207736575
H 2.1285257534 2.1243837377 1.2668057965
C -1.2037770526 5.1817802934 -0.3182919661
H -1.1725460602 6.0660605443 0.3449636712
C -1.4263697941 5.6812926624 -1.7555945672
H -2.3933410565 6.2039022361 -1.8432597426
H -1.4503436003 4.845267616 -2.476648319
H -0.646627179 6.3860948309 -2.0836743587
C -2.3650808304 4.2852352165 0.1421593594
H -3.3315009994 4.8040811195 0.0274562751
H -2.2800749345 3.9886963825 1.1994860616
H -2.4249728925 3.3611118552 -0.4587674621
C -0.3521480427 -0.7499068556 -3.565055627
H -1.4225725259 -0.624525123 -3.7962231114
H 0.1741397811 -0.7690259015 -4.5327605912
C -1.3849358132 -2.9228567657 -3.0543915005
H -1.1172634754 -3.9400243433 -2.7261229313
H -1.6769622853 -2.9937333758 -4.1162248412
C -2.5363559842 -2.4461878241 -2.1979956411
C -3.8790443705 -2.6629128259 -2.4947491669
H -4.1636686739 -3.1402861992 -3.43503484
C -4.8486625637 -2.2765153074 -1.5600082564
H -5.9008361268 -2.464356155 -1.7888391776
C -4.4885212915 -1.663067548 -0.3453988154
C -3.1176208086 -1.4599401964 -0.1563090859
H -2.7245228129 -0.9758081853 0.7407625199
C -4.8149482061 -1.370850849 2.6778557237
H -3.9791319567 -0.644586873 2.6601075666
C -4.2015041151 -2.7712638691 2.8591852112
H -3.6151296332 -2.8184725345 3.7932212855
H -3.5312873545 -3.0547776635 2.029425334
H -4.9759461961 -3.5501889382 2.9260827118
C -5.7047396174 -1.0013177093 3.8803271181
H -5.1241835089 -1.0181227298 4.8176723172
H -6.5327869165 -1.7175816696 4.0009784396
H -6.1453956606 0.0038882698 3.7858661678
C -7.3449720607 -2.0253427571 0.7521980552
H -7.5660243384 -1.8816564029 -0.3237801374
C -8.5271048989 -1.4200908396 1.5336239162
H -9.4647566549 -1.9394768766 1.2760369713
H -8.6752071836 -0.3511168117 1.3148789395
H -8.3909124472 -1.5209131181 2.6216457688
C -7.2219395025 -3.5378826004 1.008888151
H -8.1363351785 -4.0646160121 0.6900123504
H -7.0885374728 -3.7501675023 2.0816299672
H -6.3731461091 -3.995657084 0.4732247713
C -5.9575553006 0.7923241405 0.7142189951
H -6.6468262197 1.1241288467 1.5128436742
C -6.6345406706 1.0632577754 -0.6410412911
H -6.8087793709 2.1424998577 -0.7850359025
H -7.6103563442 0.5620943337 -0.733535636
H -6.0038774173 0.7231696593 -1.4814518973
C -4.6568654094 1.6009352979 0.850272462
H -4.8466656052 2.6761664688 0.6998185881
H -3.9118166448 1.303537583 0.0907447983
H -4.1875966604 1.4912586544 1.8408320668
C 1.0804895567 -2.746135812 -3.1757034133
H 1.3642521833 -2.6100061008 -4.2328962659
H 0.9259691386 -3.8274939508 -3.0319460125
C 2.2068119722 -2.3225012072 -2.260160937
C 3.5513108578 -2.5343316956 -2.5581718113
H 3.8398582901 -2.94870487 -3.5268805459
C 4.5142404936 -2.2339679775 -1.5887269348
H 5.565078504 -2.4305550823 -1.814880928
C 4.1490047116 -1.6832021526 -0.3453250752
C 2.7795201567 -1.4681685458 -0.1602792078
H 2.3851281392 -1.0108814725 0.7500403221
C 7.1323546599 -1.5774303027 0.4799235245
H 7.0804299826 -2.5769400643 0.0046159978
C 7.6146653845 -0.573146153 -0.5834544057
H 8.568259362 -0.9042570009 -1.0264450856
H 7.7964167058 0.4179893262 -0.1387126316
H 6.8973159484 -0.4333218578 -1.4091356412
C 8.1523774769 -1.679893795 1.6317101101
H 9.1494894046 -1.9389580043 1.2393369733
H 7.8839611364 -2.4493589299 2.3715241246
H 8.2556239863 -0.724139078 2.1690558846
C 4.9258122853 -2.4902071944 2.5019548167
H 5.6107404656 -2.2049232528 3.322272467
C 5.2329072276 -3.9463676611 2.1086346549
H 5.041489647 -4.631883091 2.95121128
H 6.2805085225 -4.0922769309 1.80532177

H 4.5964173696 -4.2768557867 1.2687475253
C 3.4898114635 -2.3706275086 3.0410050486
H 3.3593383185 -2.9961926675 3.9402086057
H 2.7557031422 -2.7299855437 2.297589014
H 3.2132853231 -1.3406169386 3.3173444165
C 4.9397792794 0.526282658 1.5953266641
H 3.9166001231 0.4685148737 2.0154108437
C 4.8925785701 1.5067604986 0.4101822683
H 4.6045508739 2.5125871802 0.7507176724
H 4.1634961783 1.2025305013 -0.3594641974
H 5.8707529105 1.6031141223 -0.08334095
C 5.8623658228 1.0476596619 2.7123727814
H 5.5118333015 2.0256411631 3.0824086421
H 6.8919857714 1.1913116307 2.347778599
H 5.9076764223 0.3666136138 3.5772845474

139
2a-4-2+ 2 2 -4414.5287402 1.210039 0 B3LYP-D3/def2SVP
O -0.1615472984 -3.5063327776 -0.4973092231
O -0.0667733129 -1.4778387644 1.0077124313
C -0.1700305107 -2.572468783 1.6000033345
O -0.2292233802 -3.6926127392 0.931450918
C -0.2354649759 -2.7061117305 3.0819527116
H 0.6985119978 -2.3083118325 3.5079002419
H -0.3710432304 -3.7510741011 3.3846840437
H -1.0672677089 -2.0873066316 3.4517722769
Fe -0.0328963839 -1.7409699732 -0.9299236918
N 0.102944056 0.144653339 -1.4265641932
Si 0.214986806 3.9338407379 0.3642841269
C 0.1306757482 1.1609746122 -0.5435155743
H 0.0546799824 0.8597577202 0.5021582701
Si -5.5068738575 -0.3494762528 0.7280741954
N -0.0129006721 -2.1344978341 -2.8844478368
C 0.2526056602 2.4998853255 -0.9268561646
Si 5.440911059 -1.2136483732 1.0640299785
N -1.9837025767 -1.5983862566 -1.1829245038
C 0.350022383 2.7275494892 -2.3159201886
H 0.4581358425 3.7454981725 -2.6984922803
N 1.9325514138 -1.874958962 -1.0914799038
C 0.3086725481 1.6721958678 -3.2308041205
H 0.3759350306 1.8568751443 -4.3053529394
C 0.1787097984 0.3708680575 -2.747870787
C 1.4220511474 5.2607328826 -0.2703130177
H 1.0399534681 5.494152323 -1.2832911152
C 2.8729371315 4.7740388909 -0.424833795
H 3.4808130927 5.5220210554 -0.9599444203
H 2.9484156054 3.8255011192 -0.9817585938
H 3.347975646 4.619649332 0.5563769208
C 1.3534549962 6.5655725026 0.5451460754
H 1.9732667262 7.3475474276 0.0765589702
H 1.7346164229 6.42331597 1.5689745925
H 0.329369445 6.9632466686 0.6224556014
C 0.5817015283 3.1433229841 2.0635154244
H -0.1931809056 2.3562048285 2.1548532668
C 0.3597167241 4.1242086348 3.2302374449
H 0.4637413325 3.6082664764 4.1994262491
H -0.6396803451 4.5859867752 3.2102985695
H 1.1005217132 4.9389400478 3.2182980601
C 1.9527065724 2.4574577034 2.1730734318
H 2.0237420974 1.8629602167 3.1000605938
H 2.768966997 3.1945860148 2.2046744746
H 2.1643770959 1.7854430001 1.3266485221
C -1.5724228089 4.6007418259 0.3577776263
H -1.5585555421 5.44184083 1.0753124258
C -1.9980563605 5.1566843098 -1.0112409352
H -3.0136022518 5.5834519686 -0.9633546143
H -2.0204170313 4.3650143662 -1.7805628577
H -1.3285602669 5.9545898971 -1.3686688976
C -2.5793699405 3.5600659355 0.8705013561
H -3.598913464 3.9785395102 0.8945913685
H -2.3514747048 3.2114766801 1.8897729632
H -2.6085817088 2.6757484495 0.2101735635
C 0.105572955 -0.8419805711 -3.6474613528
H -0.7575884761 -0.7403815424 -4.3234220787
H 0.999403744 -0.8790310434 -4.2889487946
C -1.3072968604 -2.8355051964 -3.1384554628
H -1.1922760771 -3.8717755349 -2.7863405252
H -1.5502815509 -2.8672163715 -4.212489363
C -2.3986492148 -2.1883070357 -2.3239279191
C -3.7539185035 -2.2470304795 -2.6336470664
H -4.0859516545 -2.7243406437 -3.5581224618
C -4.6729610582 -1.7092432823 -1.7275181067
H -5.7383595574 -1.788703433 -1.9558903804
C -4.2490210516 -1.0746583739 -0.5425420117
C -2.8644079276 -1.0410773333 -0.3361336777
H -2.4182598123 -0.568452878 0.5401989548
C -4.4564573591 0.4866105951 2.0931880727
H -3.660711508 1.0178429722 1.5377940792
C -3.778706554 -0.5059323802 3.0572693478
H -3.0438674917 0.0107519883 3.6992222915
H -3.2570932008 -1.3276646769 2.5378870338
H -4.5162740631 -0.973528053 3.7277753947
C -5.2320815422 1.5579517338 2.8826706358
H -4.5788303102 2.036903124 3.6316942536
H -6.0863544942 1.1257291407 3.4277468433
H -5.6208985316 2.3562525878 2.2330802244
C -6.5774789416 -1.8232380324 1.2861953919
H -7.1390813509 -2.0926676402 0.3710933692
C -7.6056533822 -1.3967304871 2.3509291165
H -8.3068007093 -2.2190352677 2.5691161869
H -8.2071008343 -0.5298278638 2.0333369005
H -7.1143874187 -1.1308991364 3.3014292857
C -5.7980134249 -3.0756897062 1.7230445248
H -6.4875014151 -3.9188362023 1.8947826405
H -5.2536605212 -2.9118110093 2.6655230278
H -5.0655846894 -3.4012797134 0.9656466685
C -6.5708501621 0.9541007542 -0.1715490003
H -7.1192396376 1.4492226535 0.6515270724
C -7.6235944374 0.380308222 -1.1350201028
H -8.2617519369 1.1873800986 -1.5313793042
H -8.2891390802 -0.351589841 -0.6526848513
H -7.1608307463 -0.1066901541 -2.0101586597
C -5.707281934 2.0196510994 -0.8677191001
H -6.3352279948 2.81790525 -1.2969208791
H -5.1251653247 1.5837913834 -1.698503078
H -4.9946115425 2.4976522827 -0.1803342317
C 1.17511643 -3.0184821758 -3.0752906782
H 1.4586601601 -3.0973998854 -4.1369011781
H 0.8957143213 -4.0230364038 -2.7232936383
C 2.3100918586 -2.5260587241 -2.2144500318
C 3.6534371175 -2.7795025771 -2.4660236359
H 3.9528191956 -3.2969274713 -3.3799962033
C 4.601461049 -2.3853848576 -1.51497005
H 5.6539639491 -2.6115086563 -1.7007560557
C 4.2195334401 -1.710805442 -0.3413532928
C 2.8463582446 -1.4608733015 -0.2009034161
H 2.4411195443 -0.9210704104 0.6577865671
C 7.2107597937 -1.5120786645 0.4335586964
H 7.18057744 -2.5360009176 0.0121845818
C 7.6429081371 -0.5502331818 -0.6887370161
H 8.6016146945 -0.8705576791 -1.1285513226
H 7.7945291108 0.469409733 -0.3005472115
H 6.9090871422 -0.4820793927 -1.5091280601
C 8.2524003667 -1.5280216504 1.5704288113
H 9.2472905073 -1.791149361 1.1752330669
H 8.0117237031 -2.2580752065 2.3583003296
H 8.3464718148 -0.5412838033 2.0497862498
C 5.0442577979 -2.3921883893 2.5175193961
H 5.7085837648 -2.0523156385 3.3338633923
C 5.4007002606 -3.852220835 2.1851389193
H 5.2162709673 -4.5106499363 3.0502657078
H 6.4572589961 -3.9755414512 1.904017946
H 4.7880660949 -4.2347726484 1.3499083931
C 3.596026142 -2.2956015675 3.0281326694
H 3.4646809776 -2.8989990606 3.9422506124
H 2.8879734755 -2.6966618704 2.2807687409
H 3.2924396021 -1.2633160144 3.2694169101
C 5.0060955893 0.5875074557 1.5135371753
H 4.0081735115 0.516606483 1.9851969018
C 4.8769125895 1.5227015147 0.2985492884
H 4.5794378335 2.5339358542 0.6160119016
H 4.12139919 1.1731952424 -0.4250381613
H 5.8283523405 1.6232290583 -0.2444336349
C 5.9628635638 1.1753387994 2.5671812918
H 5.5955804569 2.1520782294 2.9243024859
H 6.9676426397 1.343184709 2.14814834
H 6.0746419399 0.5245690113 3.449175928

139
4a-4-2+ 2 4 -4414.5096673 1.2074569 0 B3LYP-D3/def2SVP
O -0.0559395582 -3.4428686924 -0.332373166
O -0.0920092773 -1.2011289416 -1.5292546863
C -0.1755801082 -2.2033127382 -2.2683637803
O -0.1768914815 -3.4120870752 -1.7677287067
C -0.2896111862 -2.1385852642 -3.7518112949
H -1.2240169788 -1.6110172336 -4.0009203669
H -0.2904753777 -3.1382096073 -4.2021218278
H 0.5491793061 -1.5406235796 -4.1399530779
Fe -0.012603091 -1.7918585972 0.4177093799
N 0.0299080759 -0.0548010125 1.2942129246
Si 0.1012070133 3.9625070518 0.1078347131
C 0.0366557442 1.0916210907 0.5838530349
H 0.0270327402 0.961946585 -0.4997389237
Si 5.7145880006 -0.5345976778 -0.7000453948
N -0.0007670737 -2.5679345289 2.46679723
C 0.0460471438 2.3557417669 1.1777211162
Si -5.6743992036 -0.5517493005 -0.7843858151
N 2.0396117395 -1.9173576422 0.8000516604
C 0.0520062108 2.3604989734 2.5879115703
H 0.0551508173 3.3078544834 3.1327042282
N -2.0640567843 -1.8568790325 0.8520100567
C 0.0506464134 1.1702652365 3.319913803
H 0.0559556965 1.1847637074 4.412292459
C 0.038625277 -0.0478771012 2.6406944173
C -0.8236382733 5.2939828554 1.1026001321
H -0.2924664907 5.3123238784 2.0744346572
C -2.297056722 4.9498566489 1.3839391532
H -2.7354718428 5.6647531992 2.0994215607
H -2.4281306704 3.9386362258 1.803857656
H -2.9019425884 5.0052519182 0.4653965459
C -0.6835592269 6.7015213058 0.4928395798
H -1.1331106551 7.4569046108 1.1579955143
H -1.2012203746 6.7771272208 -0.4765089758
H 0.3671780334 6.990447895 0.3337959636
C -0.6020625339 3.4931912445 -1.6041859918
H 0.0732079812 2.6841467857 -1.9462024983
C -0.473421744 4.6418872622 -2.6224183993
H -0.7546160258 4.3017574426 -3.6330671245
H 0.5512972004 5.0406073291 -2.6836694495
H -1.1399762583 5.4807128277 -2.3679818821
C -2.0285073297 2.9239544264 -1.5898448636
H -2.295112036 2.5075591015 -2.5763890935
H -2.7726538382 3.7012639158 -1.3611030719
H -2.1621767834 2.1261743616 -0.8421997927
C 1.9482203736 4.4035624088 -0.078245822
H 1.9548843693 5.3741327415 -0.6077127312
C 2.6472450349 4.5992979134 1.277728437
H 3.6953836738 4.9119067545 1.138794378
H 2.6678794782 3.6620413648 1.8609574198
H 2.1588930983 5.3693778697 1.8955718335
C 2.6986174446 3.3851062279 -0.9502706145
H 3.7605841358 3.6606605761 -1.0557050065
H 2.2857885022 3.3082317678 -1.967724088
H 2.6705943538 2.3781792733 -0.4985107984
C 0.0355361141 -1.3797934803 3.366296093
H 0.9300417778 -1.4304742771 4.0082827333
H -0.8289076363 -1.4064046091 4.0493681137
C 1.221159867 -3.3996018718 2.5327871581
H 1.0084135875 -4.3423773679 2.0042828464
H 1.4904738242 -3.6569222445 3.5714619617
C 2.3717164454 -2.720861378 1.8256831649
C 3.7100253064 -2.9445409066 2.1424260847
H 3.973749901 -3.6020643539 2.9738684645
C 4.6962340197 -2.3262394794 1.3701250112
H 5.7450901632 -2.524210757 1.6039676052
C 4.3587952746 -1.456106022 0.3135008813
C 2.9880412732 -1.293843797 0.0797767886
H 2.6140782883 -0.6439449832 -0.7152947511

C 4.8040160207 0.4055421131 -2.0922344211
H 3.9586110645 0.896200529 -1.5739832438
C 4.2194661387 -0.5036614648 -3.1888873778
H 3.5598015871 0.0704201366 -3.8626978426
H 3.6368048362 -1.3497717958 -2.7857870423
H 5.0162732478 -0.934567549 -3.8146730608
C 5.6502370451 1.5334902423 -2.7122331515
H 5.0622307062 2.0946591804 -3.4580786599
H 6.5386456486 1.1399475541 -3.2306209571
H 5.99932091 2.2576439366 -1.9604964125
C 6.9581881856 -1.8667564895 -1.2520371838
H 7.4119040703 -2.2116598852 -0.3029224599
C 8.0894935409 -1.26761117 -2.108879242
H 8.8743637679 -2.0182434887 -2.2980207482
H 8.5732106895 -0.4027951071 -1.626818798
H 7.7170025733 -0.9368201936 -3.0921594664
C 6.3296876551 -3.0969236804 -1.9296977532
H 7.0937454765 -3.8710288892 -2.1105233118
H 5.8904936646 -2.845537502 -2.9072315126
H 5.5363591316 -3.5562590377 -1.317137223
C 6.5343261789 0.7091164419 0.4959339005
H 7.2307181937 1.278586423 -0.1476241186
C 7.3684354688 0.0555587798 1.6102361057
H 7.8908880697 0.8240522172 2.2037425645
H 8.1366172814 -0.6302059181 1.221139129
H 6.7353603018 -0.5082953953 2.3166875985
C 5.5162223579 1.7016486424 1.0828644374
H 6.0159654623 2.4482066557 1.72244083
H 4.7688461981 1.1860343466 1.7118546382
H 4.9693056238 2.2530318006 0.3053713669
C -1.2477451404 -3.3558173576 2.574234676
H -1.4988467783 -3.5912850469 3.6226116731
H -1.0802497275 -4.3117811039 2.0533566338
C -2.3948342657 -2.652943614 1.8853206686
C -3.7310733875 -2.8563487612 2.2200945491
H -3.9925323984 -3.4968077879 3.0654911728
C -4.7215436634 -2.2464887198 1.4439716904
H -5.7692375358 -2.4279840542 1.6947316393
C -4.3866494972 -1.4076836082 0.3648803221
C -3.0143163709 -1.2395255567 0.1318661724
H -2.643870772 -0.5885612958 -0.664506857
C -7.4051771003 -0.8635651086 -0.0585758755
H -7.4220589827 -1.9498382553 0.1573697735
C -7.6649590223 -0.1125895172 1.2607503235
H -8.6097298778 -0.4467966649 1.7199826909
H -7.7594302249 0.9712251535 1.0880139015
H -6.8660454313 -0.2558072413 2.0072362872
C -8.5346039643 -0.5916136371 -1.0725228725
H -9.5119960347 -0.8591744612 -0.6384848577
H -8.4213279779 -1.1715765718 -2.0011282223
H -8.5853293095 0.4727227801 -1.3498391807
C -5.4990283264 -1.4054990364 -2.4870347404
H -6.2122841374 -0.8646546107 -3.136816675
C -5.926848031 -2.8831920933 -2.4313358565
H -5.8778100171 -3.3469920593 -3.4306235892
H -6.9565051311 -3.011648004 -2.0652748016
H -5.2643153458 -3.4674864148 -1.7689246207
C -4.1038885514 -1.2737420746 -3.12319834
H -4.10811235 -1.6686513606 -4.1533535198
H -3.3573207303 -1.8637308325 -2.5615027636
H -3.7495721886 -0.2309431755 -3.1729351799
C -5.1374334537 1.2764372115 -0.8890570768
H -4.2087296526 1.2477217456 -1.4875414226
C -4.7914460378 1.9150607641 0.4677086501
H -4.4373965391 2.9497442623 0.3328391158
H -3.9976163704 1.3665185855 1.0018730386
H -5.6653363014 1.9581733732 1.1348925189
C -6.1492805379 2.138154145 -1.6666108649
H -5.7404698912 3.1454204747 -1.8534248736
H -7.0842386218 2.2704133848 -1.0993961694
H -6.4122739689 1.7047750171 -2.6448650582

139
6a-4-2+ 2 6 -4414.5079571 1.2065803 0 B3LYP-D3/def2SVP
O -0.1240916657 -3.2814488509 0.4919929643
O -0.1309468634 -0.9633884114 1.3953592803
C -0.1138032529 -1.8650587653 2.2541523443
O -0.1095804972 -3.1269331358 1.8881166104
C -0.0935978982 -1.6373510161 3.723320515
H 0.8025704293 -1.0478946705 3.9714412773
H -0.0870016166 -2.5856405385 4.2737873614
H -0.9788517473 -1.0434404588 3.9982452772
Fe -0.1312469519 -1.6802734326 -0.579950621
N -0.0269622635 0.3053045878 -1.2600712559
Si 0.4666758034 4.1974600228 0.1890964252
C 0.1254722572 1.3927258228 -0.48178613
H 0.0028994917 1.2076973497 0.5857830284
Si -5.7695430195 -0.8134823818 0.8474335982
N -0.1557684668 -2.1474052916 -2.7755507839
C 0.4236311581 2.6661707495 -0.9790485964
Si 5.4778102829 -1.1830338593 0.9645864345
N -2.1765383661 -1.7999167407 -1.0570930625
C 0.5983884337 2.7436016866 -2.3738175452
H 0.8584617881 3.6951584776 -2.8444121215
N 1.9152292831 -1.8436661863 -1.0793280508
C 0.4385212732 1.6162929723 -3.186173666
H 0.5601044101 1.6875762596 -4.2695449708
C 0.1032193674 0.3975912956 -2.5979040322
C 1.7009685454 5.4350291788 -0.5609252938
H 1.3280731059 5.5795299187 -1.5937503221
C 3.1412634737 4.9042068231 -0.6593708167
H 3.7717322455 5.590535155 -1.2484059725
H 3.1976587323 3.9113173909 -1.1353119128
H 3.6047682023 4.8198815787 0.3361229056
C 1.6557673317 6.8129645266 0.1258380491
H 2.292972488 7.5343235114 -0.4116724832
H 2.0288282537 6.7625049619 1.1610360927
H 0.6392191417 7.2355240679 0.1583134608
C 0.8424234142 3.5277619212 1.9387780414
H 0.0080918863 2.8240376256 2.1288152708
C 0.7559610745 4.6227760801 3.0182874602
H 0.8428021687 4.186111353 4.0273853802
H -0.1950580293 5.1769006402 2.9815323984
H 1.5711103435 5.3559832556 2.914027012
C 2.1529238399 2.7330548198 2.0598081875
H 2.2178872234 2.2253667879 3.0378601797
H 3.0310996881 3.3916500115 1.985612713
H 2.2622600571 1.965945793 1.2767180584
C -1.3068284035 4.9033351836 0.1427308352
H -1.2826513373 5.7811457982 0.8146787443
C -1.7005779531 5.3946762896 -1.2606683882
H -2.7107947256 5.8364582452 -1.252758267
H -1.7190843867 4.5654889832 -1.9897094838
H -1.013842373 6.1643636598 -1.6458942288
C -2.3460545674 3.9139889973 0.6947624242
H -3.359278658 4.3467651152 0.6587892083
H -2.149885283 3.6352545707 1.7424058608
H -2.3769563102 2.9844576853 0.099861699
C -0.2448011812 -0.8127733155 -3.4473688673
H -1.2877949721 -0.6731087478 -3.7740632429
H 0.367636289 -0.8134448201 -4.3627416297
C -1.3776575322 -2.9607486977 -3.0125548772
H -1.1668374732 -3.988862044 -2.6765685188
H -1.6317949212 -3.0154352391 -4.0848977954
C -2.5295081137 -2.4233921818 -2.1952665159
C -3.8738013824 -2.5997105873 -2.5150991799
H -4.1578623685 -3.0988222123 -3.444160486
C -4.8434782023 -2.1439884752 -1.6151124754
H -5.8972530267 -2.3041965335 -1.8567007868
C -4.4837503067 -1.4911346583 -0.4185236736
C -3.1100829448 -1.3385954732 -0.2080216794
H -2.7181278915 -0.8323446379 0.6770710326
C -4.8106499537 -0.5386158351 2.4799230138
H -3.9858706492 0.1420094584 2.1872355144
C -4.180304544 -1.8027842691 3.0909667295
H -3.5369390671 -1.5414984357 3.9500137154
H -3.568492665 -2.3762382309 2.3736579537
H -4.9490400761 -2.4903917783 3.474265371
C -5.6531782014 0.2150059406 3.5268696323
H -5.0441165372 0.4690559814 4.4107196765
H -6.4964518917 -0.3969632212 3.8828583949
H -6.0690487712 1.1559397982 3.1343141904
C -7.2000144376 -2.0672690225 0.8757967525
H -7.5379742139 -2.0937370399 -0.1787278935
C -8.3960774266 -1.5830532582 1.7168476261
H -9.2533691279 -2.2658888026 1.5988761671
H -8.7371564275 -0.5763970513 1.4274805572
H -8.1513193929 -1.5573418629 2.7906770216
C -6.7836133145 -3.4972574396 1.2624895642
H -7.6159935569 -4.2016017441 1.1001722779
H -6.5120291754 -3.5642277787 2.3274162073
H -5.9232717209 -3.8634072024 0.6777058694
C -6.3471413239 0.8676713486 0.1543309578
H -7.0833200556 1.2416262284 0.8898060799
C -7.0642468849 0.730974147 -1.1994265513
H -7.4272291325 1.7108724517 -1.5511523928
H -7.9371660552 0.0618126366 -1.1483140651
H -6.3869054036 0.3425032742 -1.9802171495
C -5.1980865243 1.8859323335 0.0762432794
H -5.5541305997 2.8552615011 -0.3107047749
H -4.3993035345 1.5434915519 -0.6057454248
H -4.7369504643 2.0796155492 1.0570185145
C 1.0909153629 -2.8860141675 -3.0978115206
H 1.3321438851 -2.8226048265 -4.172402483
H 0.9174753108 -3.9508539381 -2.8733550869
C 2.248113025 -2.4186194407 -2.2473130896
C 3.5852243971 -2.6395915999 -2.5708615227
H 3.8512965301 -3.1008105544 -3.5246411923
C 4.569676844 -2.2872096718 -1.6416160948
H 5.6155162955 -2.4910884161 -1.8833384622
C 4.2320072591 -1.6769056775 -0.418175147
C 2.8653227478 -1.4608634225 -0.2103772193
H 2.4957211107 -0.962876512 0.6893002467
C 7.2337446371 -1.5605999523 0.3379188915
H 7.1591862043 -2.5820225504 -0.0844396147
C 7.7088391192 -0.6170176995 -0.7832212824
H 8.6551589465 -0.9768743507 -1.2192596531
H 7.9009223392 0.395610179 -0.3944738769
H 6.982737651 -0.5174027284 -1.6070808392
C 8.2730877493 -1.6190292836 1.4754346538
H 9.2604121373 -1.9063840189 1.0780911532
H 8.0107644475 -2.3510617743 2.2543183994
H 8.3951199788 -0.6405298546 1.9659247669
C 5.0578493256 -2.2860982126 2.4708857171
H 5.7858393831 -1.9675447043 3.240409143
C 5.3007187219 -3.7766606528 2.172367888
H 5.1274291303 -4.3934060665 3.0699044148
H 6.3287170345 -3.9776752508 1.8348721722
H 4.6161144988 -4.1444526579 1.388263769
C 3.6510444783 -2.0778881062 3.0604196697
H 3.5471717451 -2.6211170756 4.0147570311
H 2.8722785098 -2.4801865955 2.3879961108
H 3.4236219657 -1.0172802351 3.2599826388
C 5.1024463868 0.6481645142 1.3506834398
H 4.1088856589 0.6248858972 1.8371904567
C 4.9803240696 1.5430624286 0.1051693574
H 4.7176189431 2.5732406782 0.3913801517
H 4.2020195885 1.189821417 -0.5918542907
H 5.9247561728 1.596565716 -0.4564789567
C 6.0922477205 1.2422138254 2.3695508209
H 5.7657779657 2.2456133762 2.6907927839
H 7.0977530856 1.3550421515 1.9339759089
H 6.1905552359 0.623223795 3.2757855343

References

1 Borrell, M. & Costas, M. Mechanistically Driven Development of an Iron Catalyst for Selective Syn-Dihydroxylation of Alkenes with Aqueous Hydrogen Peroxide. *J. Am. Chem. Soc.* **139**, 12821-12829, (2017).

2 Serrano-Plana, J. *et al.* Trapping a Highly Reactive Nonheme Iron Intermediate That Oxygenates Strong C—H Bonds with Stereoretention. *J. Am. Chem. Soc.* **137**, 15833-15842, (2015).

3 Lyakin, O. Y., Zima, A. M., Samsonenko, D. G., Bryliakov, K. P. & Talsi, E. P. EPR Spectroscopic Detection of the Elusive FeV═O Intermediates in Selective Catalytic Oxofunctionalizations of Hydrocarbons Mediated by Biomimetic Ferric Complexes. *ACS Catal.* **5**, 2702-2707, (2015).

4 Becke, A. D. Density-functional thermochemistry. III. The role of exact exchange. *J. Chem. Phys.* **98**, 5648-5652, (1993).

5 Lee, C., Yang, W. & Parr, R. G. Development of the Colle-Salvetti correlation-energy formula into a functional of the electron density. *Phys. Rev. B* **37**, 785-789, (1988).

6 Griffith, J. C. *et al.* Alkene Syn Dihydroxylation with Malonoyi Peroxides. *J. Am. Chem. Soc.* **132**, 14409-14411, (2010).

7 Miehlich, B., Savin, A., Stoll, H. & Preuss, H. Results obtained with the correlation energy density functionals of becke and Lee, Yang and Parr. *Chem. Phys. Lett.* **157**, 200-206, (1989).

8 Stephens, P. J., Devlin, F. J., Chabalowski, C. F. & Frisch, M. J. Ab Initio Calculation of Vibrational Absorption and Circular Dichroism Spectra Using Density Functional Force Fields. *J. Phys. Chem.* **98**, 11623-11627, (1994).

9 Grimme, S., Antony, J., Ehrlich, S. & Krieg, H. A consistent and accurate ab initio parametrization of density functional dispersion correction (DFT-D) for the 94 elements H-Pu. *J. Chem. Phys.* **132**, 154104, (2010).
